# DELP-YOLOv12: a lightweight deployable model for maize pest and disease detection

**DOI:** 10.1186/s13007-026-01527-4

**Published:** 2026-03-31

**Authors:** Jie Shi, Xinrui Zhang, Zhi Li, Linlin Yang, Xiuying Tang, Wencai Yang

**Affiliations:** Faculty of Mechanical and Electrical Engineering, Yunnan Agricultural University, Kunming, 650201 China

**Keywords:** Maize pest and disease detection, Lightweight model, YOLOv12, Attention mechanism, Model pruning, Edge computing

## Abstract

**Background:**

Maize is one of the world’s most important cereal crops for both food and feed, yet its yield is severely threatened by pests and diseases. The complexity of field environments, the variability of illumination, and the diversity of pest and disease types make accurate detection a challenging task. Although existing maize pest and disease detection models have achieved substantial progress, they still face difficulties in small-object recognition, feature perception, and efficient deployment on edge devices. To address these limitations, this study proposes a deployable and lightweight detection framework, DELP-YOLOv12, based on the YOLOv12 architecture.

**Results:**

A Dynamic RepConvBlock with NAM (DRN) module was designed and integrated into the C3k structure to form C3k_DRN, enabling multi-branch training and inference-time fusion to enhance both feature representation and computational efficiency. Additionally, a Lightweight Feature Enhancement Detection Head (LFEDH) was developed to refine feature extraction for small-scale pests and irregular lesions. To further improve feature discrimination, an Efficient Channel Attention (ECA) mechanism was incorporated to highlight lesion- and pest-related responses while suppressing background noise. Considering edge-device constraints, a Layer-Adaptive Sparsity for Magnitude-based Pruning (LAMP) strategy combined with fine-tuning was applied to compress the model while maintaining accuracy. Experimental results on the maize pest and disease dataset demonstrate that DELP-YOLOv12 achieved a precision of 94.1%, a recall of 89.6%, and an mAP50 of 94.2%, outperforming the baseline across all metrics. Meanwhile, the parameter count and computation cost were reduced by approximately 68% and 60%, respectively, while preserving real-time inference capability on embedded hardware such as the NVIDIA Jetson Orin NX.

**Conclusions:**

The proposed DELP-YOLOv12 effectively balances accuracy, efficiency, and deployability for field-based maize pest and disease detection. Its integration of DRN, LFEDH, and LAMP modules enhances recognition of small and irregular targets while maintaining low computational demand, offering a practical solution for real-time agricultural monitoring and intelligent pest management.

**Supplementary Information:**

The online version contains supplementary material available at 10.1186/s13007-026-01527-4.

## Introduction

Maize (Zea mays L.) is one of the world’s three major staple crops, with a total global output of approximately 1.229 billion tons in 2024. As a crucial food and feed source, maize plays an essential role in ensuring food security, maintaining feed supply, and serving as an important raw material for bio-industrial production. However, throughout its growth period, maize is highly susceptible to infections caused by fungi, bacteria, and insect pests, such as leaf spot, rust, borers, and fall armyworms. These pests and diseases not only damage leaves and stems but also cause severe yield losses and quality deterioration at different growth stages, posing a potential threat to regional and national food security [1]. Consequently, developing automated techniques capable of rapidly and accurately identifying maize pests and diseases under complex field conditions is of vital importance for precision control and yield preservation [2].

Traditionally, pest and disease monitoring in maize fields has relied on manual inspection and laboratory testing. Manual observation is time-consuming, labor-intensive, and subject to individual bias, while laboratory diagnosis offers high accuracy but is unsuitable for large-scale and continuous field monitoring. Early computer vision studies often depended on handcrafted feature extraction—such as color, texture, and shape descriptors—combined with conventional classifiers for disease identification. Although these approaches achieved satisfactory accuracy under controlled conditions, they were highly sensitive to lighting variations, occlusion, and background complexity, and their manually designed features lacked generalizability across diverse field environments.

With the advancement of machine learning (ML) in agricultural image analysis, supervised algorithms such as Support Vector Machines (SVM) [3], Random Forests (RF) [4], and their ensemble methods have been widely adopted to replace or enhance traditional visual features. For instance, Alehegn addressed the inefficiency and low precision of manual maize disease diagnosis by extracting color, texture, and shape features and integrating them with KNN and ANN classifiers, achieving an accuracy of approximately 94.4% across three disease classes and healthy leaves [5]. Restrepo-Arias et al. developed the SNMPF model, which achieved 98.4% accuracy in plant disease identification [6]. Alzoubi proposed a PRF-SVM ensemble model that fused probabilistic response functions with support vector machines to enhance accuracy and robustness in multi-class fig leaf disease detection [7]. Li et al. applied K-means segmentation combined with KNN classification for early, non-destructive detection of maize leaf spot and rust diseases, avoiding chemical contamination during diagnosis [8].

Machine learning methods exhibit strong interpretability and low training complexity in small-sample conditions, and when feature engineering is carefully designed, they can achieve high classification performance. However, they still rely heavily on high-quality handcrafted features or complex preprocessing steps. Their scalability to multi-class, multi-scale, and highly cluttered field scenarios remains limited, often requiring extended pipelines for practical deployment.

Over the past five years, deep learning (DL)—especially convolutional neural networks (CNNs) [9, 10]—has revolutionized end-to-end object detection and classification in crop pest and disease recognition. One-stage detectors such as YOLO have gained widespread adoption due to their real-time performance. Bachhal et al. integrated PRF with SVM to enhance maize leaf disease recognition accuracy, achieving approximately 96.7% classification precision [11]. Askale et al. developed and deployed a VGG16-based deep CNN model for real-time maize leaf disease detection and classification on mobile devices, demonstrating high accuracy and practical feasibility [12]. Zhang et al. improved recognition accuracy and robustness by introducing a Multi-path Activation Function (MAF) module into CNNs, achieving strong performance on public datasets [13]. Yang et al. proposed a real-time pest detection approach based on deep CNNs, attaining superior accuracy and inference speed on a self-built dataset while maintaining low computational cost [1]. Zhang et al. designed a lightweight AgriPest-YOLO model for pest detection in light-trap images, balancing accuracy and speed with reduced model size [14]. Shi et al. applied YOLOv5 and YOLOv8 for maize pest and disease detection, achieving high accuracy, and further deployed YOLOv8 on edge devices [15, 16].Building upon these advances, recent studies have explored hybrid modeling strategies and alternative sensing paradigms for agricultural disease detection. Hybrid CNN–Transformer architectures have been proposed to jointly exploit local feature extraction and global context modeling, demonstrating improved robustness under complex field conditions [17]. In addition, graph neural network (GNN) and hypergraph-based vision models have been introduced for early pest and disease detection by explicitly modeling relational structures among visual features [18, 19]. Beyond vision-based approaches, optimization-driven deep transfer learning frameworks [20], electronic-nose systems combined with clustering analysis for disease profiling [21], and blockchain-enabled crop monitoring frameworks [22] have also been explored to extend the scope of intelligent agricultural monitoring.

Despite the progress achieved by machine learning and deep learning methods, several unresolved challenges remain evident in the literature. Small targets [23] and irregular lesions [24] are common in field imagery, and conventional features or detection heads struggle to maintain high recall for extremely small or partially occluded objects. In traditional machine learning approaches, reliance on handcrafted features and predefined segmentation strategies makes performance highly sensitive to illumination variation, background clutter, and scale changes, limiting their applicability to multi-class and multi-scale field scenarios. Deep CNN-based models alleviate manual feature design but often prioritize classification accuracy over fine-grained localization, resulting in inaccurate bounding box regression when lesion boundaries are blurred or pest instances are densely distributed. Although one-stage detectors such as YOLO achieve real-time performance, their generic feature pyramids and detection heads are typically optimized for natural images, which can lead to missed detections or unstable localization for tiny pests and morphologically irregular disease symptoms. More recent hybrid CNN–Transformer and graph-based models enhance global context modeling or relational reasoning, but this improvement is often accompanied by increased architectural complexity, higher computational overhead, and greater memory consumption, limiting their suitability for deployment on resource-constrained edge devices. In addition, many existing studies are evaluated on datasets collected from limited plots or single growth stages, leaving model robustness under varying phenological stages, complex illumination, and severe occlusion insufficiently validated. Collectively, these limitations indicate that current approaches still struggle to simultaneously achieve accurate small-object detection, precise localization, and efficient real-time deployment in realistic maize field environments.

To address the difficulty of recognizing small pests and irregular lesions in maize field images and to enable efficient edge deployment, this study proposes a deployable lightweight detection model named DELP-YOLOv12, built upon the YOLOv12 framework [25]. The model focuses on enhancing the representational capability for small and morphologically irregular targets while maintaining efficient inference on embedded devices. It consists of three key modules.The first is the Dynamic RepConvBlock with NAM (DRN), which adopts a multi-branch architecture during training to enrich feature representation and employs structural re-parameterization during inference to merge branches into a single convolution, eliminating additional computational overhead. This concept is consistent with the ‘training–inference decoupling’ paradigm exemplified by DBB (Diverse Branch Block) [26] and RepAdapter [27]. By integrating NAM (Normalization-based Attention Module) [28], DRN strengthens the response to critical lesion textures and local morphological features, alleviating the feature weakening of small objects in complex backgrounds. DRN can be embedded into C3k to form C3k_DRN, improving detection of multi-scale and structurally irregular targets.The second module, Lightweight Feature Enhancement Detection Head (LFEDH), reinforces local information representation prior to the classification and regression branches. Through group convolutions and an improved feature fusion strategy, it focuses on fine-scale structures and irregular lesion patterns, enhancing localization and classification precision with minimal inference cost.The third module introduces the Efficient Channel Attention (ECA) mechanism [29] into key feature layers, adaptively amplifying pest- and lesion-relevant channel responses while suppressing redundant background noise, thereby improving discriminability at negligible computational expense.

These modules are integrated into a unified engineering pipeline combining model training, LAMP (Layer-Adaptive Sparsity for Magnitude-based Pruning) [30], and fine-tuning. Systematic comparative and ablation experiments are conducted on both public and self-built maize pest and disease datasets, followed by end-to-end deployment verification on embedded platforms such as NVIDIA Jetson Orin Nano. Results indicate that DELP-YOLOv12 substantially improves small-object recall and overall mAP while reducing parameter count and FLOPs, fully meeting the real-time detection requirements of in-field agricultural applications.

## Materials and methods

**Fig. 1:**
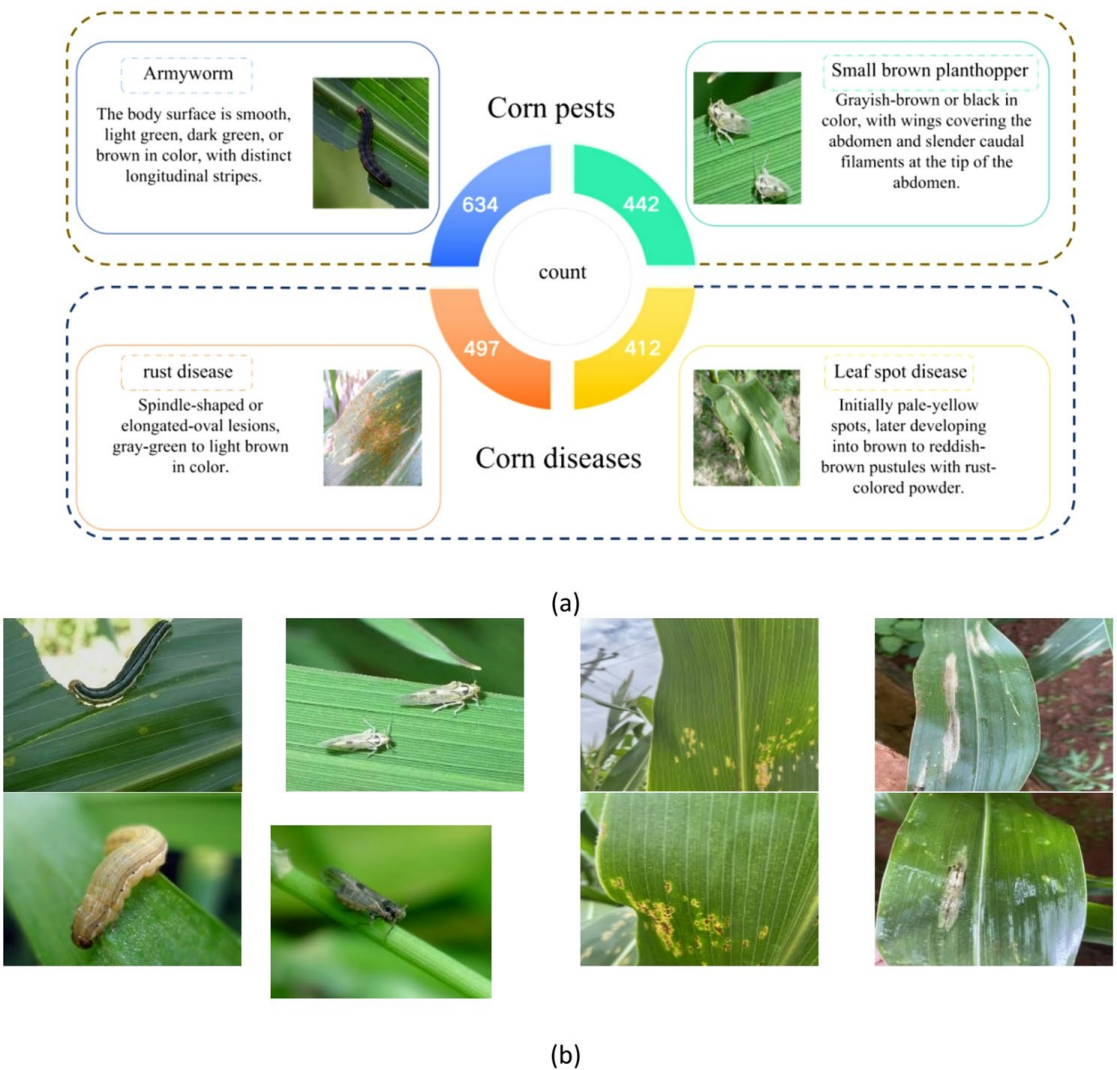
Overview of the maize pest and disease dataset: **a** class composition and sample distribution; **b** representative image samples of different pest and disease categories

Maize is highly susceptible to various pests and diseases during its growth process. To ensure yield and quality, this study focuses on detecting four common types of maize pests and diseases—fall armyworm (Spodoptera frugiperda), gray planthopper (Laodelphax striatellus), leaf spot, and rust—under complex field conditions. A maize pest and disease detection dataset was constructed by combining publicly available images with field-acquired samples.The constructed dataset contains four categories, including two pest classes and two disease classes.Specifically, the dataset includes 634 instances of fall armyworm, 442 instances of gray planthopper, 497 instances of rust disease, and 412 instances of leaf spot disease, resulting in a total of 2005 annotated instances.The per-class distribution and representative visual characteristics of each category are illustrated in Fig. 1.

Because maize is cultivated in open-field environments, plants are exposed to diverse meteorological and illumination conditions. To account for these variations, field image acquisition was conducted during the morning, noon, and afternoon periods at different shooting distances. This approach was designed to capture the influence of environmental variability on image collection, thereby improving the dataset’s diversity and representativeness. Consequently, the original images exhibit highly non-uniform spatial resolutions due to the combined use of different acquisition devices, varying shooting distances, and the integration of publicly available image sources. A statistical analysis based on the dataset configuration file indicates that the image widths range from 168 to 4032 pixels, while the heights range from 116 to 4032 pixels. In addition to a large number of high-resolution images, predominantly at resolutions such as 3024 × 4032 and 3024 × 3024 pixels, the dataset also contains lower-resolution images (e.g., 220 × 165 pixels), reflecting heterogeneity in imaging conditions, acquisition setups, and data sources. This wide resolution distribution increases the diversity of visual scales and poses additional challenges for robust feature extraction under real-world field conditions.

In practical applications, variations in illumination intensity, inter-leaf occlusion, and the similarity between lesion textures and background patterns can interfere with the extraction of discriminative features for maize pest and disease identification. To enhance the robustness of the model under complex field conditions, several data augmentation operations were applied to the original images. These included geometric transformations—rotation, translation, scaling, and flipping—to increase spatial diversity; illumination disturbances achieved by adjusting brightness, contrast, and saturation to simulate different times of day and weather conditions; noise interference such as Gaussian and salt-and-pepper noise to improve the model’s tolerance to image quality degradation; and random occlusion to simulate overlapping leaves and enhance the model’s robustness when part of the target is missing. These augmentation operations effectively expanded the dataset while maintaining authenticity and improved the model’s generalization capability in complex environments. These augmentation operations were applied only to the training set to ensure methodological clarity and unbiased evaluation.

Subsequently, the collected images were annotated using the LabelImg tool. In the labeling process, maize fall armyworm was marked as “N”, gray planthopper as “H”, rust disease as “X”, and leaf spot disease as “B”. The category and location information were saved in YOLO format (.txt) files required by YOLOv12. In the YOLO annotation format, each line of a label file corresponds to one object instance and consists of five elements: the class index and the normalized coordinates of the bounding box center $({\mathrm{x}}_{\mathrm{c}},{\mathrm{y}}_{\mathrm{c}})$, as well as its width and height $(w,h)$. All bounding box coordinates were normalized by the corresponding image width and height, ensuring consistency across images with different spatial resolutions.

The dataset was first divided into a training set and a validation set at a ratio of 8:2, and data augmentation was applied exclusively to the training set, while the validation set remained unchanged. 

In this study, an independent test set was not explicitly constructed, and model performance was evaluated using the validation subset. This experimental setting is consistent with a number of engineering-oriented agricultural vision studies, where the validation set is used for both model selection and quantitative evaluation. For example, in tomato insect infestation detection, the dataset was partitioned into training and validation subsets using an 8:2 ratio without a separate test set [31]. In contrast, large-scale plant image analysis tasks, such as the work of Umirzakova et al. [32] based on UAV imagery, typically adopt a three-way split including training, validation, and test sets [32].

**Fig. 2:**
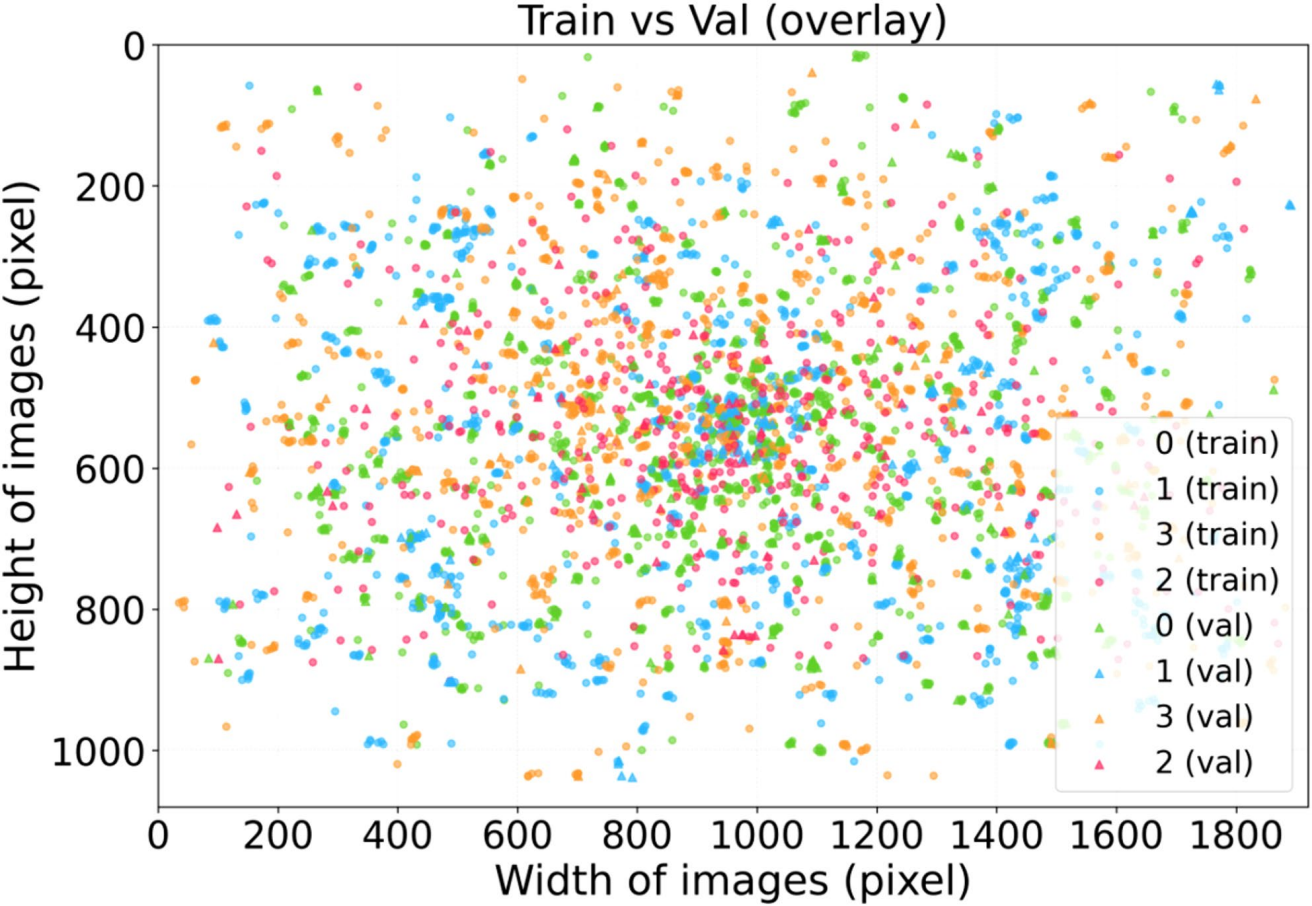
Spatial distribution of object center locations in the training and validation subsets, illustrating the consistency and uniformity of target distributions across the image plane

Figure 2 presents the spatial distribution of target centers in the train and val subsets. It can be observed that pest and disease targets of all categories are uniformly distributed within the images, with minimal spatial variation across classes. The consistent distribution patterns between the train and val subsets indicate that the dataset partitioning maintains both spatial uniformity and balance.

In terms of the experimental environment, model training was primarily conducted on a high-performance GPU workstation, with the detailed hardware and software configurations listed in Table 1. To further evaluate the real-time deployment performance of the improved model on edge devices, the NVIDIA Jetson Orin NX platform was selected as the deployment test environment.

This platform is equipped with 1,024 NVIDIA Ampere CUDA cores and 32 Tensor Cores, providing up to 100 TOPS of AI computing power. It is well suited for rapid inference and embedded deployment of lightweight deep learning models. On this platform, a graphical user interface (GUI) was implemented using PySide6 to support end-to-end operations, including image acquisition, detection, and visualization of results.

**Table 1:** Experimental environment configuration

Environment	Configuration
CPU	Intel i5-12600KF
GPU	NVIDIA RTX 4070 S
Operating system	Windows 11
Development tools	PyCharm 2025 + Python 3.9.20 + PyTorch 2.5.1
CUDA version	CUDA 12.4

## Research method

**Fig. 3:**
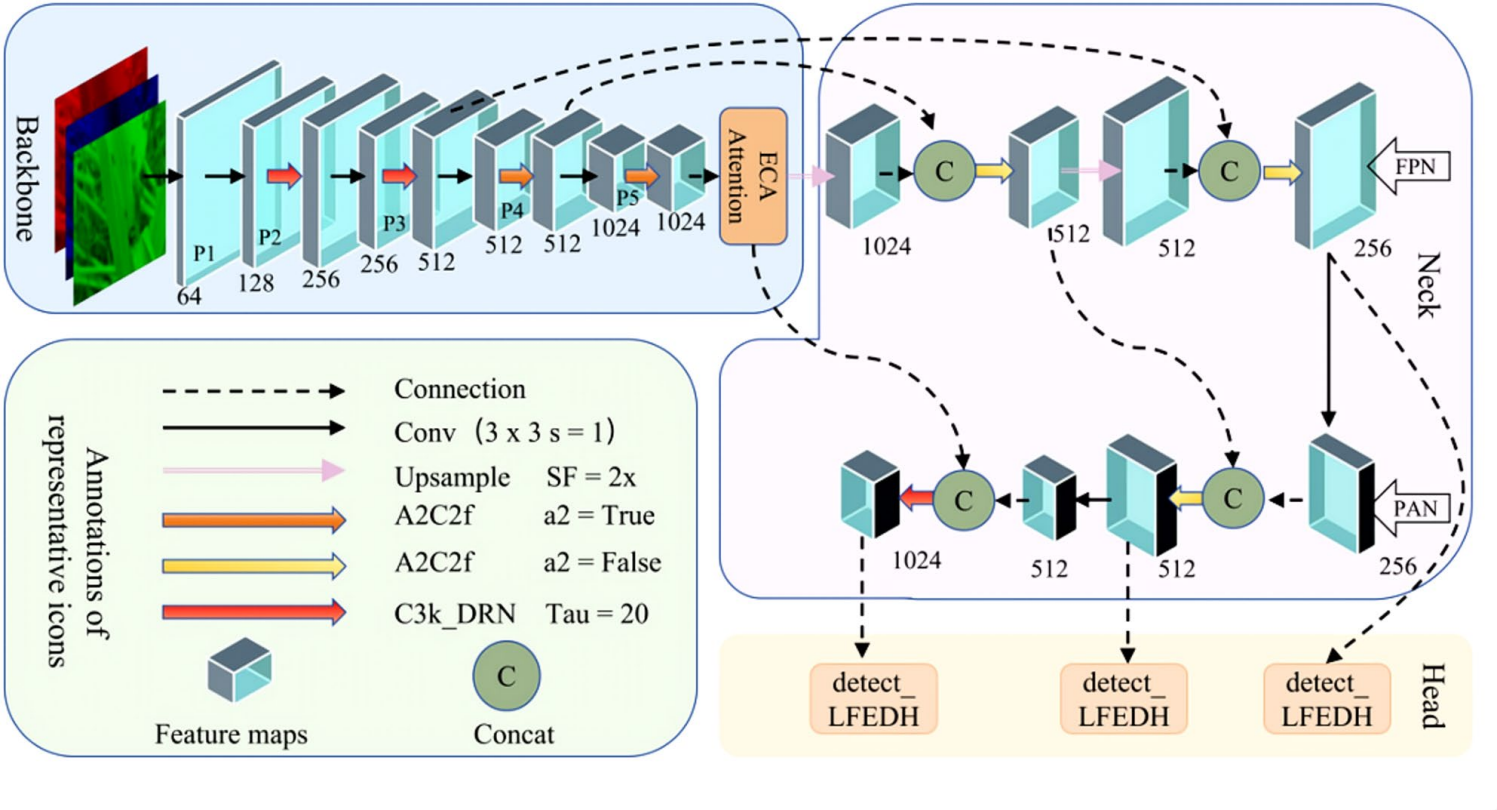
Network architecture of the DEL-YOLOv12 and DELP-YOLOv12 models

To achieve efficient detection and edge deployment of maize pests and diseases under complex field environments, this study proposes the DELP-YOLOv12 model, which introduces targeted improvements to the YOLOv12 framework. The modifications focus on three major aspects: feature enhancement, detection head optimization, and model compression. The overall architecture of the DEL-YOLOv12 (unpruned) and DELP-YOLOv12 models is illustrated in Fig. 3. The proposed design integrates four key components: the Dynamic RepConvBlock with NAM (DRN) module, the Lightweight Feature Enhancement Detection Head (LFEDH), the Efficient Channel Attention (ECA) mechanism, and the Layer-Adaptive Sparsity for Magnitude-Based Pruning (LAMP) strategy.

To address the difficulty of recognizing small pest targets and irregular lesion regions, the DRN module is embedded within the backbone network. This module combines the principles of structural re-parameterization and attention mechanisms. During training, a multi-branch convolutional structure is adopted to enhance feature representation, while during inference, all branches are fused into a single equivalent convolution, achieving a balance between expressive power and computational efficiency. Specifically, DRN employs depthwise and pointwise convolutions as its core computational units, and incorporates the Normalization-Based Attention Module (NAM) to strengthen the selective response to key lesion textures and local morphological patterns. This design effectively mitigates the weakening of small-target features caused by complex background interference.

To overcome the insufficient sensitivity of conventional detection heads to local textures and morphological variations of pest and disease targets, a Lightweight Feature Enhancement Detection Head (LFEDH) is introduced. The LFEDH replaces standard convolutions with group convolutions, which maintain computational efficiency while significantly reducing parameter redundancy. The grouped structure enhances the model’s ability to capture fine-grained local features, enabling more precise representation of lesion textures and pest contours on maize leaves. In addition, the Efficient Channel Attention (ECA) mechanism is independently embedded to adaptively model inter-channel dependencies, thereby improving the model’s capacity to focus on semantically significant features.

To address the constraints of limited computational power and redundant parameters on edge devices, a Layer-Adaptive Sparsity for Magnitude-Based Pruning (LAMP) strategy is employed to compress the trained network. Unlike traditional global pruning methods, LAMP adaptively determines the pruning rate according to the sparsity of weight distributions across layers, allowing deeper feature extraction layers and detection heads to retain sufficient representational capacity during compression. After pruning, fine-tuning is performed to restore model accuracy, ensuring that detection performance remains comparable to or even exceeds that of the unpruned model, while substantially reducing both parameter count and computational load.

Considering the practical deployment requirements of agricultural applications, the DELP-YOLOv12 model was ultimately implemented as an end-to-end real-time detection system on the NVIDIA Jetson Orin NX platform after training and pruning. A graphical user interface (GUI) was developed using PySide6 to enable an integrated workflow for image acquisition, detection, and visualization of results.

The research framework of DELP-YOLOv12 follows a complete design chain of “feature enhancement-detection optimization-lightweight compression-embedded deployment,” effectively balancing detection accuracy under complex field conditions with real-time performance on embedded platforms, and providing an efficient and scalable technical solution for intelligent monitoring of maize pests and diseases.

### C3K_DRN feature extraction module

To enhance the backbone’s ability to represent small targets and irregular lesions in maize field images while maintaining lightweight and efficient inference during deployment, a C3K_DRN feature extraction module is proposed. In engineering implementation, the module follows the “bottleneck stacking + 1 × 1 mapping” design principle of the C3 structure commonly used in the YOLO series, but replaces the conventional bottleneck block with a Bottleneck_DRN unit that contains a dynamic re-parameterization branch (DynRepNam). This modification enables stronger representational capacity during training while preserving single-branch efficiency during inference.

**Fig. 4:**
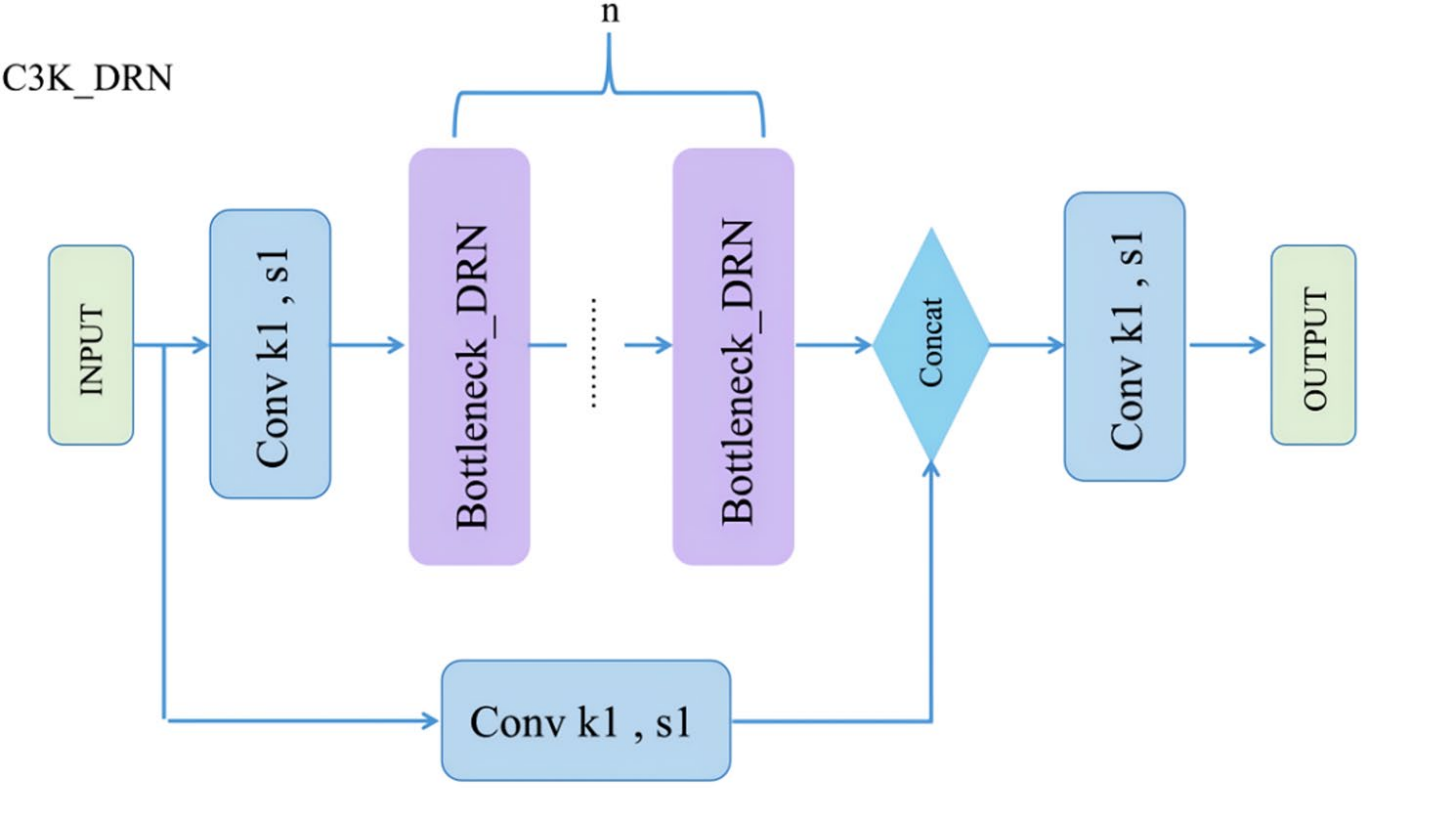
Design of the C3K_DRN feature extraction unit

From input to output, the overall processing flow of the module is as follows: a 1 × 1 convolution is first applied for dimensional transformation, followed by n stacked Bottleneck_DRN units for feature extraction, and finally another 1 × 1 convolution is used for feature fusion and output. The schematic diagram of the module is shown in Fig. 4.

In the DynRepNam module, the input features first pass through a 1 × 1 convolution for channel adjustment and are then simultaneously fed into two parallel sub-branches. One branch is a RepConvBlock that performs structural re-parameterization, while the other is an attention branch based on normalization, namely the Normalization-based Attention Module (NAM).

**Fig. 5:**
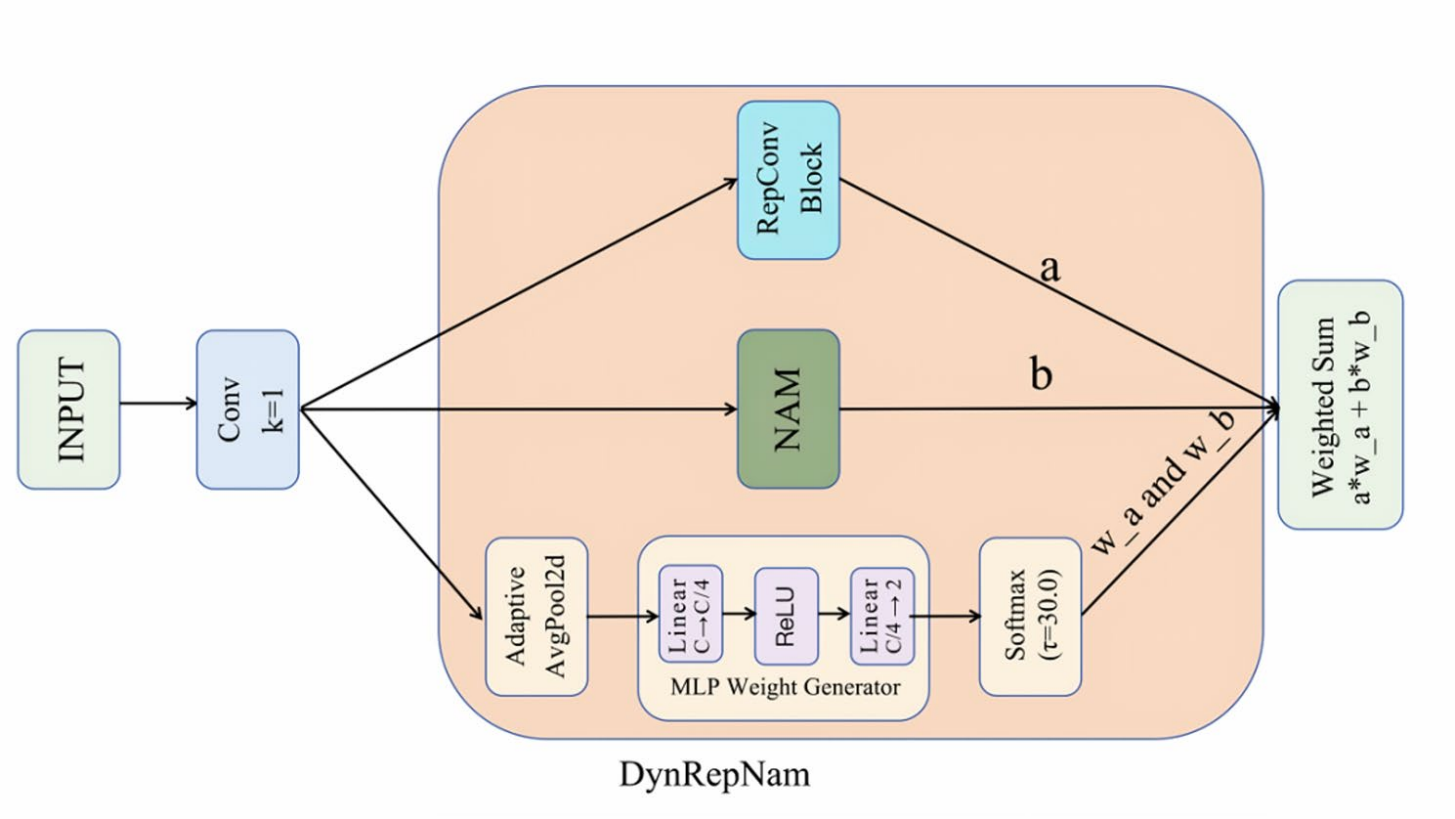
Schematic diagram of the DynRepNam module structure

To adaptively determine the relative contribution of the two paths under different samples and scenarios, a lightweight weight generator is incorporated into the module, as illustrated in Fig. 5.

Specifically, let the input be denoted as $X \in R^{{(N \times C \times H \times W)}}$. The input features are first processed by a 1 × 1 convolution to adjust channel dimensions, followed by adaptive average pooling over the spatial domain to obtain a channel-wise statistical vector y. This vector is then passed through two fully connected layers (MLP) with an intermediate dimension of $\mathrm{max}\left(\frac{\mathrm{c}}{\mathrm{4}}\mathrm{,1}\right)$ and ReLU activation, yielding a two-dimensional scoring vector. The vector is scaled by a temperature parameterτand normalized via the softmax function to produce two per-sample weights, as expressed in Eq. (1).

1 $${\mathrm{w}} = {\mathrm{Softmax}}\left( {\frac{{\mathrm{s}}}{\tau }} \right){\text{ = }}\left[ {{\mathrm{w}}_{{\mathrm{a}}} {\mathrm{,w}}_{{\mathrm{b}}} } \right] \in {\mathrm{R}}^{{{\mathrm{(N}} \times {\mathrm{2)}}}}$$

The resulting weights ${\mathrm{w}}_{\mathrm{a}}$ and ${\mathrm{w}}_{\mathrm{b}}$ are subsequently broadcast to a shape of [N,1,1,1] and used to perform element-wise weighting on the outputs of the two branches. The final output of the DynRepNam module can thus be formulated as Eq. (2):

2 $$Dyn\mathrm{Re} pNam(X) = w_{a} \otimes a(X) + w_{b} \otimes b(X)$$

where a(X) and b(X) denote the outputs of the RepConvBlock and NAM branches, respectively.

The key design aspect of this module lies in the per-sample dynamic weighting mechanism. Differences in global channel statistics among input images drive the weight generator to adaptively adjust the relative contributions of the RepConvBlock and NAM branches. This dynamic weighting enables the module to exhibit strong adaptability and robustness to variations in illumination, background complexity, and object scale commonly encountered in maize field environments.

In maize pest and disease detection tasks, the model must capture fine-grained details of small-scale pest targets while maintaining sensitivity to the complex texture patterns of disease lesions, all under limited computational resources. To this end, the RepConvBlock is adopted as one branch of the DynRepNam module. Its design follows the concept of Diverse Branch Block (DBB, CVPR 2021) [26] and aligns with the “adaptive branch + inference fusion” paradigm emphasized in RepAdapter (arXiv 2023) [27]. This design strengthens feature representation during training and converts to a single-branch convolution during inference, achieving both expressiveness and lightweight efficiency.

**Fig. 6:**
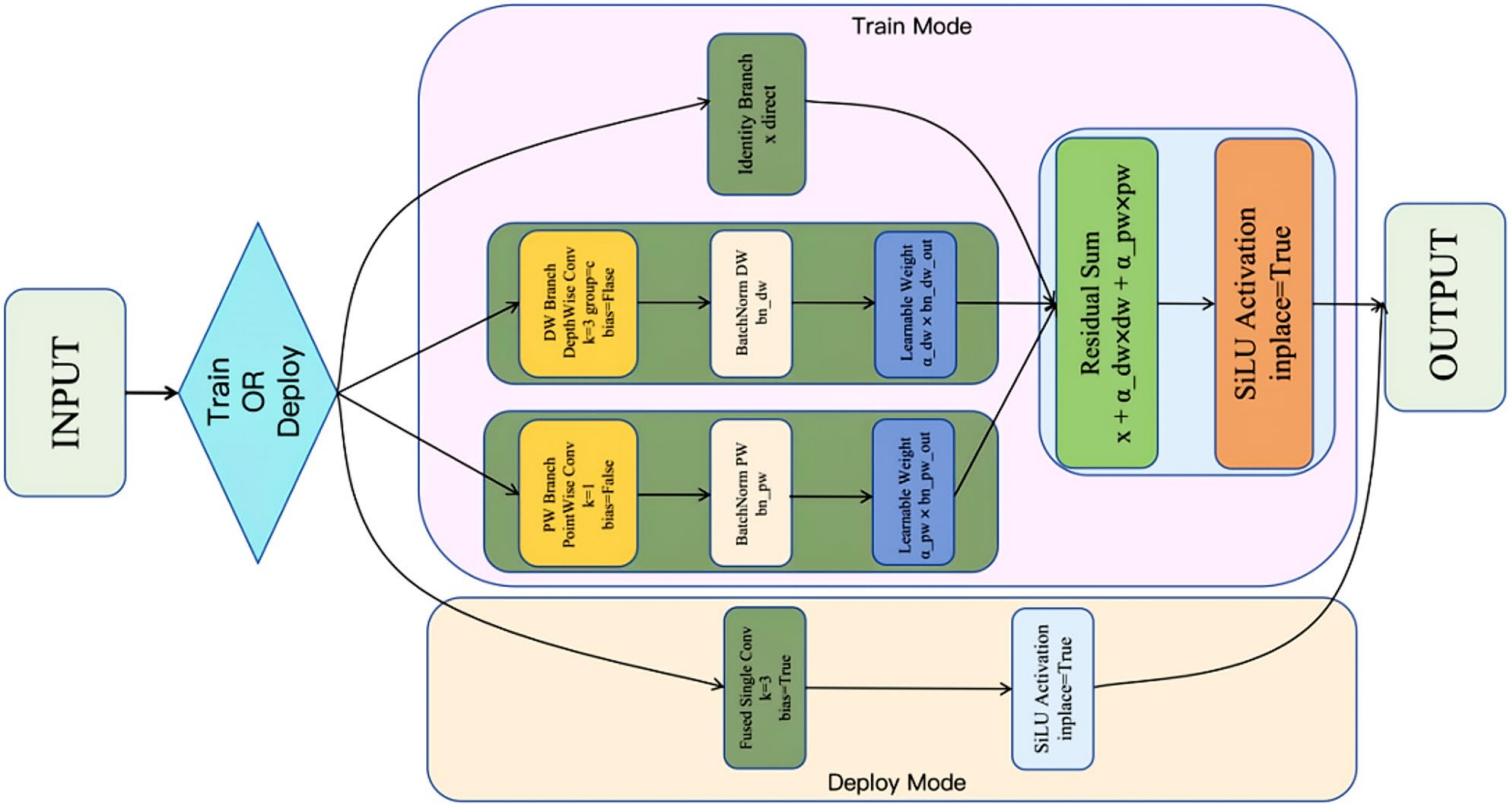
Schematic diagram of the RepConvBlock module structure

As illustrated in Fig. 6, the RepConvBlock operates in a three-branch configuration during training: (i) a Depthwise 3 × 3 convolution + Batch Normalization (BN) branch for extracting local spatial details; (ii) a Pointwise 1 × 1 convolution + BN branch for modeling inter-channel correlations and enhancing feature compactness; (iii) an Identity branch for direct information flow and stable gradient propagation.

The convolutional branch outputs are each scaled by learnable factors after BN and summed with the identity branch to form a residual structure, followed by a SiLU activation for non-linear transformation. This multi-branch design enables the network to learn richer texture, morphological, and scale representations during training, thereby improving adaptability to complex backgrounds and diverse pest–disease appearances in maize fields.

During inference, the RepConvBlock fuses its convolution and BN parameters (fusion process), combining the depthwise, pointwise, and identity mappings into a single equivalent 3 × 3 convolution kernel and bias, thus converting the three training branches into a single deployment branch, as formulated in Eq. (3):

3 $$W_{{eq}} = \alpha _{{dw}} W_{{dw}} + \alpha _{{pw}} W_{{pw}} + I$$

where $\mathrm{I}$ represents the central convolution kernel corresponding to the identity mapping. The final output can then be expressed as Eq. (4):

4 $$b_{{eq}} = a_{{dw}} b_{{dw}} + a_{{pw}} b_{{pw}}$$

This design allows the RepConvBlock to fully exploit the representational power of multi-branch learning during training while maintaining high efficiency with a single-convolution structure during deployment. Unlike conventional multi-branch modules, this approach is particularly suitable for embedded maize pest and disease detection systems: the enhanced representational ability during training facilitates the accurate recognition of small pest bodies and irregular lesions, whereas the lightweight single-convolution structure during inference significantly reduces computational cost and ensures real-time detection speed on edge devices such as the Jetson Orin NX.

To further enhance the model’s sensitivity to lesion textures and small pest targets, the NAM (Normalization-based Attention Module) [28] is incorporated into the DynRepNam structure. The core idea of NAM is to highlight locally salient texture regions based on spatial variance normalization. As illustrated in Fig. 6, the input to NAM is the feature map $\mathrm{X}$. For each channel, the spatial variance along dimensions $\left(\mathrm{H,W}\right)$ is computed, as expressed in Eq. (5):

5 $$Var(X) = \frac{1}{{H \times W}}\sum\limits_{{i = 1}}^{H} {\sum\limits_{{j = 1}}^{W} {\left( {X_{{i,j}} 0\mu } \right)} } ^{2}$$

where $\mu _{c}$ denotes the mean value of each channel. The feature map is then normalized accordingly, as expressed in Eq. (6):

6 $$X_{{norm}} = \frac{X}{{\sqrt {Var(X) + \in } }}$$

where $\epsilon$ is a stability constant introduced to ensure numerical robustness.

This normalization operation suppresses regions with small variance—typically corresponding to redundant information—and highlights spatial areas exhibiting larger fluctuations, which represent key lesion textures and pest features.

Subsequently, a learnable scaling parameter γ is applied to adaptively adjust the normalized features across different channels, as formulated in Eq. (7):

7 $$X' = \gamma \odot X_{{norm}}$$

Finally, the NAM module adds the normalized feature map ${\mathrm{X}}^{{\prime}}$ to the original input $\mathrm{X}$ to form a residual output, as expressed in Eq. (8):

8 $$NAM(X) = X + X^{\prime }$$

The intuitive significance of this design lies in its adaptive response to spatial variations within feature channels.

When a channel exhibits strong fluctuations across the spatial dimension—such as along pest texture edges or lesion boundaries—the normalized magnitude becomes relatively large, and after scaling by γ, the corresponding response is significantly enhanced.

Conversely, for background or homogeneous regions (e.g., healthy leaf areas), the spatial variance is small, leading the normalized result to approach zero, thereby producing only limited enhancement.

**Fig. 7:**
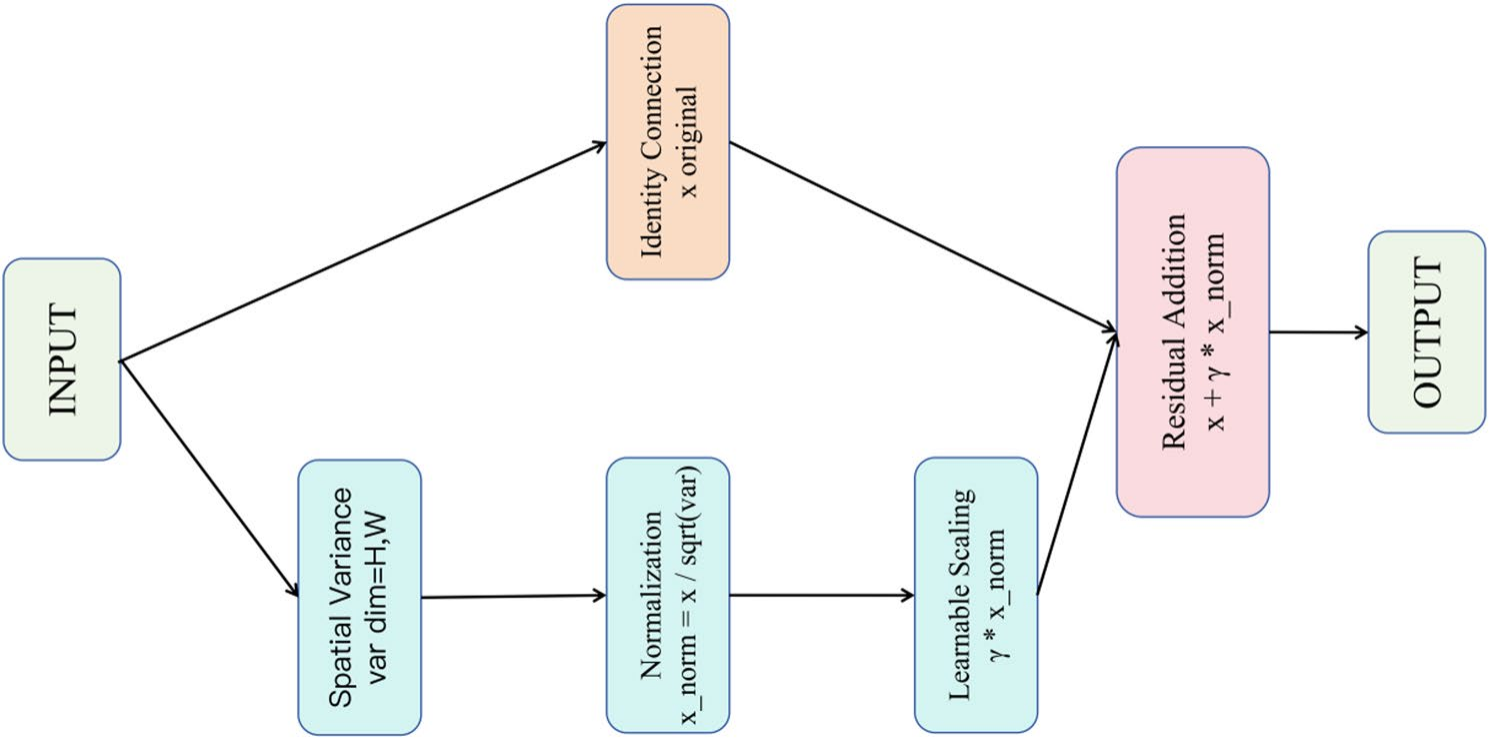
Schematic diagram of the NAM module structure

As a result, the NAM module can adaptively emphasize the fine texture details of leaf lesions and pest bodies while suppressing background interference, effectively improving the robustness and accuracy of maize pest and disease detection under complex field conditions (Fig. 7).

### LFEDH detection head (lightweight feature enhancement detection head)

In an object detection network, the design of the detection head directly affects both the model’s sensitivity to small targets and its inference efficiency.Considering that pest bodies in maize field images are generally small in scale and that lesion edges are complex and morphologically irregular, a Lightweight Feature Enhancement Detection Head (LFEDH) is proposed in this study. The LFEDH enhances the detection head’s ability to represent texture and local features while maintaining a lightweight structure suitable for efficient inference.

The core innovation of the LFEDH lies in introducing Group Convolution to replace the conventional standard convolution.This substitution significantly reduces parameter size and computational cost while maintaining detection accuracy, enabling lightweight inference without compromising performance. Group convolution maintains the receptive field size while effectively reducing redundant computation through channel partitioning, making it particularly suitable for efficient multi-scale feature modeling in maize pest and disease detection tasks.

To further meet the detection requirements of targets at different scales, the LFEDH adopts a scale-differentiated grouping strategy across the three detection branches—P3, P4, and P5.

In the P3 branch, which focuses on small objects, a lower number of groups is used to preserve fine-grained details such as pest bodies and lesion textures.

In contrast, the P5 branch, which targets larger objects, employs more groups to reduce computational overhead.This design achieves a dynamic balance between accuracy and efficiency across feature maps of different resolutions.Let the input feature be denoted as $\mathrm{X}$.The Group Convolution operation divides the input channels into GGG groups, where each group performs convolution independently.The output feature map can be expressed as Eq. (9):

9 $$Y^{{(g)}} = W^{{(g)}} *X^{{(g)}} ,\quad g = 1, \ldots ,G$$

where ${\mathrm{X}}_{\mathrm{g}}$ and ${\mathrm{W}}_{\mathrm{g}}$ denote the input feature and convolution kernel of the g-th group, respectively.The final output is obtained by concatenating the channel-wise results of all groups.

when G = 1, the operation degenerates into a standard convolution, whereas when $\mathrm{G=}{\mathrm{C}}_{\mathrm{in}}$, it becomes equivalent to a depthwise convolution.In the proposed LFEDH detection head, the differentiated design of the G values across scales enables a balance between detection accuracy and inference efficiency on different feature branches.

**Fig. 8:**
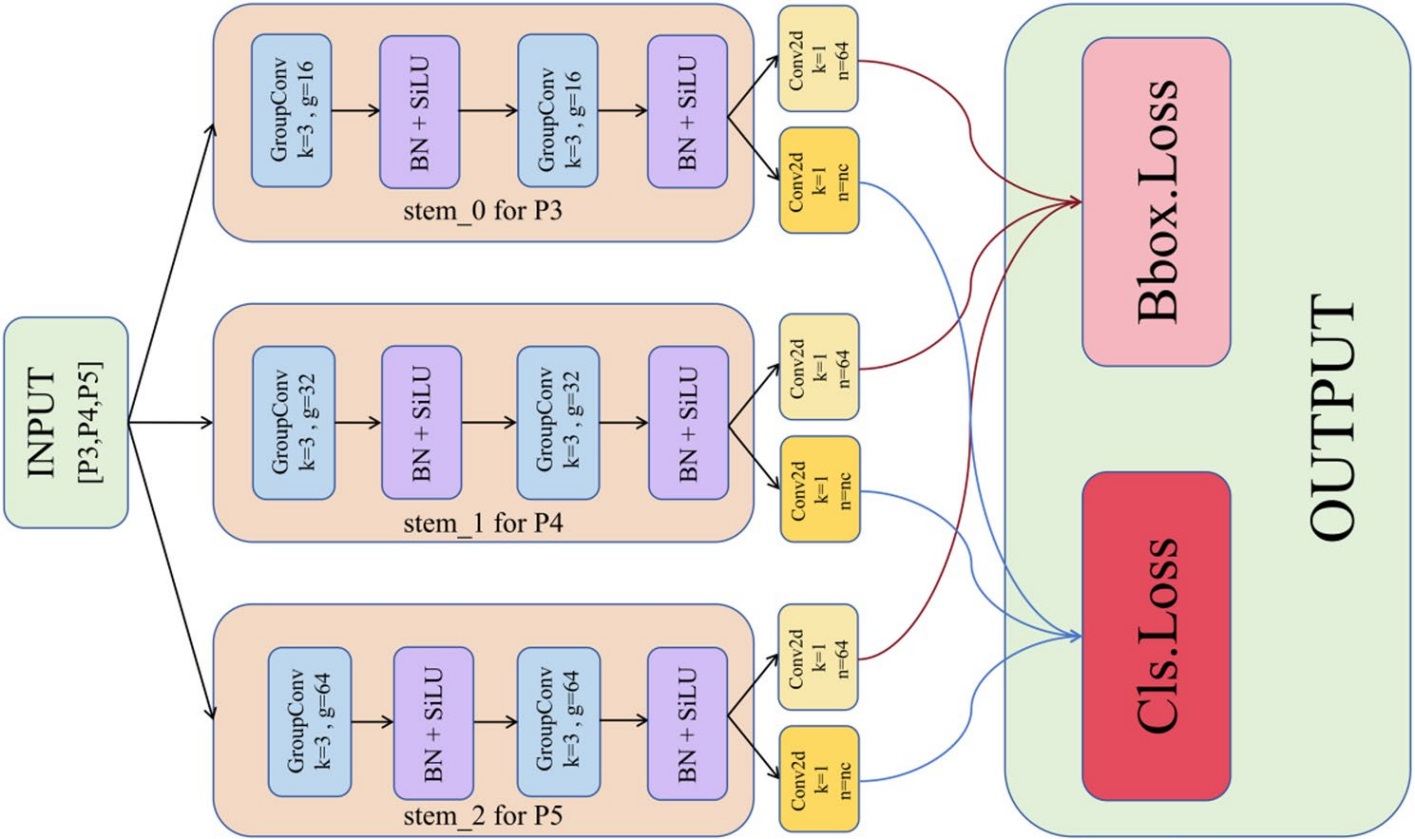
Structural diagram of the LFEDH detection head

As illustrated in Fig. 8, the LFEDH receives multi-scale features [P3,P4,P5] from both the backbone and neck networks.Each scale-specific input is processed through a lightweight Stem module, which consists of Group Convolution (Group Conv), Batch Normalization (BN), and a SiLU activation function.The SiLU function preserves non-linear expressiveness while ensuring smooth gradients, thereby enhancing training stability.

The LFEDH detection head significantly improves the recognition capability for small targets and irregular lesions in field conditions while maintaining a lightweight structure and real-time performance.This design provides an efficient and practical solution for intelligent monitoring in complex agricultural environments.

### ECA attention mechanism

In maize pest and disease detection, targets are typically small, exhibit fine-grained textures, and often have low contrast against complex backgrounds. Spatial features extracted solely through convolution are easily disturbed, resulting in weak responses along lesion boundaries and pest contours. To address this limitation, an Efficient Channel Attention (ECA) mechanism is integrated into the detection head to strengthen the network’s channel-wise feature selection. By adaptively emphasizing channels relevant to pest and lesion information, the ECA module enhances the model’s sensitivity to texture and structural details while suppressing background interference.

**Fig. 9:**
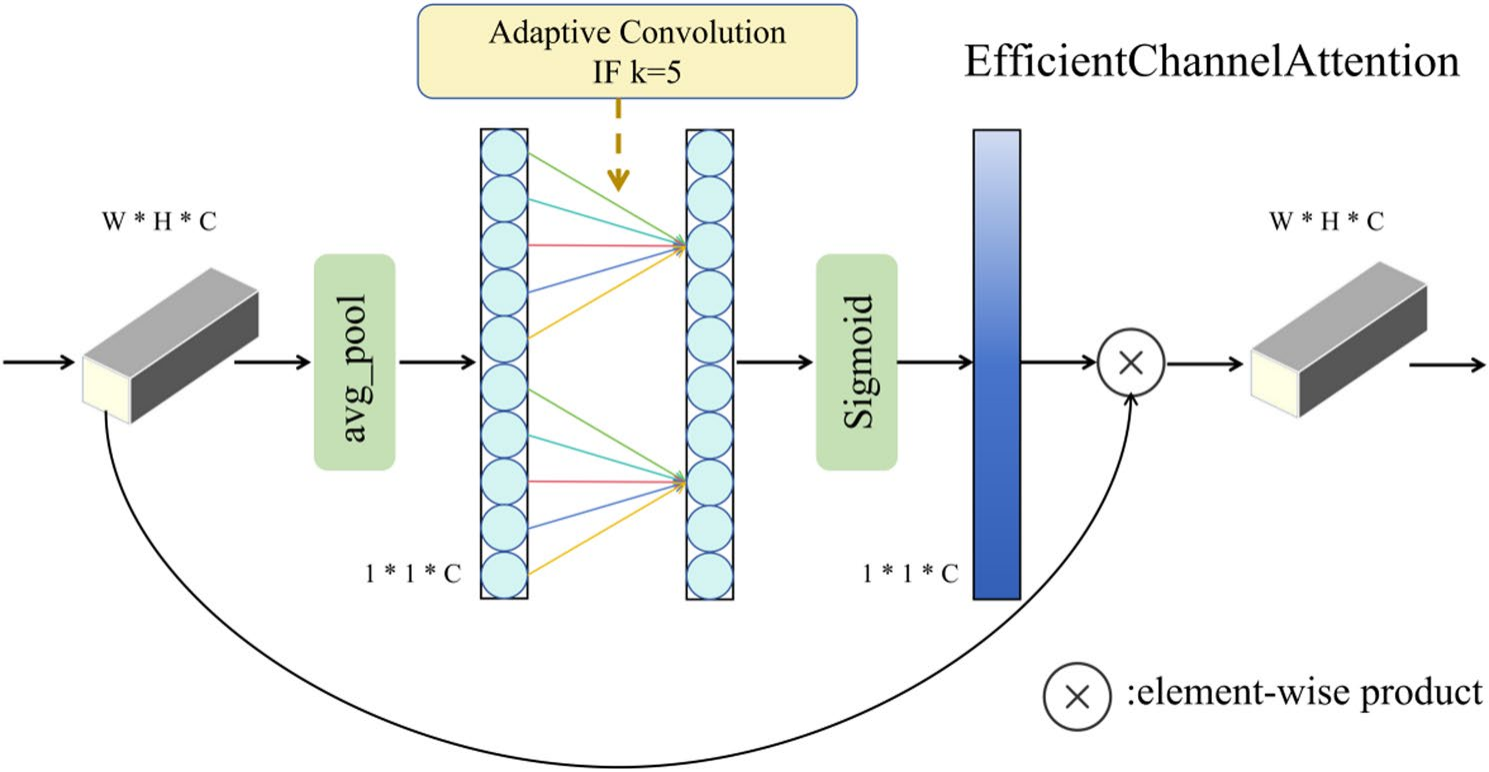
Structural diagram of the ECA attention mechanism

As illustrated in Fig. 9, the core idea of the ECA mechanism is to eliminate the dimensionality reduction caused by fully connected layers in conventional attention modules. Instead, it employs a one-dimensional convolution (1D Conv) to achieve efficient cross-channel interaction, capturing critical channel dependencies with minimal computational overhead. Specifically, the input feature map is first processed by global average pooling (GAP) to obtain a channel-wise statistical vector. The ECA module then applies a 1D convolution with a kernel size of kkk along the channel dimension to model local inter-channel interactions, as formulated in Eq. (10):

10 $$s = \sigma \left( {Conv1D_{k} (z)} \right)$$

where $\sigma(\cdot {\mathrm{)}}$ denotes the Sigmoid activation function. The resulting channel weights s are then multiplied element-wise with the original input feature map to obtain the recalibrated output, as defined in Eq. (11):

11 $$Y = X~ \odot ~s$$

The weighted feature map emphasizes lesion textures and pest details while suppressing redundant background information. It is noteworthy that the convolution kernel size $\mathrm{k}$ in the ECA module is adaptively determined according to the channel dimension, as formulated in Eq. (12):

12 $$k = \psi (C) = \left| {\frac{{\log _{2} C}}{\gamma } + b} \right|_{{odd}}$$

where γ,b is a hyperparameter and the operator ${\left|\right|}_{\mathrm{odd}}$ denotes taking the nearest odd integer to ensure kernel symmetry.

This design allows the ECA module to remain lightweight while retaining strong adaptability, automatically adjusting the interaction range according to the number of feature channels.

Embedding the ECA module into the YOLOv12 architecture significantly enhances the model’s ability to distinguish lesions and pests under complex field conditions. As a lightweight channel attention mechanism, ECA highlights discriminative channels associated with lesions and pest features while suppressing redundant background responses, improving the overall effectiveness of feature representation without noticeable computational overhead. When integrated with the LFEDH detection head, this mechanism proves particularly effective for small-object detection and bounda.

### LAMP pruning and fine-tuning

In deep detection models, increasing network depth and width can significantly improve detection performance but also leads to large parameter sizes and high computational costs, limiting deployment on edge devices such as agricultural IoT terminals and unmanned aerial platforms. To address this challenge, a Layer-wise Adaptive Magnitude-based Pruning (LAMP) strategy is introduced during model compression, combined with a fine-tuning stage to achieve lightweight inference while preserving detection accuracy.

The core principle of LAMP pruning is to perform channel-level sparsification based on the importance of weight magnitudes. Specifically, convolutional layer weights are globally ranked according to their norms, and channels are adaptively retained layer by layer based on a predefined sparsity ratio, removing redundant or low-contribution parameters. Unlike conventional global pruning methods, LAMP employs layer-wise normalization to dynamically balance the pruning ratios across different layers, preventing excessive reduction in shallow feature maps that could degrade small-object detection performance. The channel selection process can be formally expressed as Eq. (13):

13 $$s_{{i,\delta }} = \frac{{\parallel W_{{i,\delta }} \parallel _{2} }}{{\mathop \sum \nolimits_{{j = 1}}^{N} \parallel W_{{j,\delta }} \parallel _{2} }}$$

where ${\mathrm{W}}_{{\mathrm{l}}_{\mathrm{i}}}$ denotes the convolution kernel weights of the ${\text{l - th}}$ layer,${\mathrm{C}}_{\mathrm{l}}$ represents the total number of channels in that layer, and ${\mathrm{S}}_{\mathrm{l,i}}$ denotes the importance score of the corresponding channel. Channels are then pruned according to their scores and the predefined sparsity threshold, resulting in a sparsified network structure.

Pruning inevitably alters the parameter distribution and may cause partial feature loss. To mitigate this issue, a fine-tuning stage is introduced after the LAMP pruning process. The sparsified model is retrained on the original maize pest and disease dataset to restore potentially degraded feature representations. The fine-tuning procedure adopts the same hyperparameter configuration as the main model but employs a lower learning rate to prevent excessive perturbation of the converged weights.

Through the combination of LAMP pruning and fine-tuning, the proposed model achieves substantial reductions in parameter count and GFLOPs while maintaining high detection accuracy. The inference speed (FPS) is also significantly improved. This design is particularly suitable for resource-constrained agricultural scenarios, enabling efficient deployment on edge devices such as UAVs and field monitoring nodes, and providing a practical solution for real-time maize pest and disease detection.

## Hyperparameter settings and evaluation metrics

### Hyperparameter settings

The proposed DELP-YOLOv12 model was implemented based on the Ultralytics YOLO framework.And the main hyperparameters used in the training process are summarized in Table 2.The number of training epochs was set to 150 to ensure full convergence on the maize pest and disease dataset.The batch size was configured as 32, balancing training stability and convergence speed within the limits of available GPU memory.The initial learning rate (lr0) was set to 0.01, and gradually decayed using a learning rate decay factor (lrf = 0.01) to prevent overfitting and enhance generalization in later training stages.The Stochastic Gradient Descent (SGD) optimizer was employed for its stability and reliable convergence characteristics.To avoid overtraining, an early stopping criterion with Patience = 30 was adopted, terminating training if no improvement was observed on the validation metrics within 30 consecutive epochs.Mosaic data augmentation was disabled during the final 20 epochs (Close mosaic = 20) to allow the model to adapt more effectively to the real distribution of field data and improve detection performance under actual conditions.

**Table 2:** Training hyperparameter settings

Hyperparameter	Value
Epochs	150
Batch size	32
lr0	0.01
Lr1	0.01
Optimizer	SGD
Patience	30
Close mosaic	20

Under this experimental configuration, the complete training process lasted for 150 epochs, with an average training time of approximately 27.0 s per epoch, resulting in a total training cost of about 1.12 GPU-hours.

The hyperparameter configurations used during the pruning and fine-tuning stages are listed in Table 3. During pruning, the Layer-adaptive Magnitude-based Pruning (LAMP) method was adopted with the global pruning strategy enabled (Global pruning = True). The target speed-up factor was set to 2.0 to balance inference efficiency and detection accuracy. To enhance the pruning effect, a regularization constraint (reg = 0.0005) was applied to the weights during sparse training, which was conducted for 500 epochs to improve weight sparsity and ensure stable convergence.

In the fine-tuning phase, a smaller initial learning rate (lr0 = 0.001) and batch size (16) were employed to recover the model’s performance after pruning. The model was fine-tuned for 300 epochs under the same training configuration as the main model, and an early stopping criterion (Patience = 15) was applied to prevent overfitting and restore detection precision effectively.

**Table 3:** Hyperparameters for pruning and fine-tuning

Stage	Hyperparameter	Value/Setting
Pruning	Pruning method	LAMP
	Global pruning	True
	Target speed-up factor	2.0 (expected inference acceleration)
	Regularization (reg)	0.0005
	Sparse training	True
	Sparse-learning epochs	500
Fine-tuning	Epochs	300
	Initial learning rate	0.001
	Optimizer	SGD
	Batch size	16
	Image size	640 × 640
	Early stopping patience	15
	Workers	4

### Evaluation metrics

To comprehensively evaluate the detection performance of the proposed model, several quantitative metrics were employed, including Precision (P), Recall (R), and mean Average Precision (mAP).In addition, model complexity and efficiency were assessed using Parameters, Model Size, GFLOPs, and Frames Per Second (FPS) as complementary indicators.A summary of the evaluation metrics is presented in Table 4.

**Table 4:** Evaluation metrics

Metric	Symbol	Definition
Precision	P	$\mathrm{P=}\frac{\mathrm{TP}}{\mathrm{TP+FP}}$
Recall	R	$\mathrm{R=}\frac{\mathrm{TP}}{\mathrm{TP+FN}}$
Mean average precision	mAP	$\mathrm{mAP=}\frac{\mathrm{1}}{\mathrm{N}}\sum_{\mathrm{i=1}}^{\mathrm{N}}\mathrm{A}{\mathrm{P}}_{\mathrm{i}}$
Parameters	Params(M)	—
Model size	Size(MB)	—
Floating-point operations	GFLOPs (G)	*FLOP*_S_ = 2 ✕ H_out_ ✕ W_out_ ✕ C_in_ ✕ K^2^ ✕ C_out_
Frames per second	FPS	$\mathrm{FPS=}\frac{{\mathrm{N}}_{\mathrm{fremes}}}{{\mathrm{T}}_{\mathrm{inferce}}}$

These metrics provide a comprehensive evaluation of the model from two perspectives: detection accuracy (P, R, and mAP) and model complexity (Parameters, Model Size, GFLOPs, and FPS).They jointly reflect the accuracy and robustness of the proposed model in maize pest and disease detection, while ensuring its lightweight structure and computational efficiency for practical deployment.

## Experimental results and analysis

### Ablation study

Table 5 summarizes the ablation results of the three proposed modules—C3k_DRN, LFEDH, and ECA—on the maize pest and disease detection dataset. Rather than merely reflecting numerical performance changes, these results provide insights into how different feature enhancement mechanisms affect detection behavior under complex field conditions.

When C3k_DRN is introduced independently, noticeable improvements are observed in Precision, Recall, and mAP50, indicating that structural re-parameterization enhances the backbone’s ability to capture fine-grained spatial patterns and texture variations of small pests and irregular lesions. This improvement mainly reduces missed detections, which explains the concurrent increase in recall-related metrics. In contrast, incorporating LFEDH alone leads to higher Precision but a slight reduction in Recall, suggesting that this detection head primarily refines local feature representation and bounding box regression, while being relatively conservative in activating uncertain targets. The ECA module, when applied independently, yields a more evident improvement in mAP50–95 than in mAP50, confirming that channel-wise attention effectively suppresses background interference and benefits precise localization under stricter IoU thresholds.

Further analysis of module combinations reveals complementary effects among the proposed components. The joint use of C3k_DRN and LFEDH significantly increases Recall, demonstrating that enhanced feature representation combined with refined local detection facilitates more robust target activation. Meanwhile, combining C3k_DRN with ECA achieves strong mAP50 performance, but with a slight drop in Precision, which can be attributed to increased sensitivity to complex background regions when feature expressiveness is amplified without sufficient localization refinement. Notably, when all three modules are integrated, the model achieves the best overall performance across all metrics, including Precision, mAP50, and mAP50–95. This result indicates that C3k_DRN, LFEDH, and ECA operate in a complementary manner, jointly enhancing feature modeling, local boundary regression, and channel selectivity. Their synergistic integration effectively balances detection sensitivity and localization accuracy, resulting in improved robustness under complex maize field environments.

**Table 5:** Ablation study results of the proposed modules on the maize pest and disease detection dataset

C3k_DRN	LFEDH	ECA	*P*	*R*	mAP50	mAP50-95
			89.90%	83%	90.10%	63.90%
√			92.50%	87.20%	92.30%	68.10%
	√		92.90%	82.90%	91.60%	66.10%
		√	92.50%	83.70%	91.50%	65.90%
√	√		92.20%	88.80%	93%	67%
√		√	89.80%	87.40%	92.80%	67.20%
	√	√	90.70%	87.20%	92.10%	67.40%
√	√	√	95.50%	88.50%	94.40%	70.50%

### Comparative experiments

**Fig. 10:**
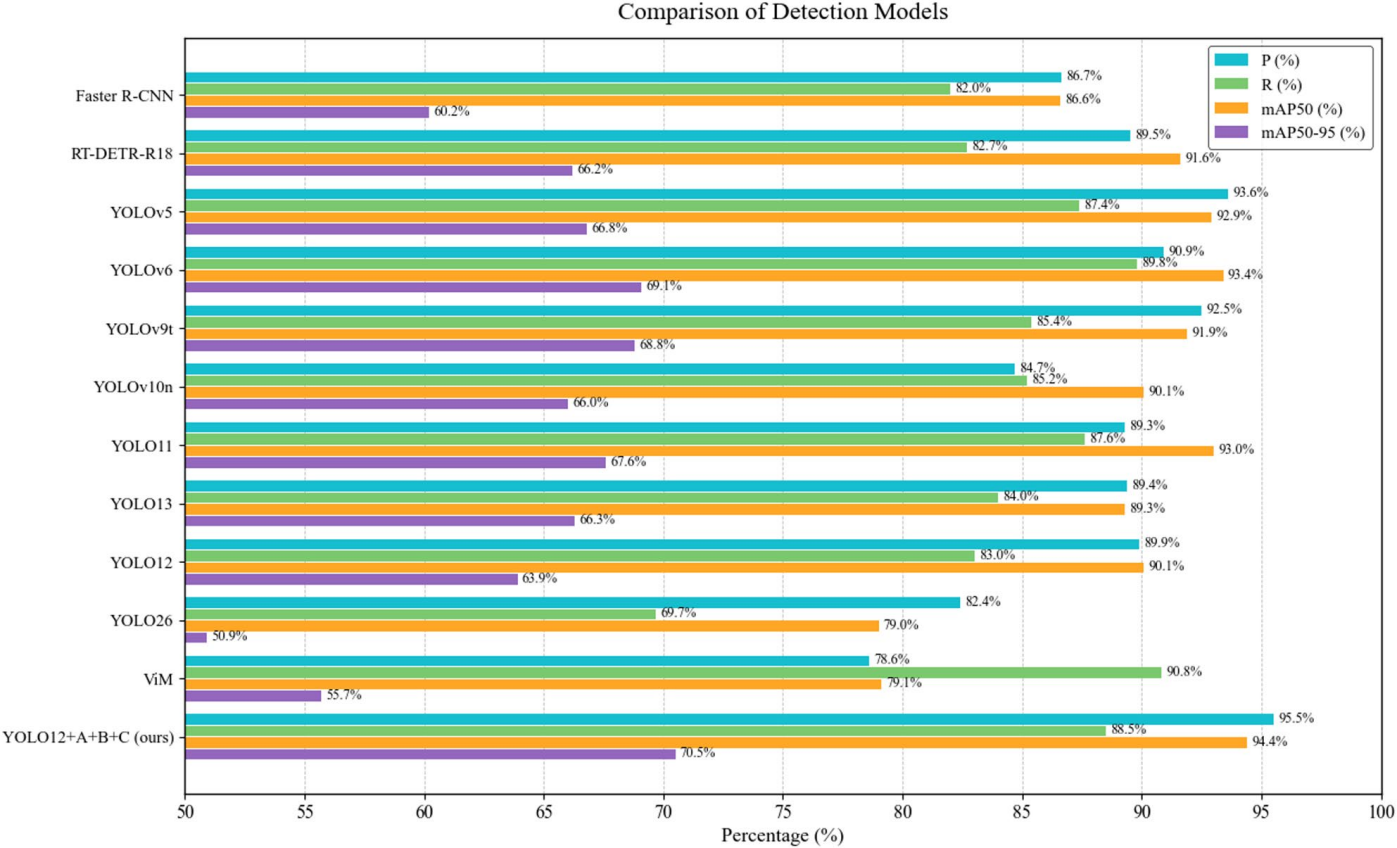
Comparative performance results of different detection models on the maize pest and disease dataset

To comprehensively validate the effectiveness of the proposed method, comparative experiments were conducted against representative object detection paradigms, including the two-stage detector Faster R-CNN, Transformer-based detectors (RT-DETR-R18 and ViM), and a series of YOLO-based models (YOLOv5, YOLOv6, YOLOv9t, YOLOv10n, YOLOv11, YOLOv13, YOLOv12, YOLO26, and the improved YOLOv12 variant). The evaluation metrics include Precision (P), Recall (R), mAP50, and mAP50–95, and the comparative results are illustrated in Fig. 10.

From the overall comparison, clear performance differences can be observed among detectors with different architectural paradigms. The two-stage detector Faster R-CNN shows limited adaptability to complex maize field environments, particularly under stricter localization criteria, as reflected by its relatively low mAP50–95 (60.20%). This behavior suggests that region proposal–based frameworks struggle to precisely localize small pests and irregular lesion boundaries in cluttered backgrounds. Transformer-based detectors, such as RT-DETR-R18 and ViM, benefit from global context modeling and achieve competitive detection performance at moderate IoU thresholds, with mAP50 exceeding 90% in some cases; however, their lower mAP50–95 values (e.g., 66.20% for RT-DETR-R18 and 55.70% for ViM) indicate that global attention alone is insufficient for capturing fine-grained local variations required for accurate pest and disease detection.

Within the YOLO family, most models demonstrate a more balanced trade-off between detection sensitivity and localization accuracy. Several established YOLO variants achieve stable localization performance, with mAP50–95 generally distributed in the range of approximately 66–69%, reflecting reliable detection behavior under typical field conditions. The baseline YOLOv12 emphasizes inference efficiency and model compactness for real-time deployment on edge devices [25], and while this design enables fast detection, its mAP50–95 (63.90%) remains lower than that of stronger YOLO baselines, indicating room for improving localization accuracy without sacrificing speed.

In contrast, the proposed DELP-YOLOv12, which integrates C3k_DRN, ECA channel attention, and the LFEDH detection head, demonstrates consistently improved detection robustness under complex field scenarios. The improved model achieves a notable increase in localization accuracy, with mAP50–95 reaching 70.50%, representing a 6.6% point improvement over the baseline YOLOv12. These gains are achieved without relying on increased model capacity, but rather through targeted feature enhancement strategies that improve feature representation, suppress background interference, and refine boundary regression. As a result, the improved model maintains higher stability when confronted with blurred lesion boundaries, small-scale insect targets, and cluttered backgrounds, achieving a more favorable balance among detection accuracy, localization precision, and inference efficiency. These characteristics make DELP-YOLOv12 particularly suitable for practical maize pest and disease monitoring applications where both accuracy and real-time performance are required.

### Model pruning and fine-tuning

To further enhance inference efficiency and deployment adaptability, structural pruning and fine-tuning experiments were conducted on the improved YOLOv12 model.

By evaluating the importance of convolutional channels, redundant parameters were identified and selectively removed, resulting in the lightweight DEL-YOLOv12 and DELP-YOLOv12 models.

**Fig. 11:**
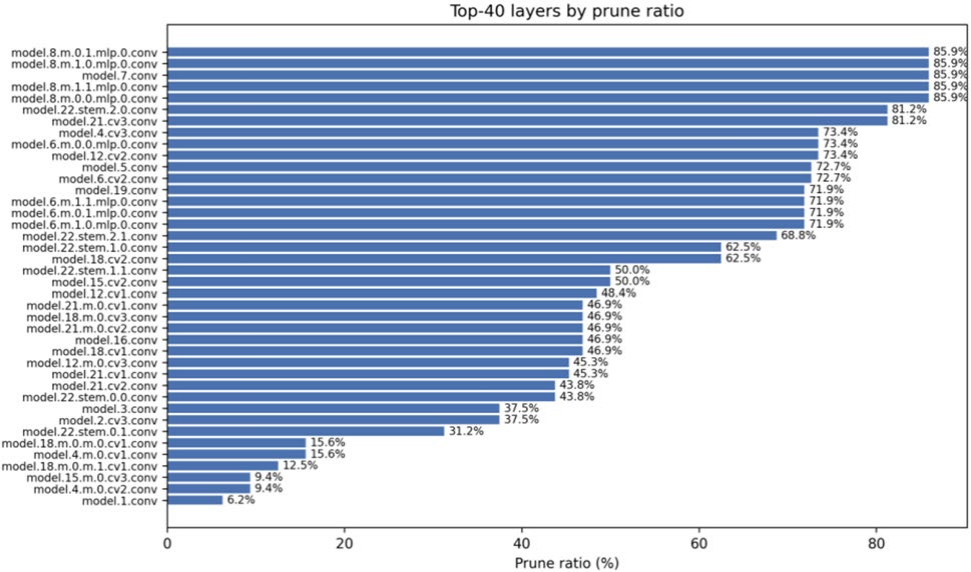
Top-40 ranking of layer-wise pruning rates in the improved YOLOv12 model

As illustrated in Fig. 11, the degree of redundancy varied significantly across different convolutional layers, with pruning rates reaching up to 85.9% in some layers, while remaining as low as 6.2% in several shallow layers. Overall, the latter part of the network and the MLP module exhibited higher channel redundancy.

**Fig. 12:**
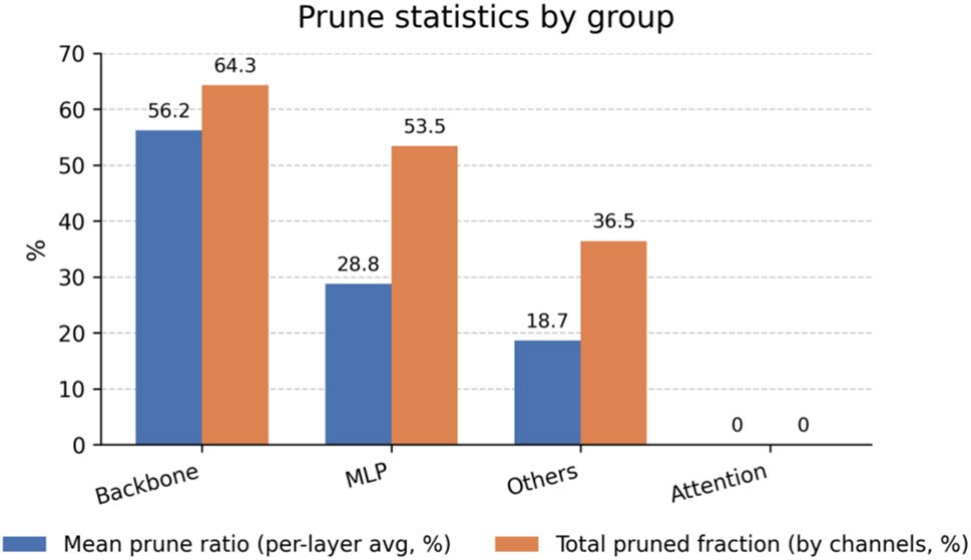
Average pruning rates and channel reduction ratios across different network components

Further statistical analysis (Fig. 12) indicates that the Backbone achieved an average pruning rate of 56.2%, contributing to an overall channel reduction of 64.3%, whereas the MLP module showed a relatively modest average pruning rate of 28.8%, suggesting that the backbone’s redundancy contributed more substantially to model compression.

**Fig. 13:**
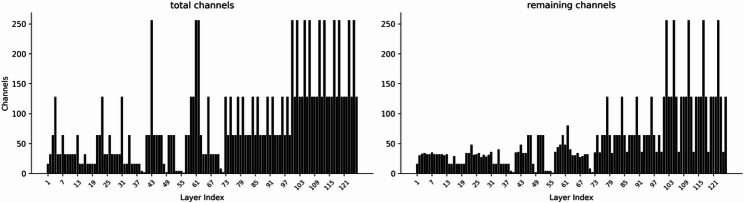
Changes in channel distribution before and after model pruning

From the channel distribution shown in Fig. 13, the number of channels varied considerably across layers before pruning, whereas redundant channels were effectively removed after pruning, resulting in a notable overall reduction in channel count and a more compact network structure.This strategy significantly reduces model complexity while maintaining the backbone’s feature extraction capability.

**Fig. 14:**
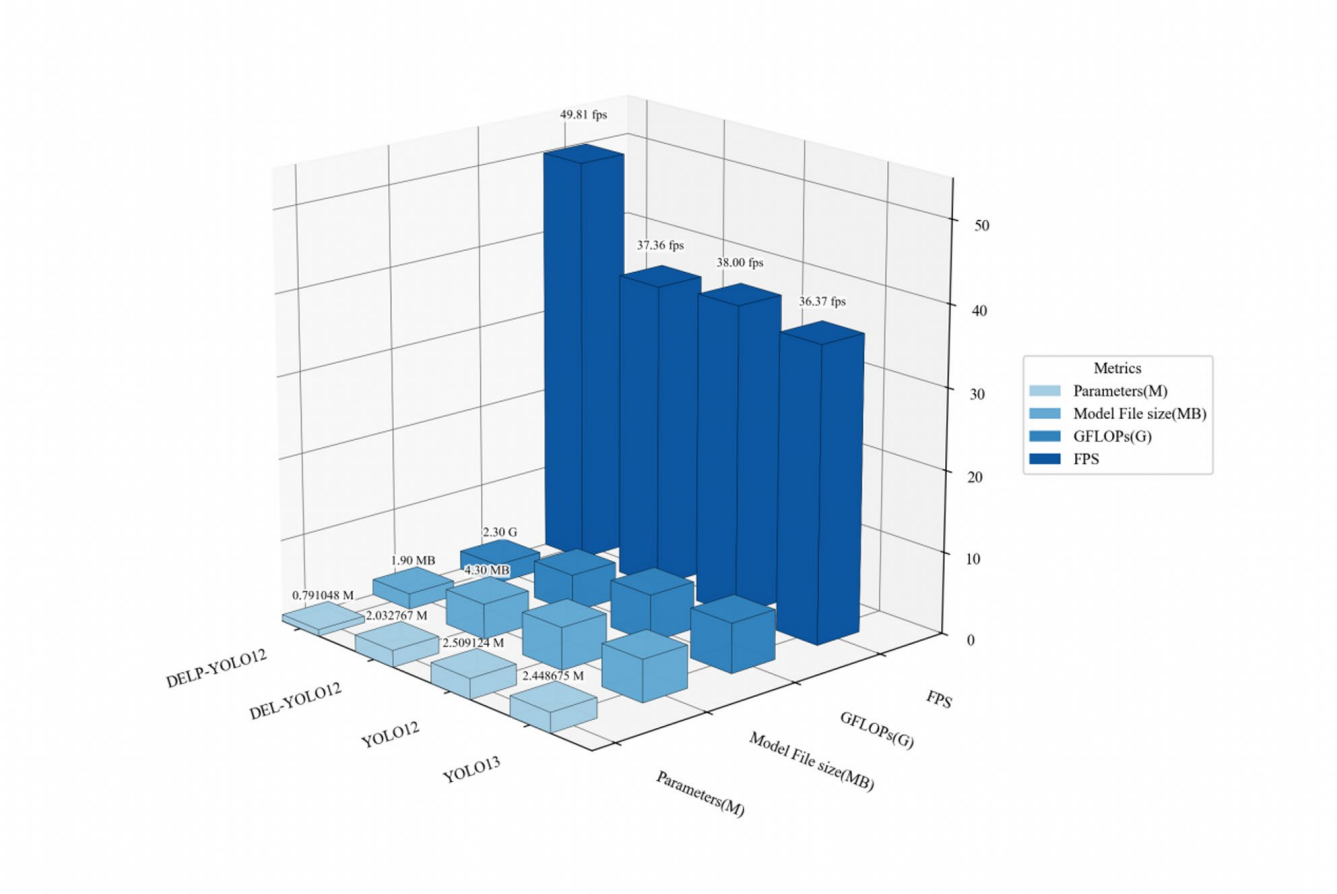
Comparison of parameters, computational cost, and inference speed among different models

As shown in Fig. 14, the baseline YOLOv12 model contains 2.50 M parameters and achieves an inference speed of 38 FPS.After pruning, the DEL-YOLOv12 model reduces the parameter count to 0.79 M, while the inference speed increases to 49.81 FPS, demonstrating a clear improvement over the original model.With the integration of fine-tuning, the DELP-YOLOv12 model maintains comparable detection accuracy while achieving an optimal balance between computational efficiency and memory cost, confirming the effectiveness of the pruning-plus-fine-tuning strategy.In contrast, YOLOv13 and other baseline models exhibit similar parameter sizes and computational loads to YOLOv12, but their inference speeds are noticeably lower, indicating that the proposed lightweight framework offers a distinct advantage in both speed and efficiency.

Considering the application context of maize pest and disease detection, the lightweight model obtained through pruning and fine-tuning achieves faster inference speed and lower memory consumption on resource-constrained field devices while maintaining high detection accuracy. Specifically, DELP-YOLOv12 achieved a precision of 94.1%, a recall of 89.6%, and an mAP50 of 94.2%. Compared with the pre-pruned DEL-YOLO model (precision = 95.50%, recall = 88.50%, and mAP50 = 94.40%), the proposed model shows only minor performance variation, with a 1.40%-point decrease in precision, a 1.10%-point increase in recall, and a negligible 0.20%-point reduction in mAP50. Meanwhile, the parameter count and computational cost were reduced by approximately 68% and 60%, respectively, significantly improving deployment efficiency. This favorable trade-off between accuracy and efficiency is of great significance for real-time monitoring and early warning of pest and disease outbreaks, providing effective technical support for green prevention strategies and intelligent agricultural management.

### Visualization analysis

**Fig. 15:**
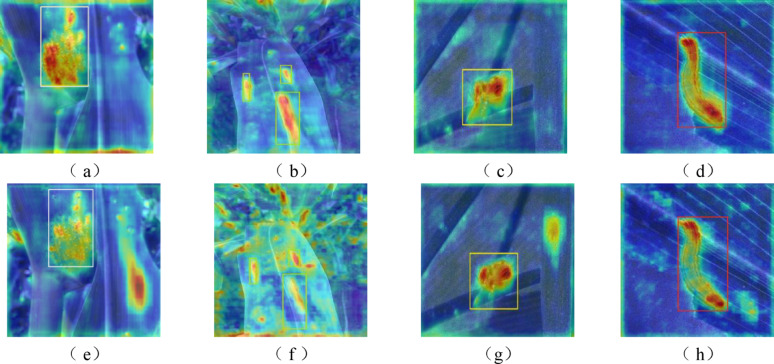
Grad-CAM visualization comparison. **a–d** Heatmaps of the improved DELP-YOLOv12; **e–h** Heatmaps of the baseline YOLOv12

To further verify the effectiveness of the proposed model in maize pest and disease detection, Grad-CAM (Gradient-weighted Class Activation Mapping) was employed to visualize the model’s attention regions.The comparative results are shown in Fig. 15, where (a)–(d) represent the heatmaps of the improved DELP-YOLOv12, and (e)–(h) correspond to those of the baseline YOLOv12.

The heatmaps were generated by weighting the class gradients of the final backbone convolutional feature maps, followed by upsampling and overlaying them onto the original images (colormap: jet), with normalization applied for comparability.

In the visualization, red denotes regions with high attention (strong class evidence), whereas blue indicates low attention. White and yellow boxes mark manually annotated lesions or pest regions (ground truth), while red boxes represent the model’s predicted bounding boxes.

The visual comparison clearly demonstrates that the improved model focuses more accurately on lesion and pest regions across most samples, exhibiting better alignment between high-attention areas and the annotated boxes than the baseline.Specifically, in samples with blurred boundaries or low color contrast between lesions and background (e.g., Figures a and c), the improved model successfully locates the lesion cores.In cases involving small-scale or irregularly shaped pest targets (e.g., Figures b and d), the model exhibits stronger responses to small objects while effectively suppressing background noise, thereby reducing both missed detections and false positives.In contrast, the baseline model (Figures e–h) often shows dispersed attention or strong responses to leaf veins and background textures, leading to attention regions deviating from the true lesion areas. This observation is consistent with its lower mAP50-95 performance.

The visualization analysis provides an intuitive and convincing interpretability complement to the quantitative results.The C3k_DRN module enhances multi-scale contextual representation, the ECA mechanism strengthens channel-wise attention, and the Detect_LFEDH module improves the detection head’s response to irregular lesions and small-scale targets.

Together, these components synergistically enhance the model’s discriminative capability under complex field conditions in maize pest and disease detection.

### System interface and application performance

To demonstrate the practical applicability of the proposed model in real agricultural production, an intelligent maize pest and disease detection system was developed based on the DELP-YOLOv12 model.

**Fig. 16:**
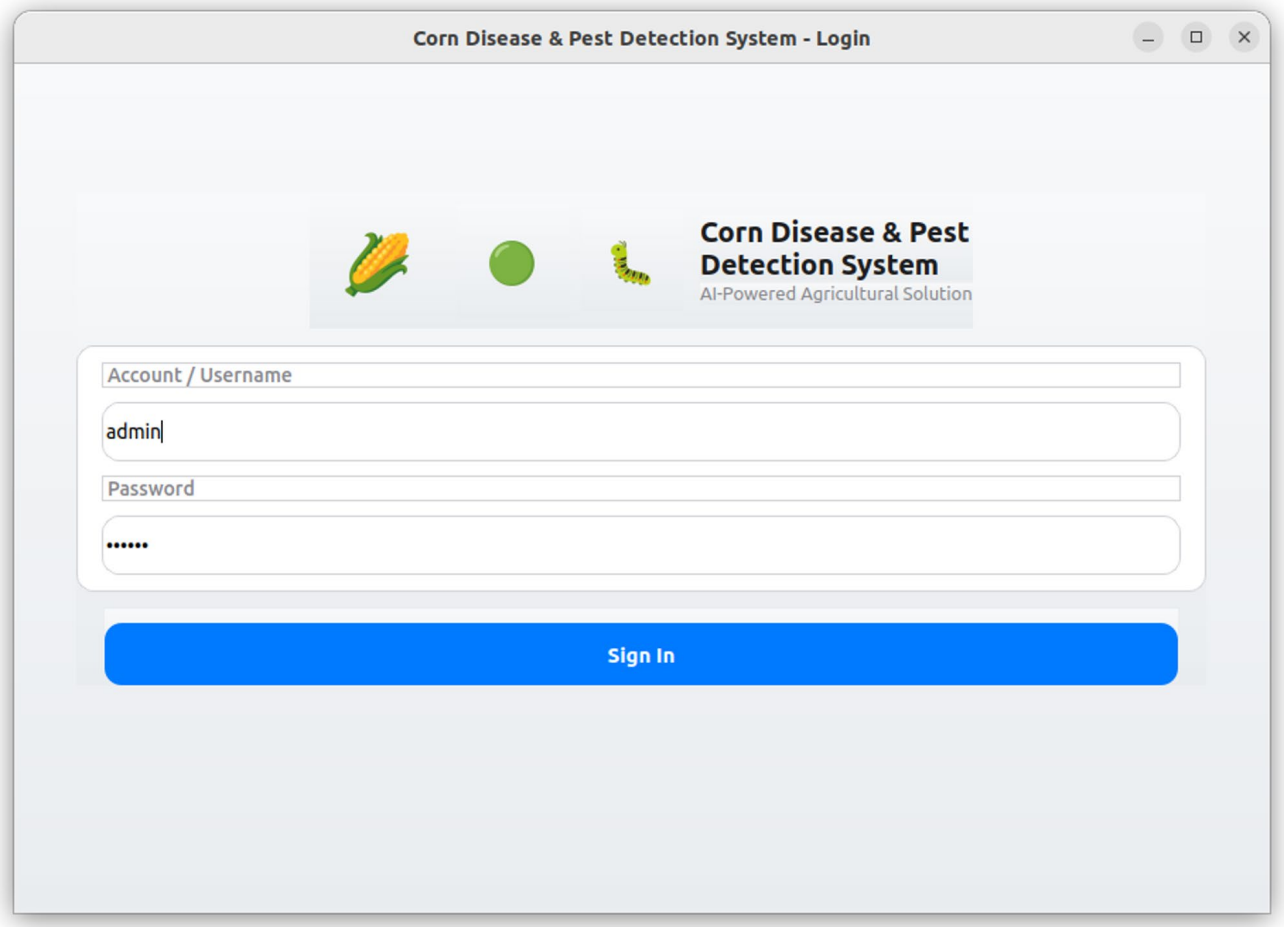
Login interface of the maize pest and disease detection system

**Fig. 17:**
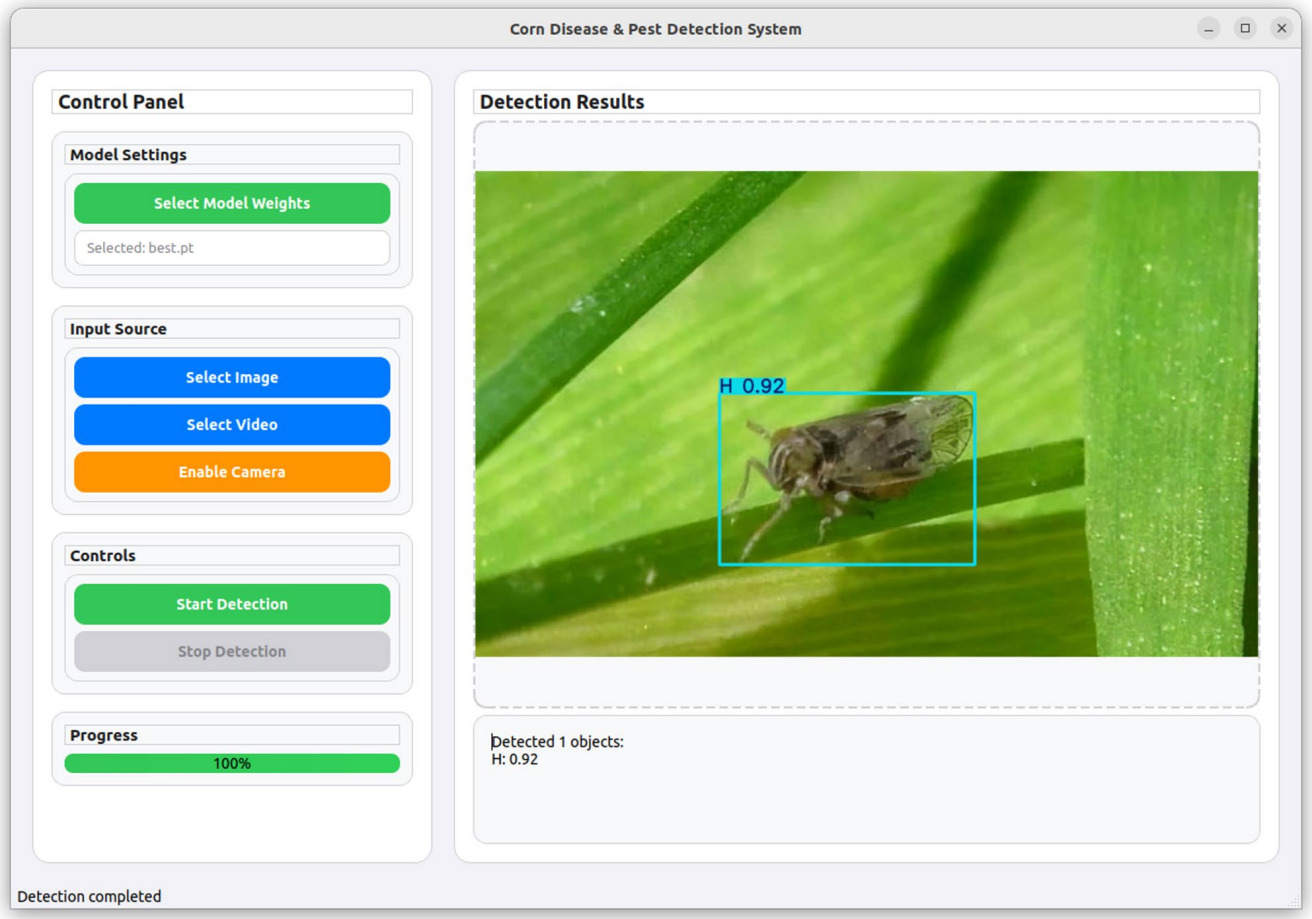
Main interface and detection result display of the maize pest and disease detection system

As shown in Fig. 16, the system provides a user login function to ensure data security and access control during actual deployment. Figure 17 presents the main detection interface, where the left panel serves as the functional control area, allowing users to select model weight files, import images or videos, or enable real-time camera detection.The right panel displays the detection results in real time, including bounding boxes and confidence scores of the detected lesions or pests.After detection, the system outputs the number and category of identified targets, enabling users to intuitively interpret the detection outcomes.

The system supports multiple input modes, including static images, video streams, and real-time camera feeds.Combined with the proposed lightweight strategy, it can operate efficiently on resource-constrained field-end devices, achieving real-time recognition and early warning of maize pests and diseases.This implementation provides a practical and efficient technical solution for intelligent agriculture, supporting sustainable and data-driven pest management in field environments.

### Limitations

This study has several limitations that should be acknowledged. The maize pest and disease dataset used in this work is mainly collected during the maturity stage, while samples from other growth stages are relatively limited. Such an imbalance across the crop growth cycle may affect the model’s performance when applied to early-stage symptoms or pest appearances that differ from those observed at maturity.Although the proposed detection framework is designed for real field environments and data augmentation strategies are applied during training, complex imaging conditions—including drastic illumination variation, motion blur, severe target occlusion, and cluttered backgrounds—may still lead to detection performance degradation. Under these conditions, detecting extremely small targets and densely distributed pest instances remains challenging. This issue can become more pronounced after model pruning when inference efficiency is prioritized, as fine-grained feature representation may be partially affected.

The deployment experiments in this study are conducted only on the NVIDIA Jetson Orin NX, which represents a mid-range edge computing platform. The performance of the proposed method on lower-power or heterogeneous edge devices has not been evaluated. In addition, potential biases may arise from class imbalance and annotation subjectivity within the dataset, which could influence the learned feature representations.

## Conclusion

This study proposes and implements a lightweight detection framework, termed DELP-YOLOv12, for maize pest and disease detection under complex field conditions, aiming to balance detection accuracy and deployment efficiency. Based on the YOLOv12 architecture, the framework integrates C3k_DRN structural re-parameterization, ECA channel attention, and the LFEDH detection head, together with LAMP-based pruning and fine-tuning, to achieve joint algorithmic and engineering optimization.

Extensive comparative and ablation experiments on the maize pest and disease dataset demonstrate that the proposed DEL-YOLOv12 achieves a Precision of 95.5%, a Recall of 88.5%, an mAP50 of 94.4%, and an mAP050-95 of 70.5%. After LAMP-based pruning and fine-tuning, the number of model(DELP-YOLOv12) parameters is reduced from approximately 2.03 M to 0.79 M, while the inference speed increases from 37.36 FPS to 49.81 FPS, with only a marginal reduction in detection accuracy where the precision and mAP50 decrease by 1.40 and 0.20% points, respectively, and the recall even increases by 1.10% points. In addition, Grad-CAM visualizations and engineering implementation results indicate that the proposed framework can effectively focus on pest and lesion regions and support near real-time inference on resource-constrained edge devices.

Despite these results, several limitations remain. The current dataset mainly covers specific maize growth stages and field environments, which may restrict model generalization, and detecting extremely small or heavily occluded targets remains challenging, especially after aggressive pruning. Future work will focus on expanding cross-domain datasets, improving small-object detection through enhanced multi-scale feature modeling, and exploring additional lightweight optimization strategies, such as quantization and knowledge distillation, accompanied by further field-level validation.

## Supplementary Information

Supplementary Material 1.

## Data Availability

The original contributions presented in the study are included in this published article and its supplementary materials. Further inquiries can be directed to the corresponding author.
