# Supplementary figures and images for: DELP-YOLOv12: a lightweight deployable model for maize pest and disease detection
Source: Plant Methods. 2026 Mar 31;22:59. doi: 10.1186/s13007-026-01527-4 (PMC13321971; doi:10.1186/s13007-026-01527-4)

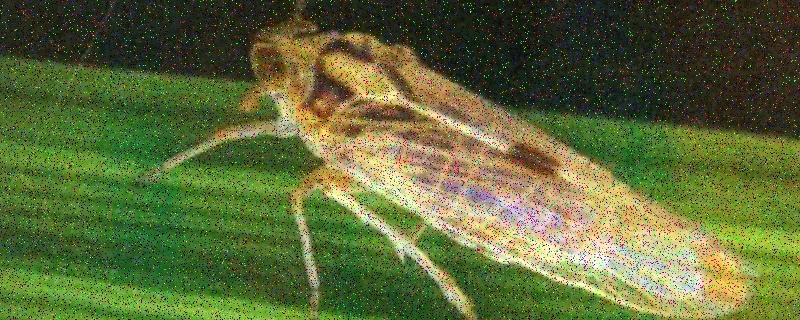

Supplement: Supplementary file 1 — Supplementary Material 1. [file 13007_2026_1527_MOESM1_ESM.zip › same_data/14173137114_noise.jpg]

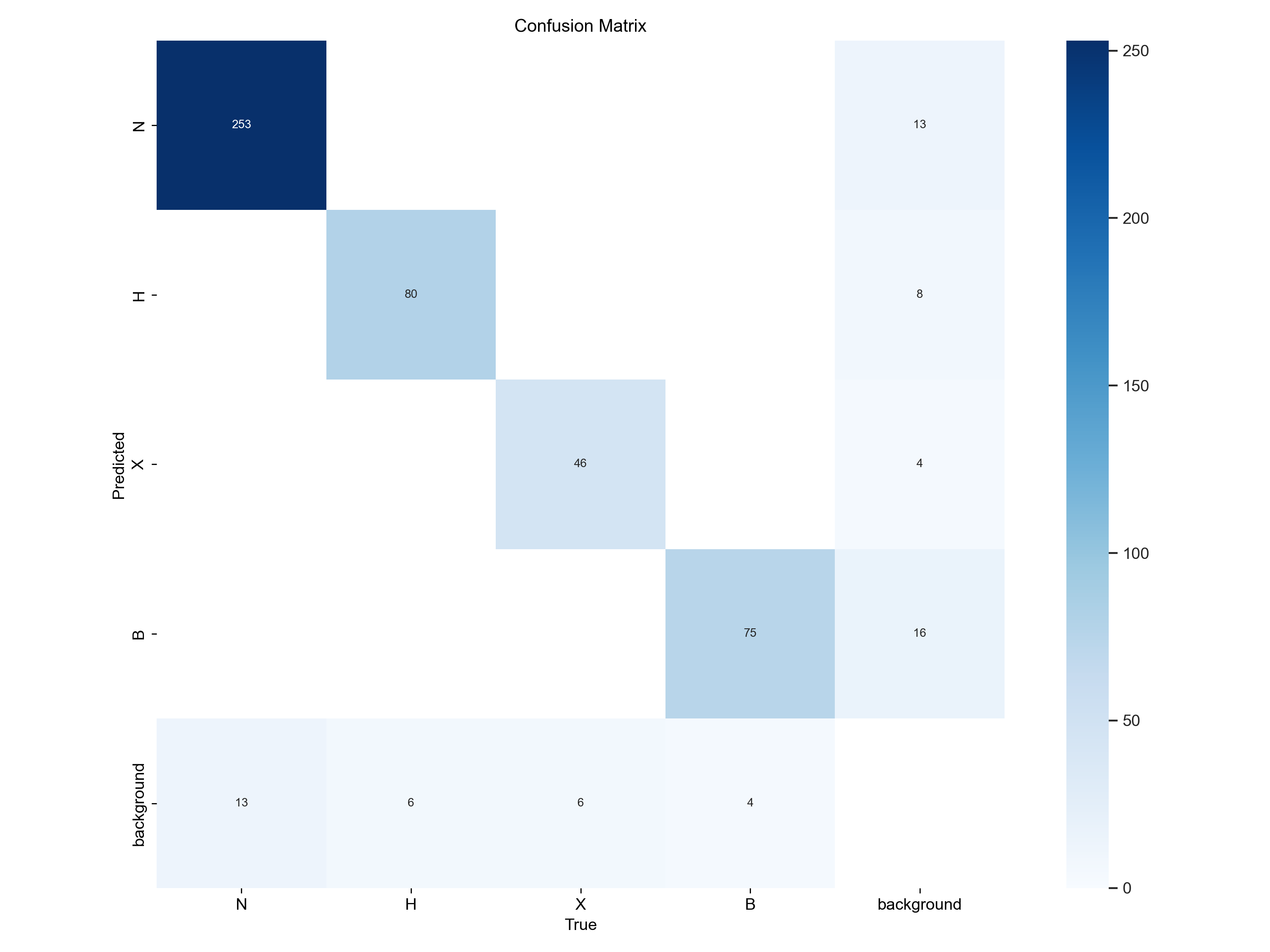

Supplement: Supplementary file 1 — Supplementary Material 1. [file 13007_2026_1527_MOESM1_ESM.zip › same_data/abc-finetune2.0/confusion_matrix.png]

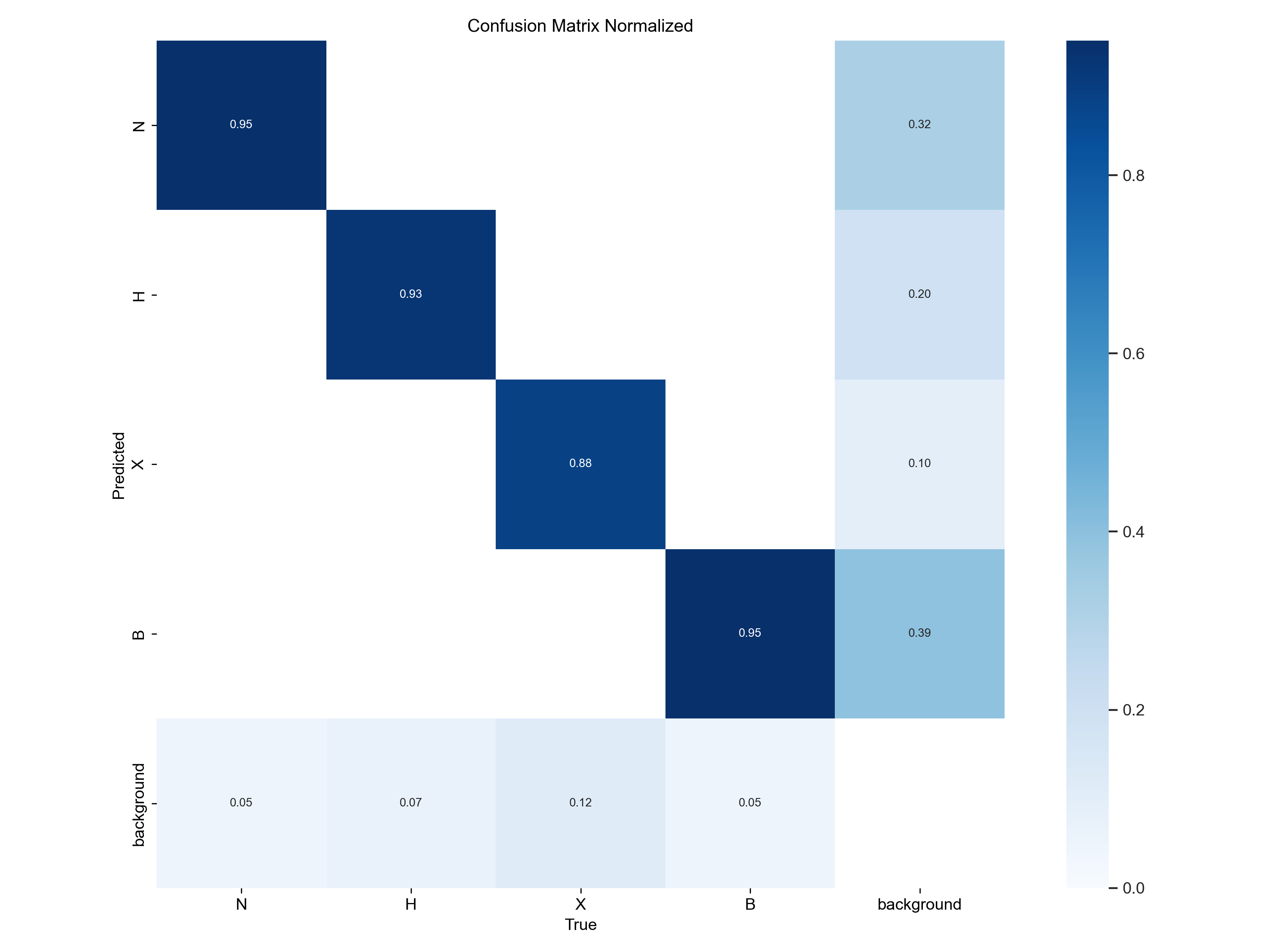

Supplement: Supplementary file 1 — Supplementary Material 1. [file 13007_2026_1527_MOESM1_ESM.zip › same_data/abc-finetune2.0/confusion_matrix_normalized.png]

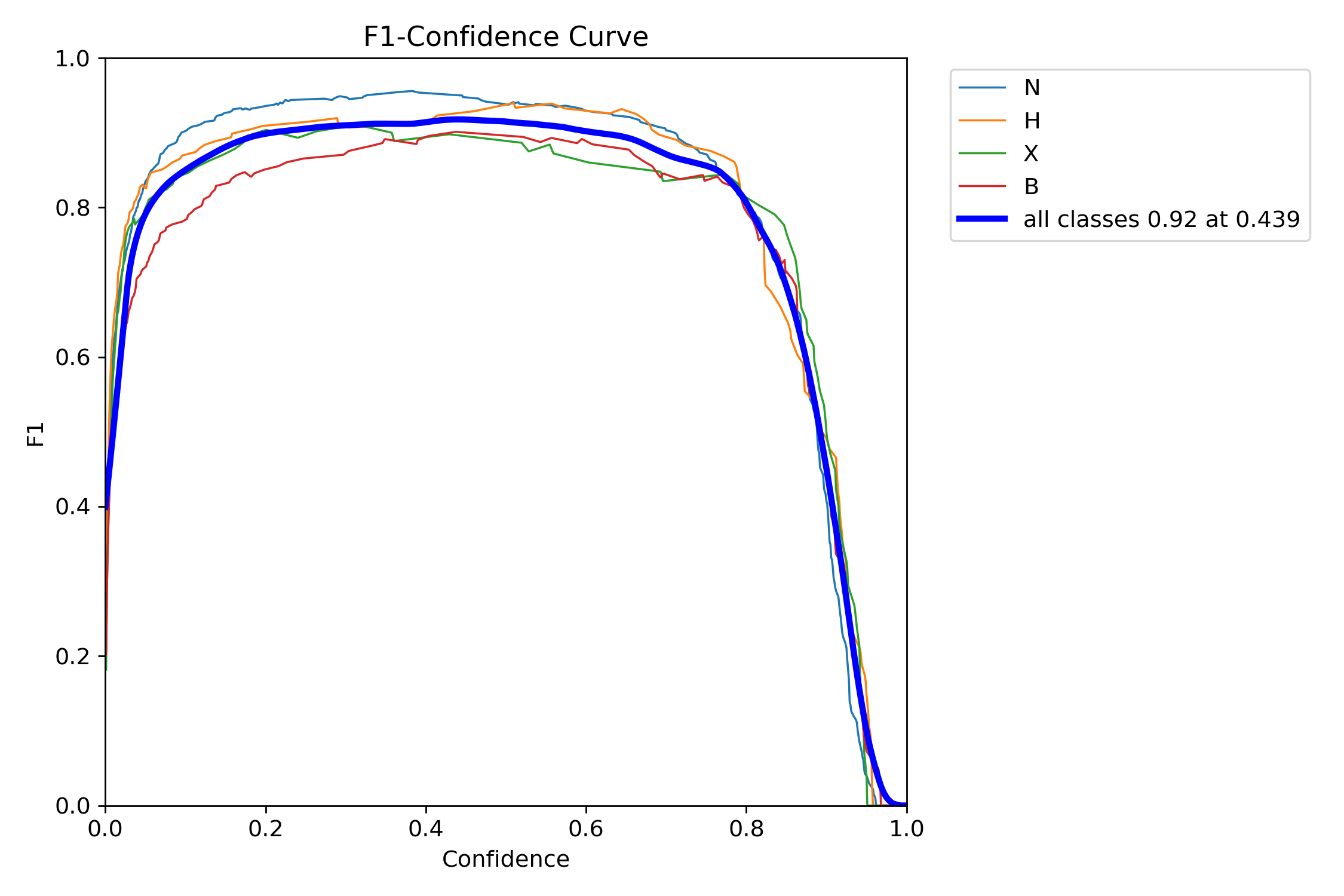

Supplement: Supplementary file 1 — Supplementary Material 1. [file 13007_2026_1527_MOESM1_ESM.zip › same_data/abc-finetune2.0/F1_curve.png]

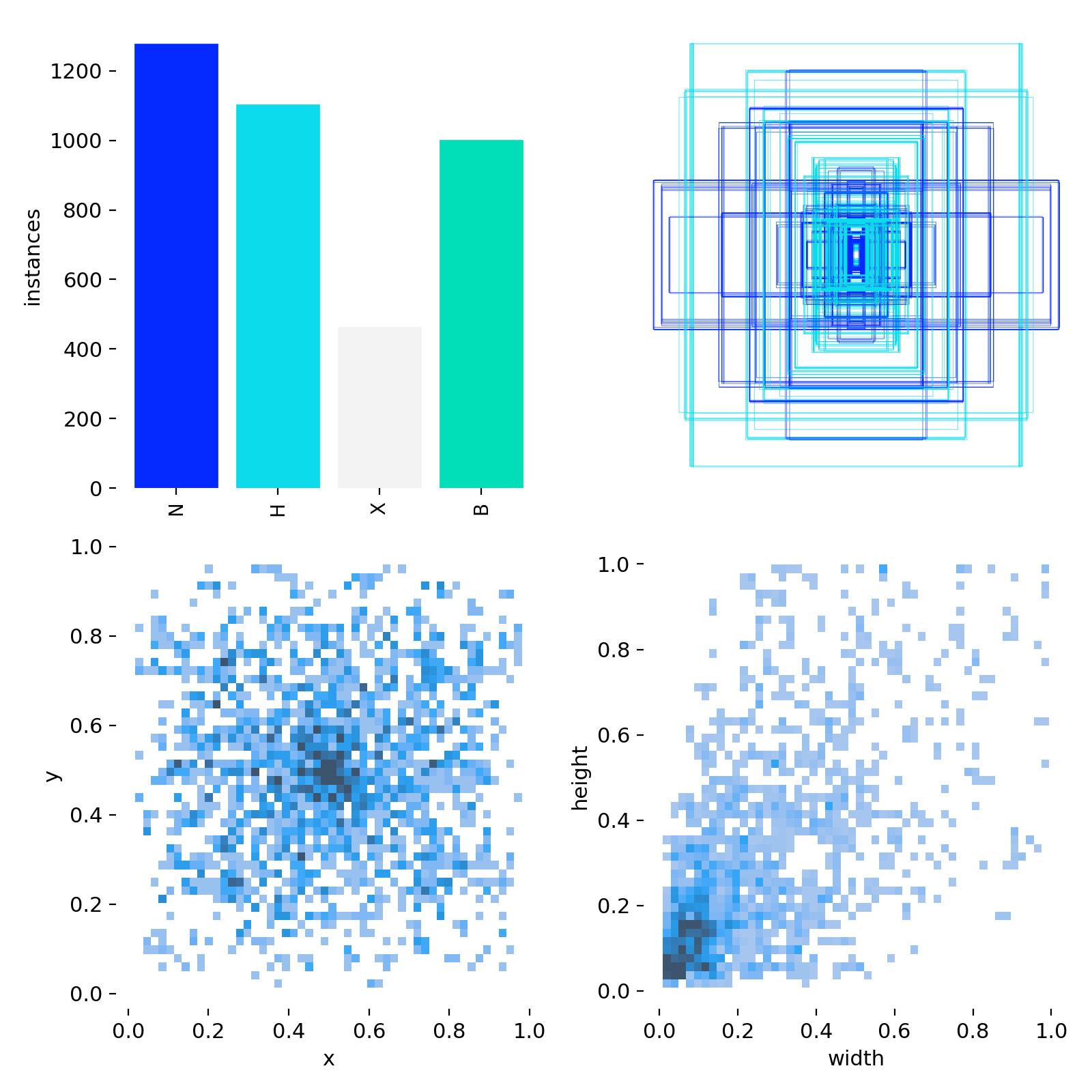

Supplement: Supplementary file 1 — Supplementary Material 1. [file 13007_2026_1527_MOESM1_ESM.zip › same_data/abc-finetune2.0/labels.jpg]

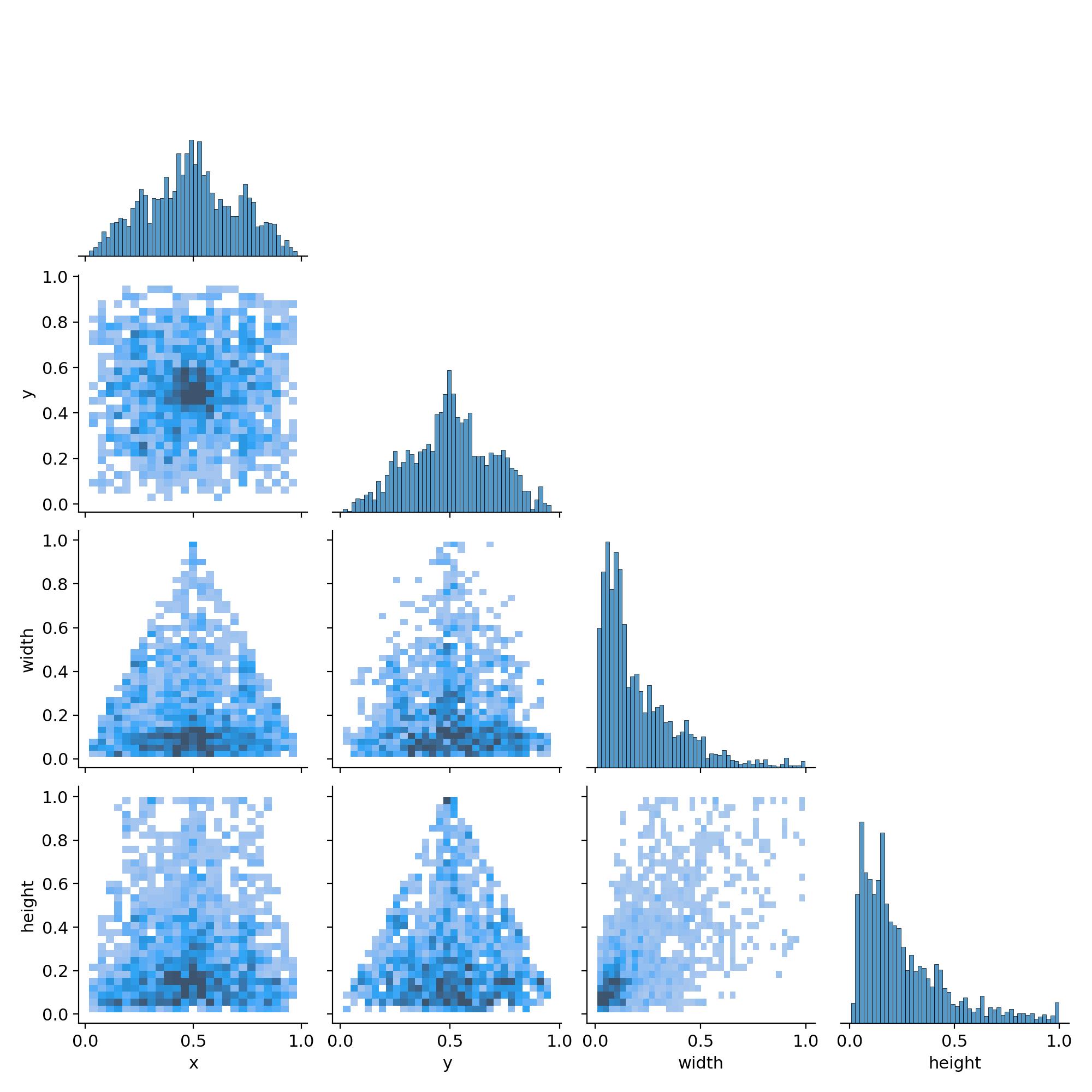

Supplement: Supplementary file 1 — Supplementary Material 1. [file 13007_2026_1527_MOESM1_ESM.zip › same_data/abc-finetune2.0/labels_correlogram.jpg]

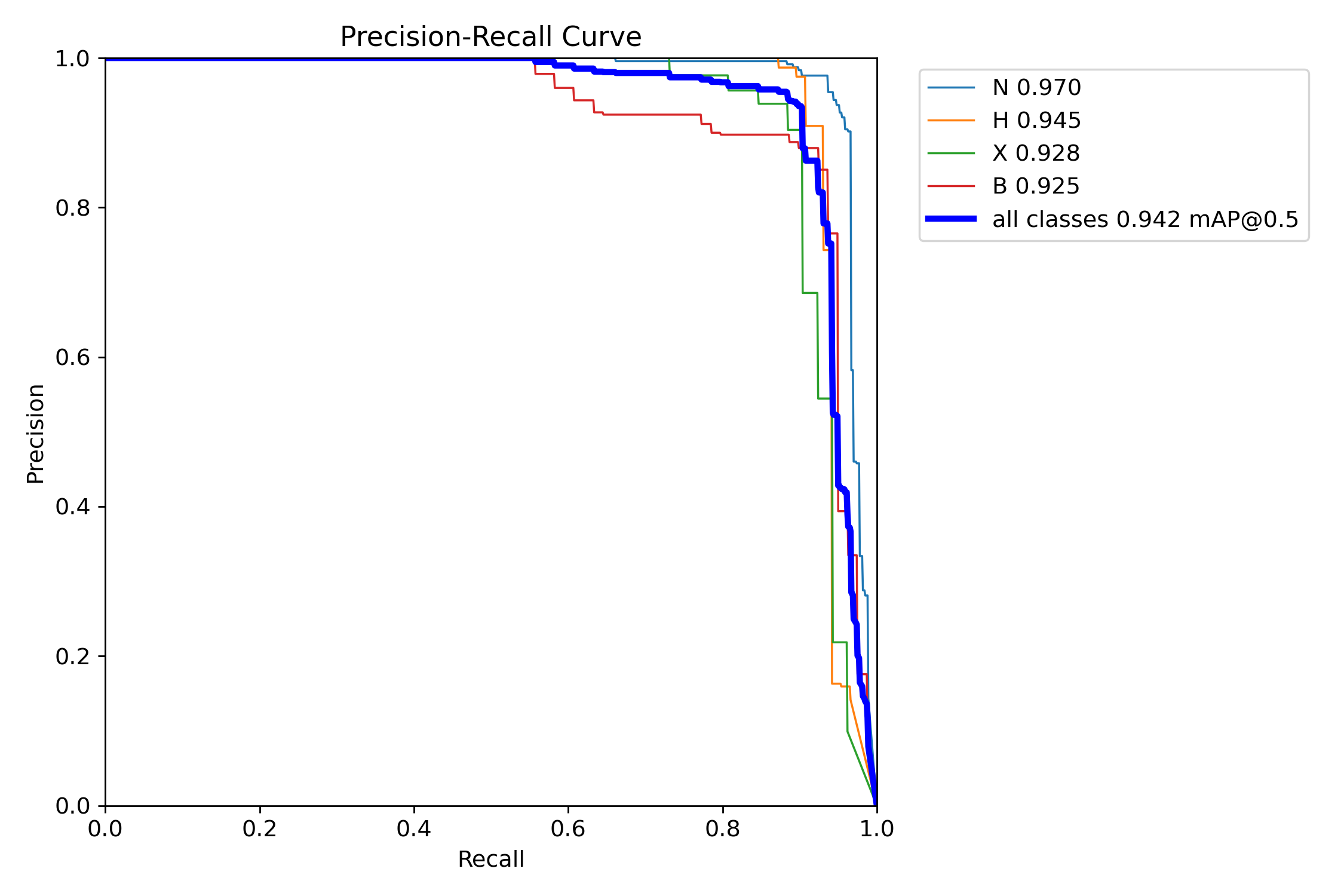

Supplement: Supplementary file 1 — Supplementary Material 1. [file 13007_2026_1527_MOESM1_ESM.zip › same_data/abc-finetune2.0/PR_curve.png]

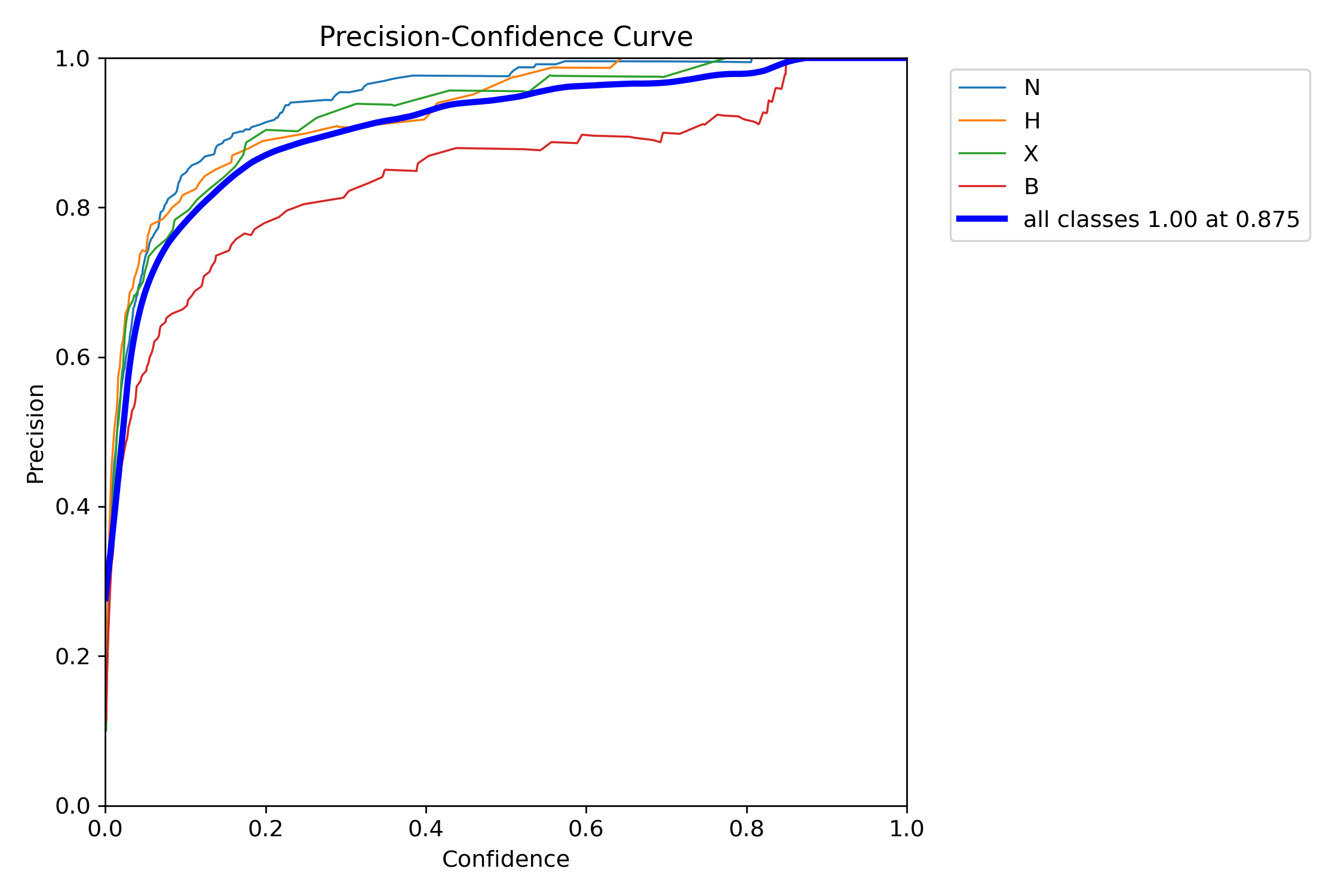

Supplement: Supplementary file 1 — Supplementary Material 1. [file 13007_2026_1527_MOESM1_ESM.zip › same_data/abc-finetune2.0/P_curve.png]

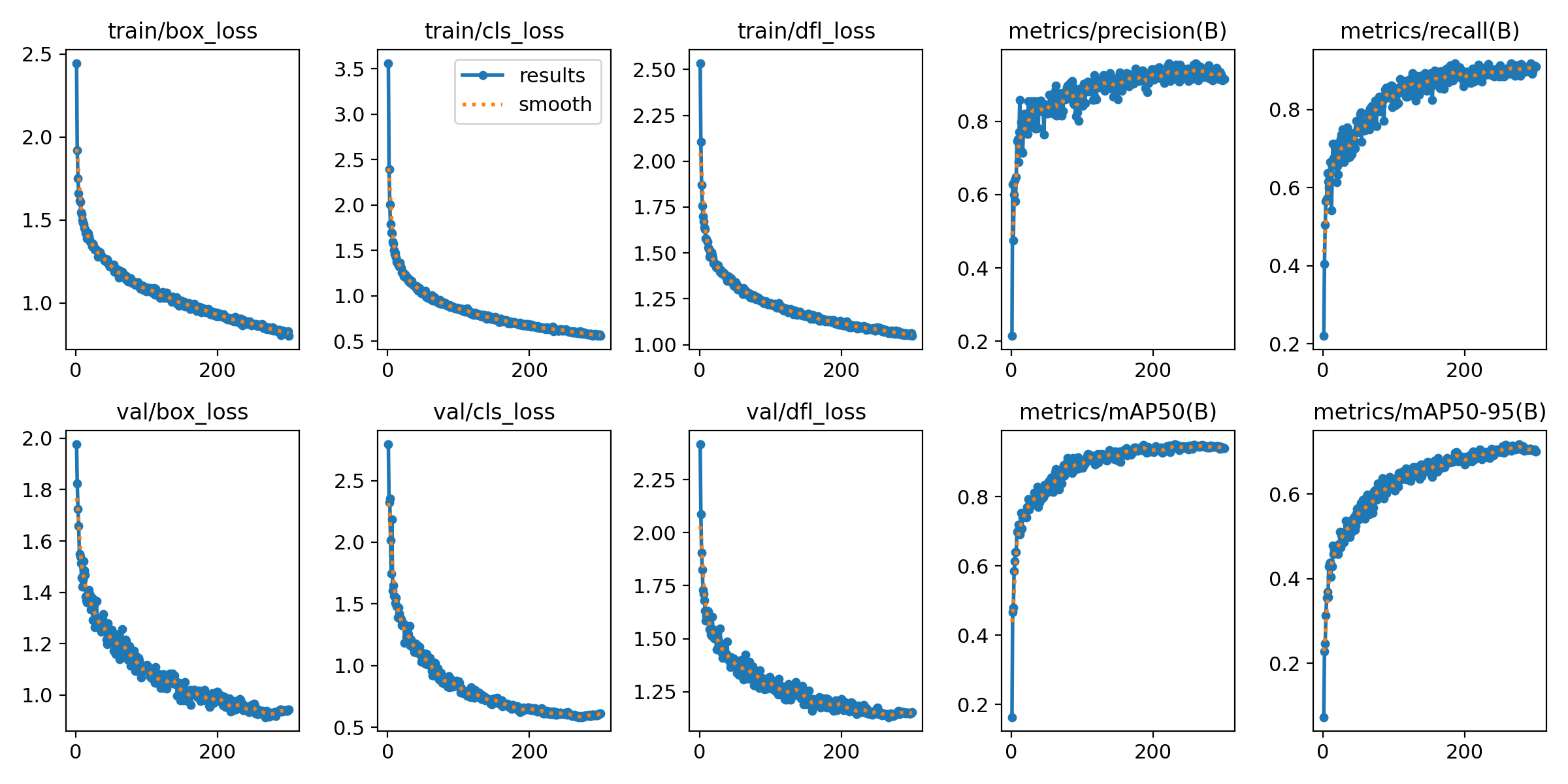

Supplement: Supplementary file 1 — Supplementary Material 1. [file 13007_2026_1527_MOESM1_ESM.zip › same_data/abc-finetune2.0/results.png]

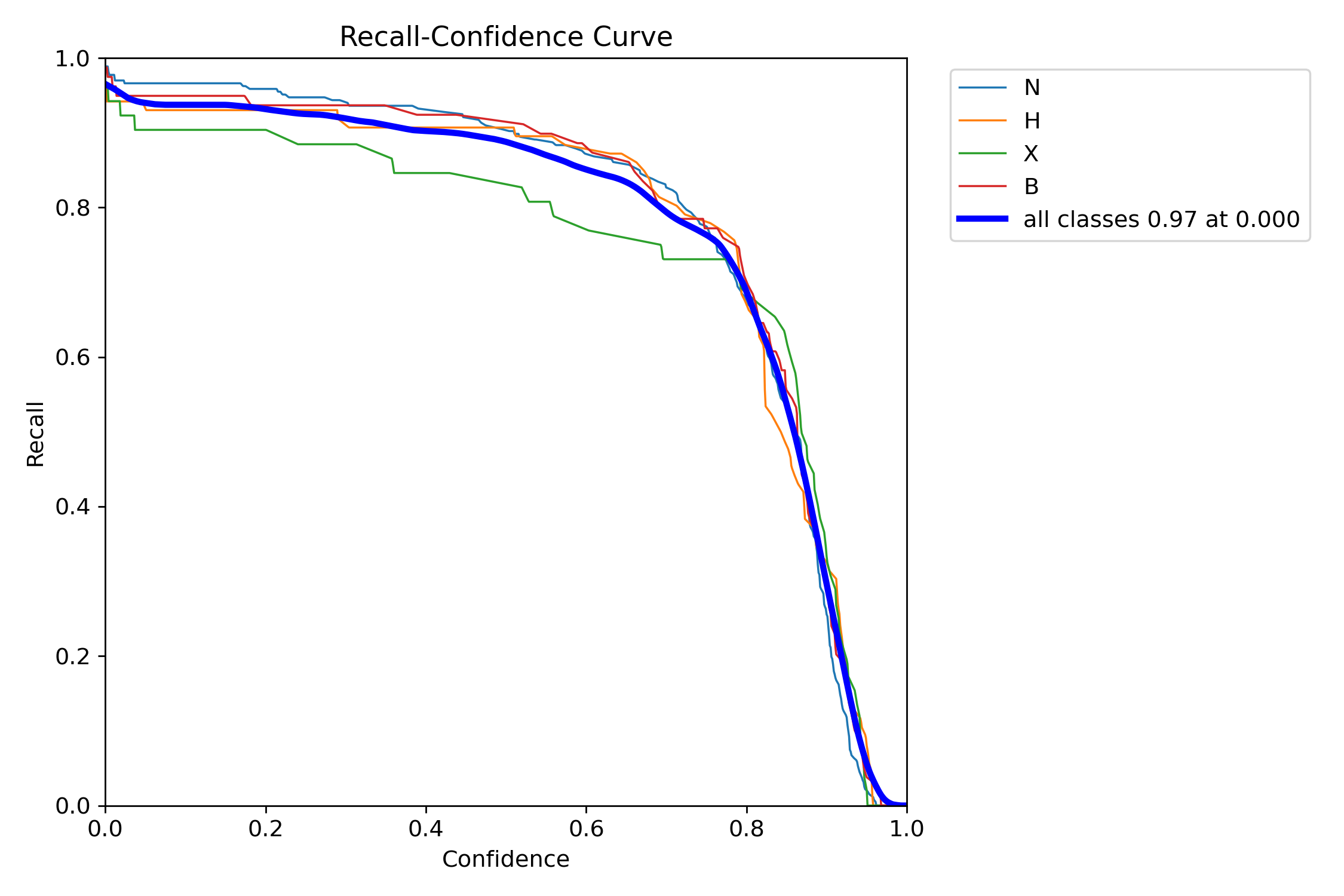

Supplement: Supplementary file 1 — Supplementary Material 1. [file 13007_2026_1527_MOESM1_ESM.zip › same_data/abc-finetune2.0/R_curve.png]

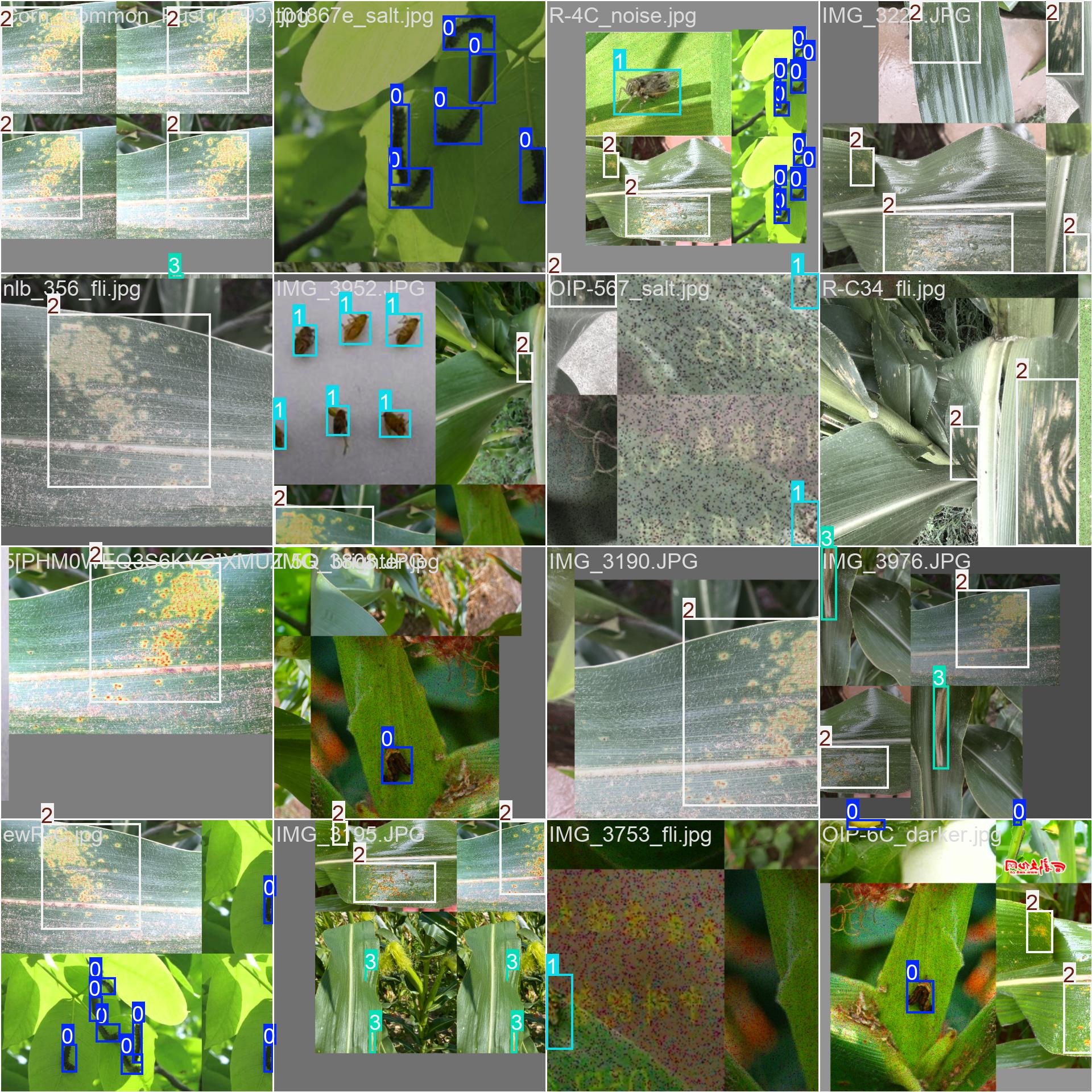

Supplement: Supplementary file 1 — Supplementary Material 1. [file 13007_2026_1527_MOESM1_ESM.zip › same_data/abc-finetune2.0/train_batch0.jpg]

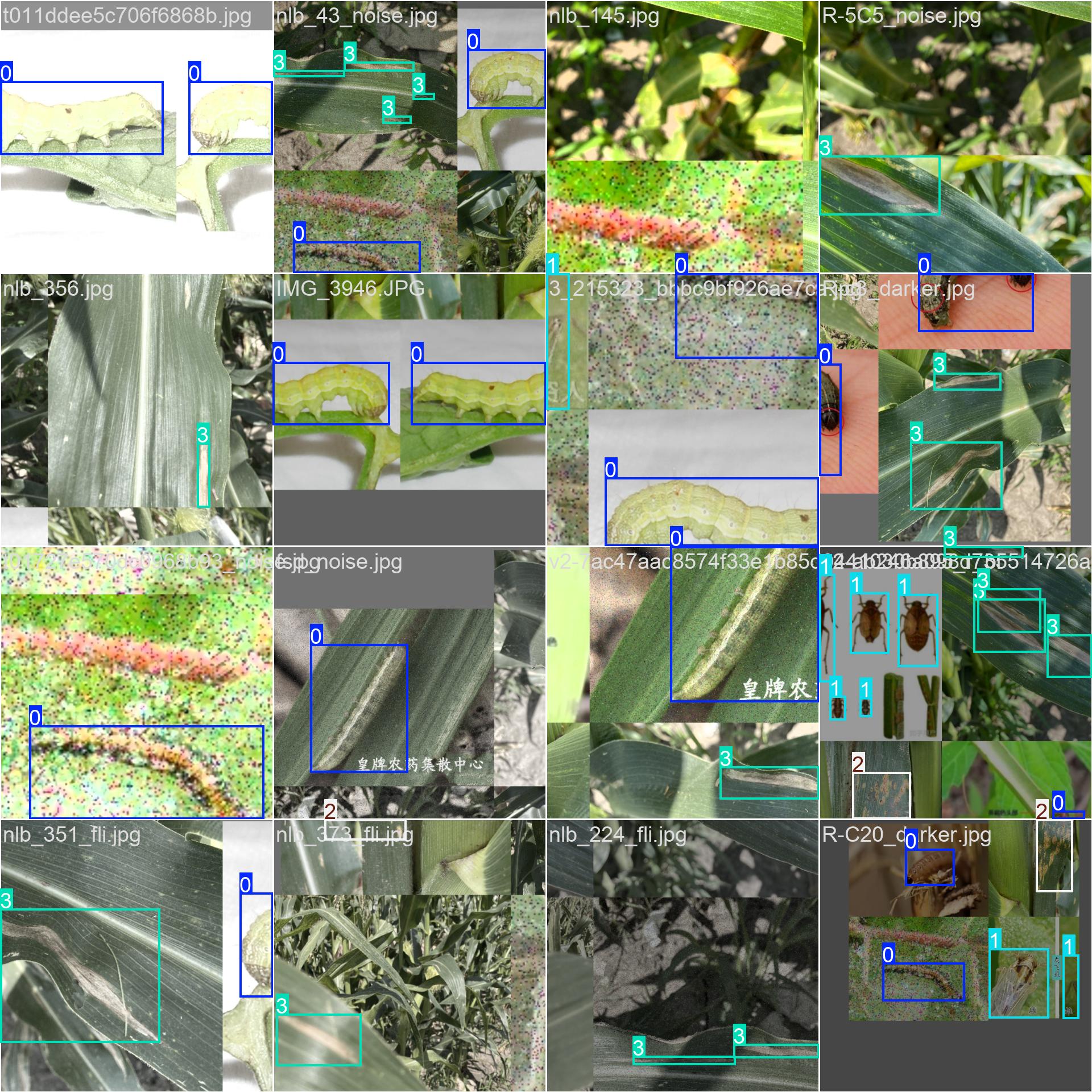

Supplement: Supplementary file 1 — Supplementary Material 1. [file 13007_2026_1527_MOESM1_ESM.zip › same_data/abc-finetune2.0/train_batch1.jpg]

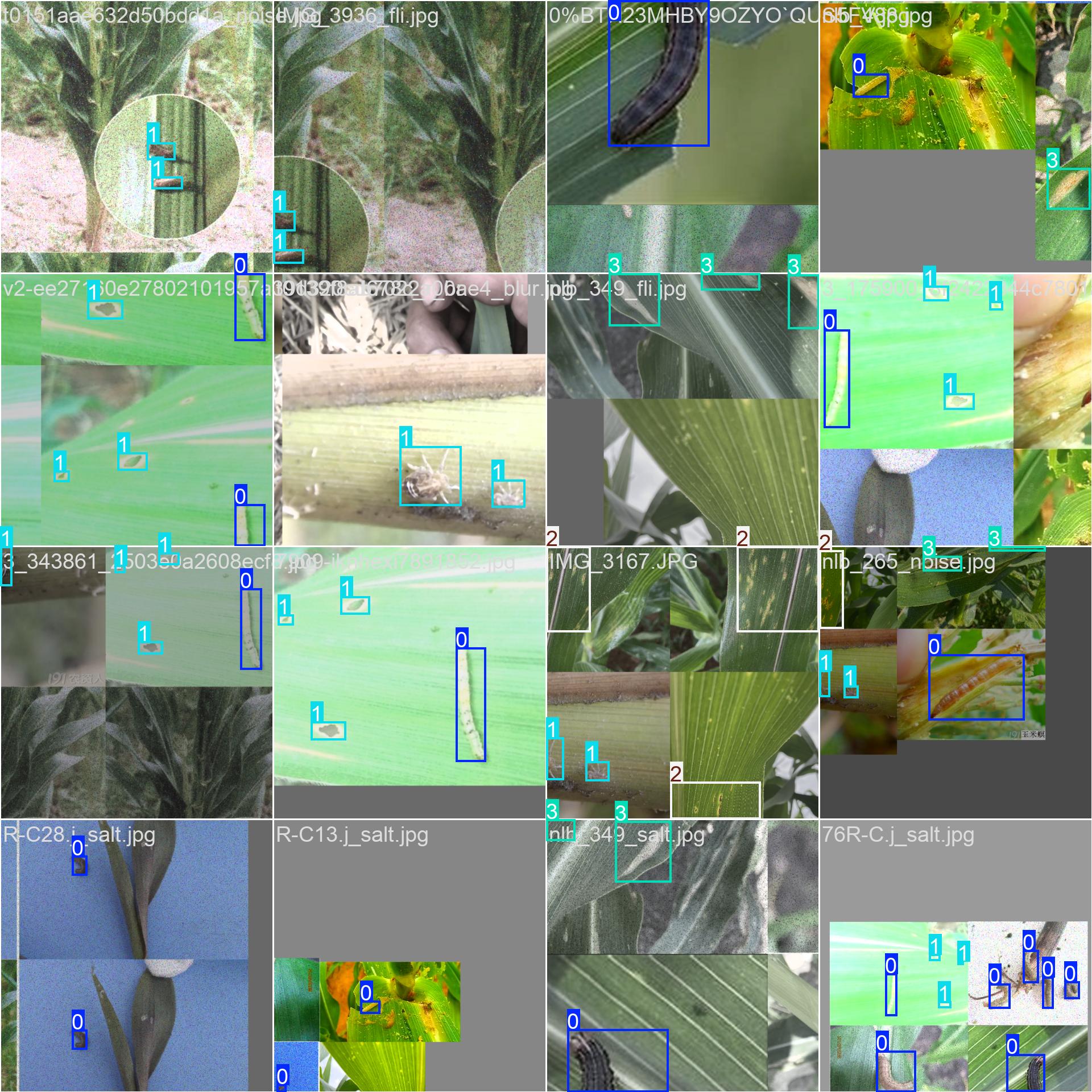

Supplement: Supplementary file 1 — Supplementary Material 1. [file 13007_2026_1527_MOESM1_ESM.zip › same_data/abc-finetune2.0/train_batch2.jpg]

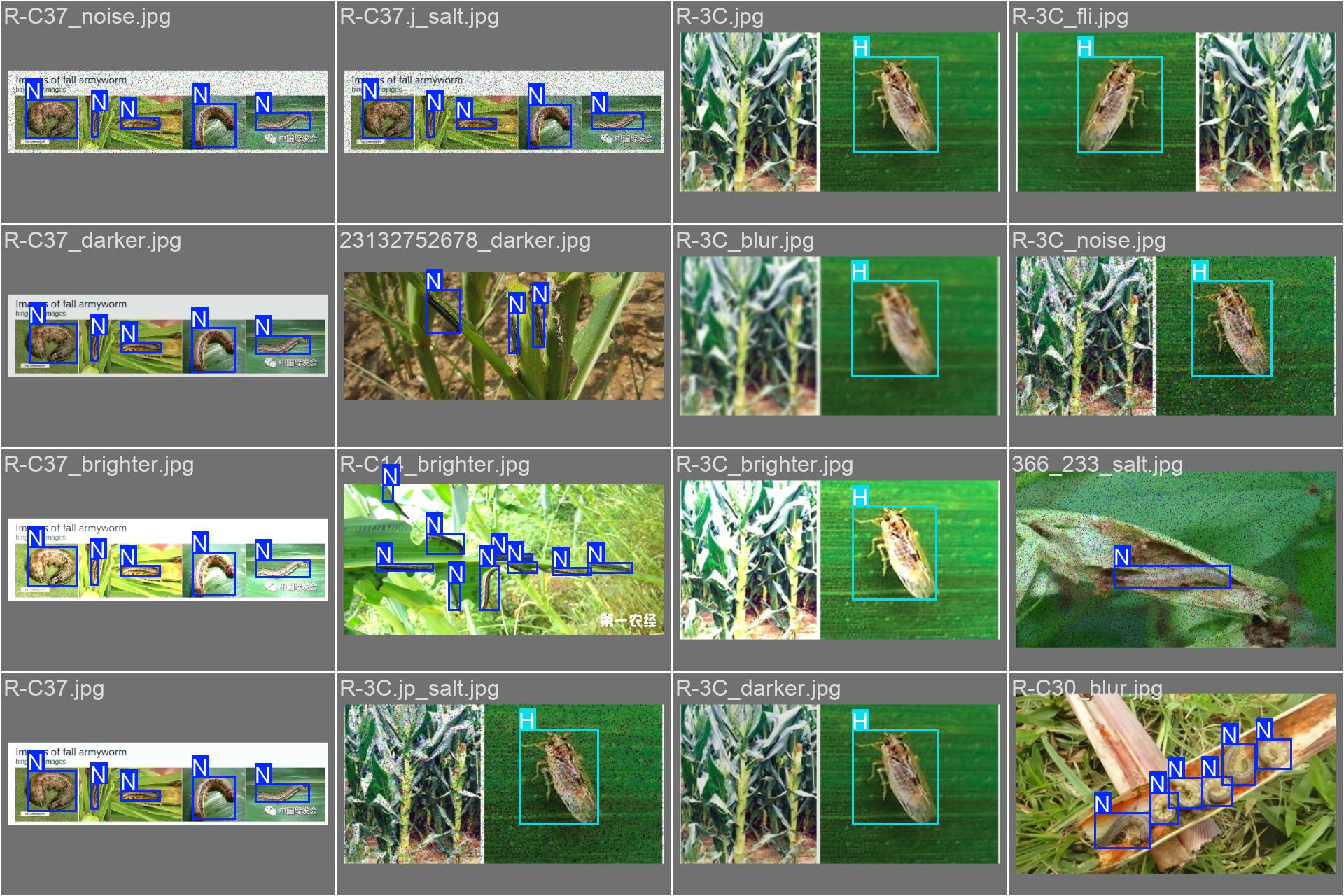

Supplement: Supplementary file 1 — Supplementary Material 1. [file 13007_2026_1527_MOESM1_ESM.zip › same_data/abc-finetune2.0/val_batch0_labels.jpg]

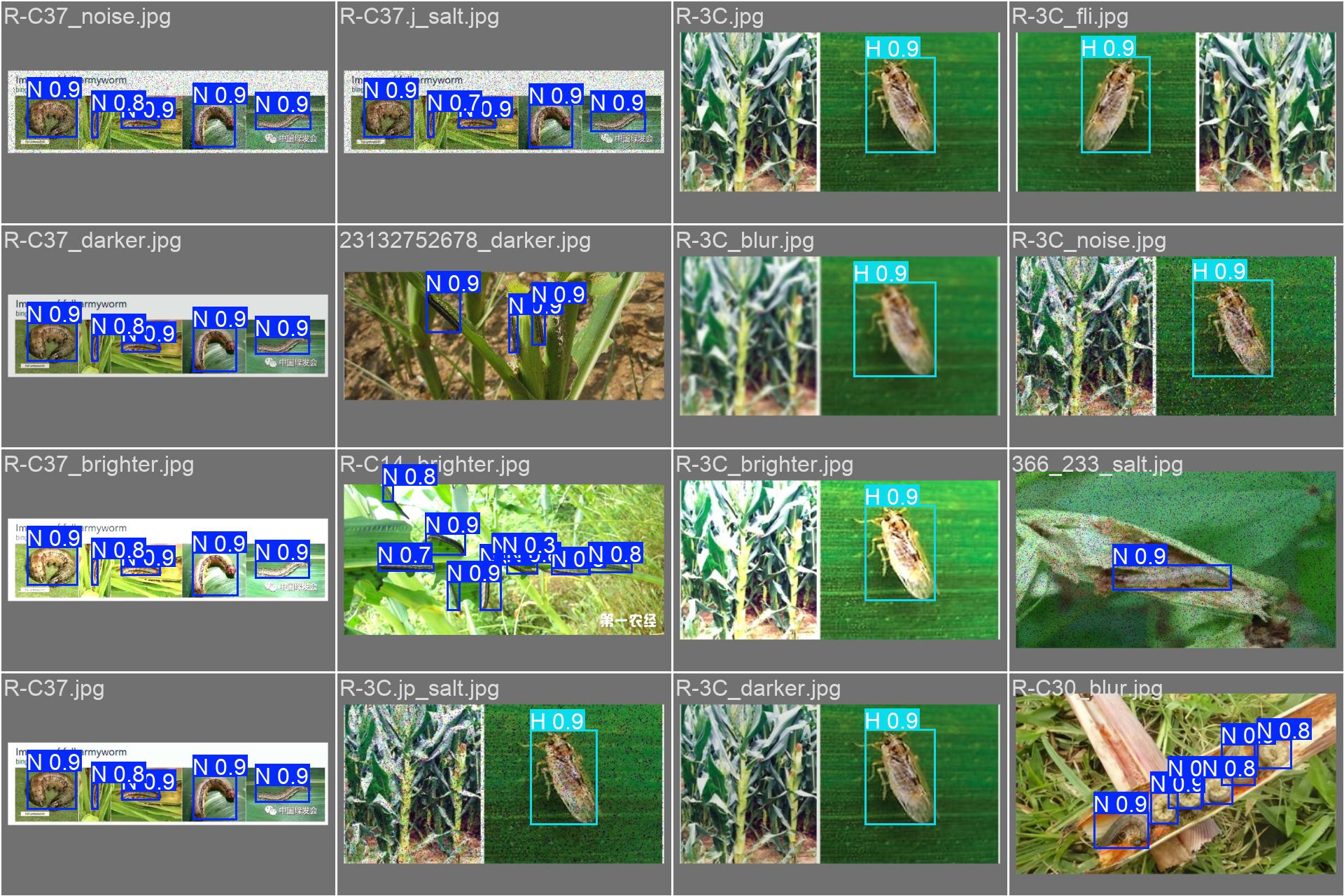

Supplement: Supplementary file 1 — Supplementary Material 1. [file 13007_2026_1527_MOESM1_ESM.zip › same_data/abc-finetune2.0/val_batch0_pred.jpg]

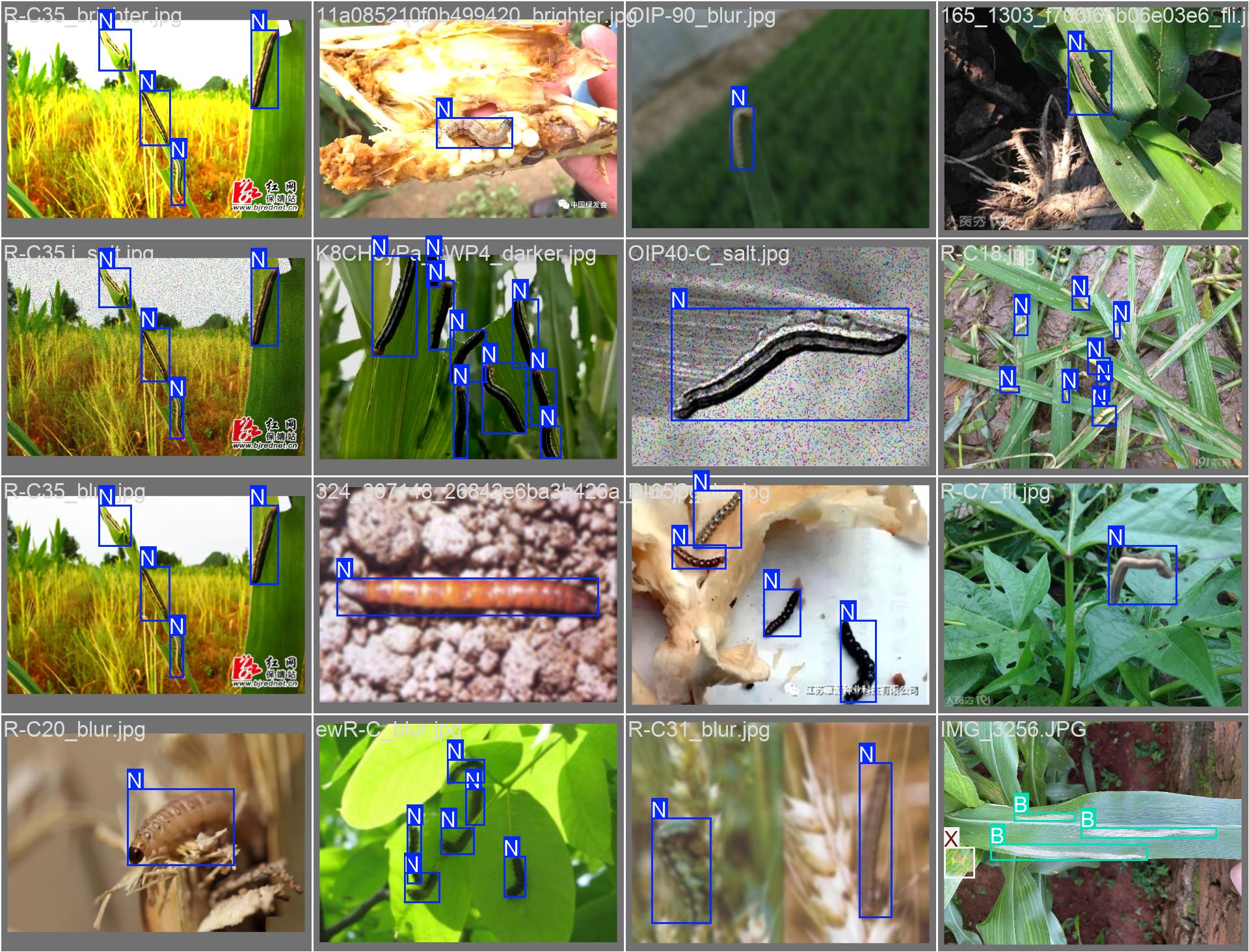

Supplement: Supplementary file 1 — Supplementary Material 1. [file 13007_2026_1527_MOESM1_ESM.zip › same_data/abc-finetune2.0/val_batch1_labels.jpg]

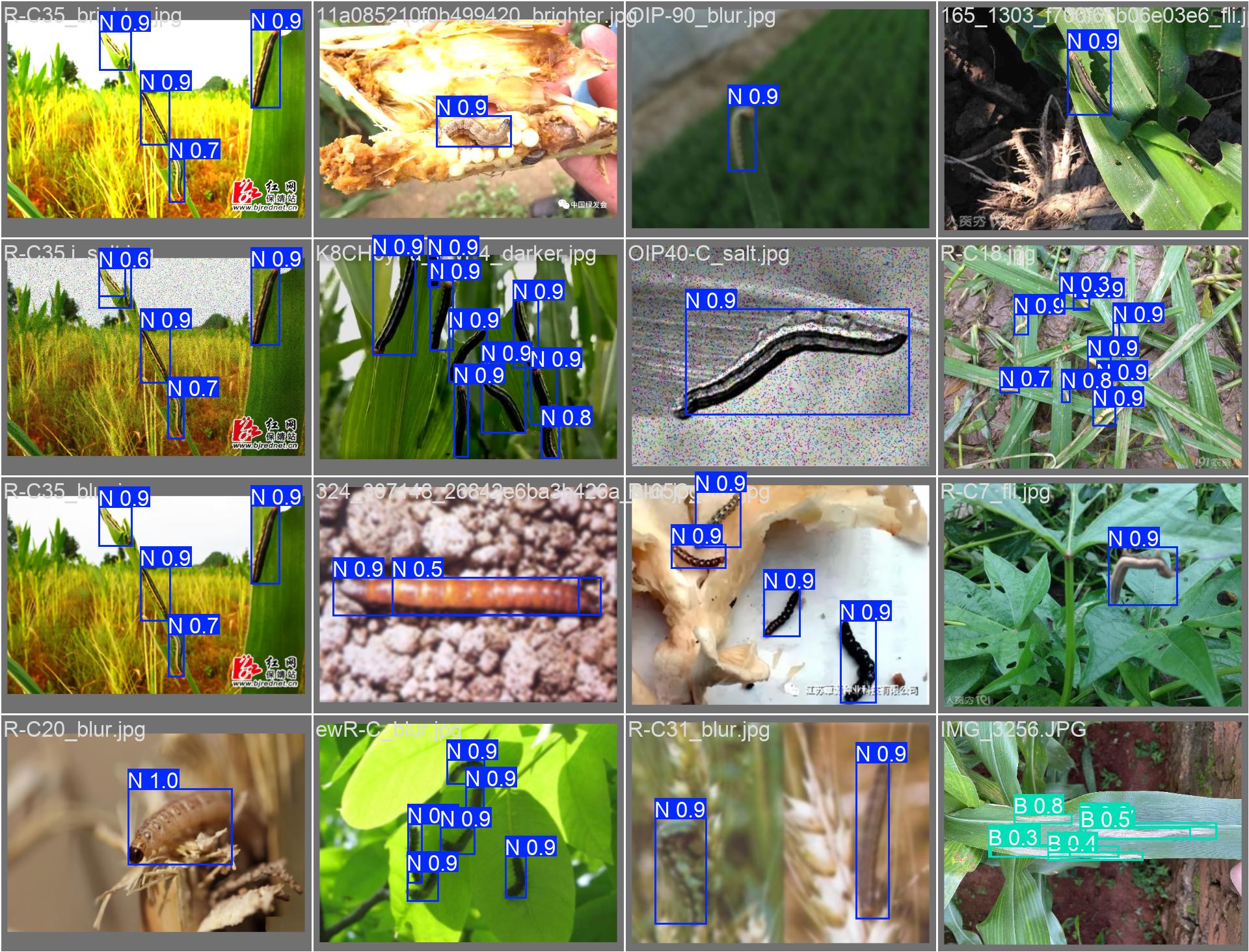

Supplement: Supplementary file 1 — Supplementary Material 1. [file 13007_2026_1527_MOESM1_ESM.zip › same_data/abc-finetune2.0/val_batch1_pred.jpg]

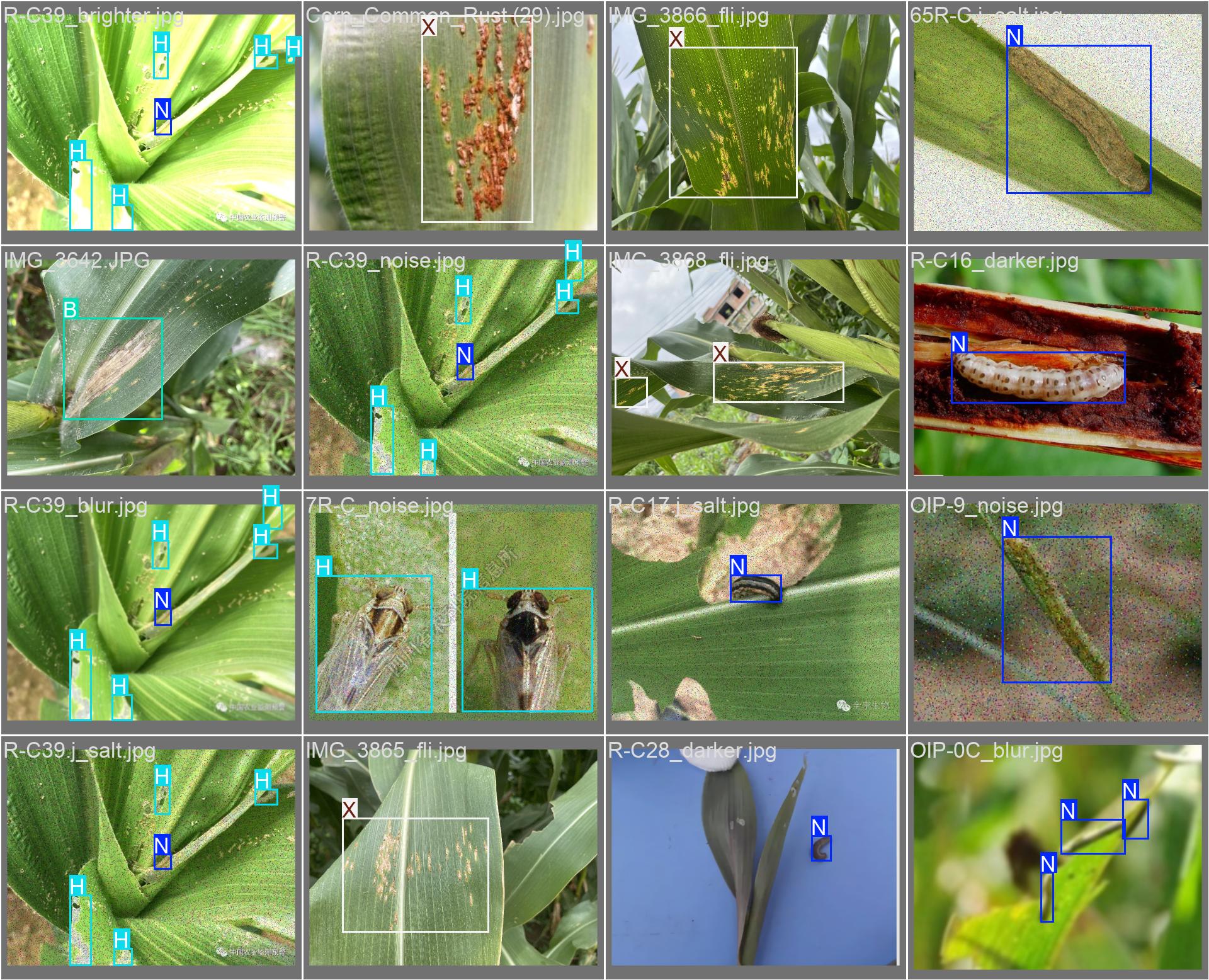

Supplement: Supplementary file 1 — Supplementary Material 1. [file 13007_2026_1527_MOESM1_ESM.zip › same_data/abc-finetune2.0/val_batch2_labels.jpg]

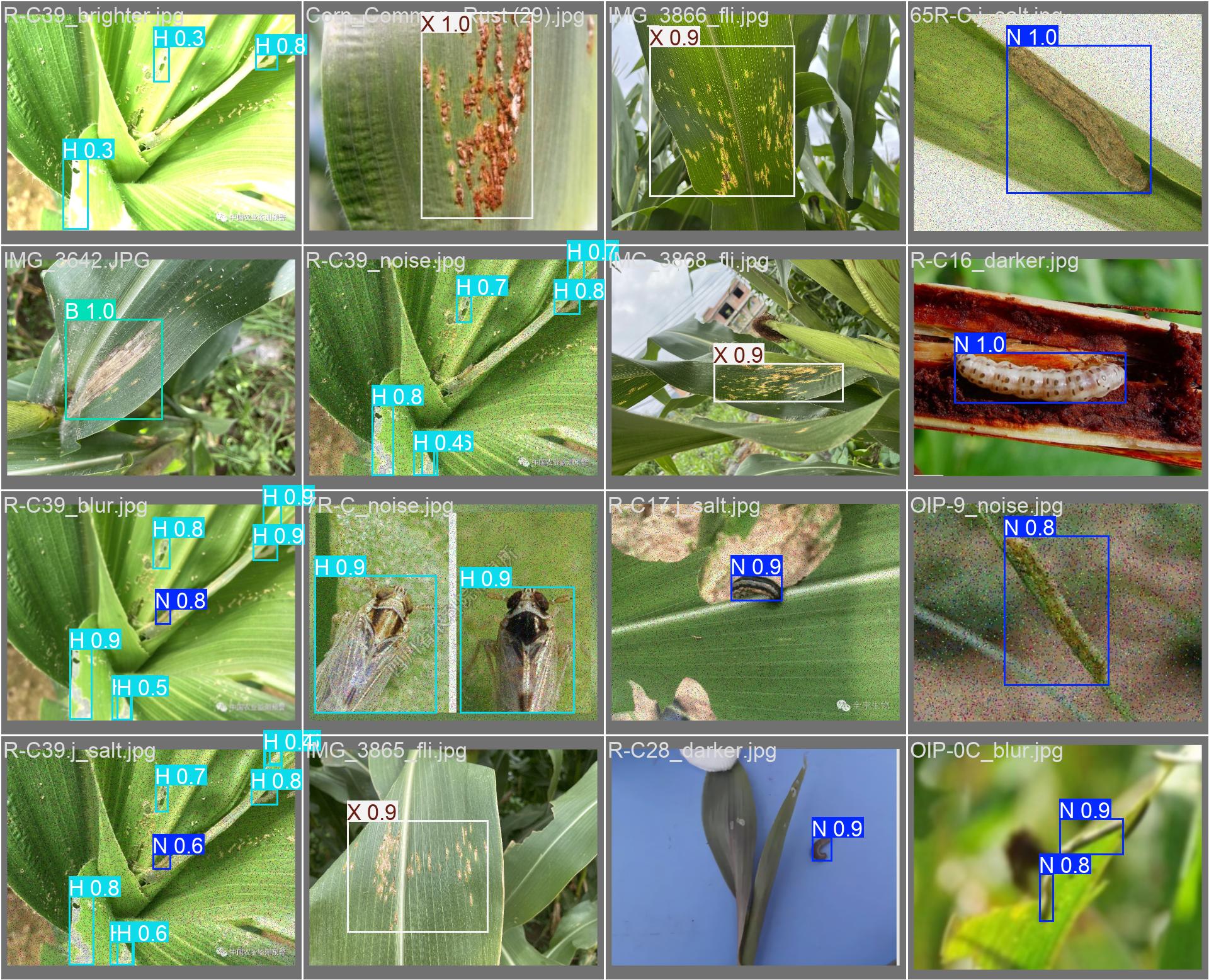

Supplement: Supplementary file 1 — Supplementary Material 1. [file 13007_2026_1527_MOESM1_ESM.zip › same_data/abc-finetune2.0/val_batch2_pred.jpg]

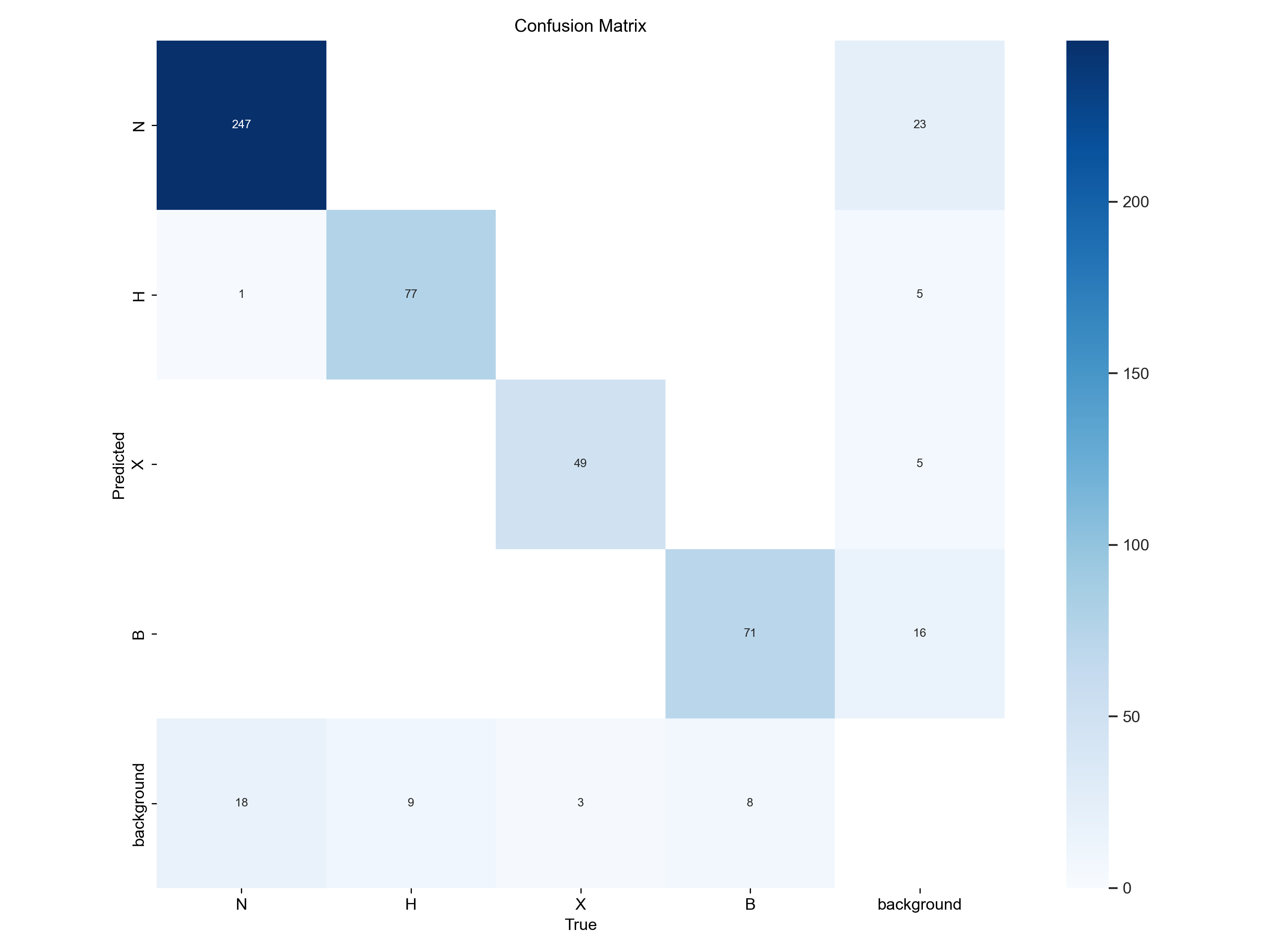

Supplement: Supplementary file 1 — Supplementary Material 1. [file 13007_2026_1527_MOESM1_ESM.zip › same_data/abc3/confusion_matrix.png]

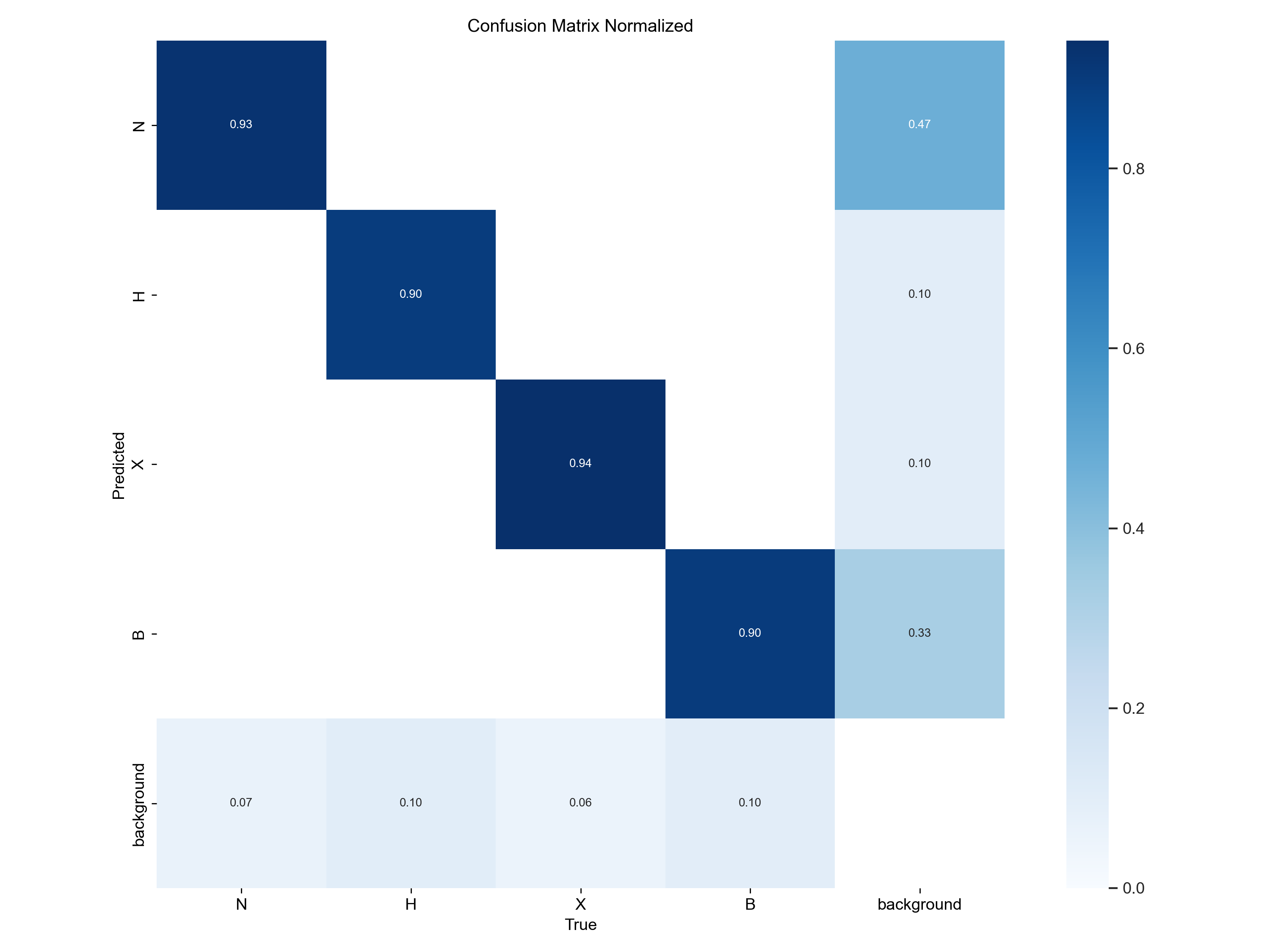

Supplement: Supplementary file 1 — Supplementary Material 1. [file 13007_2026_1527_MOESM1_ESM.zip › same_data/abc3/confusion_matrix_normalized.png]

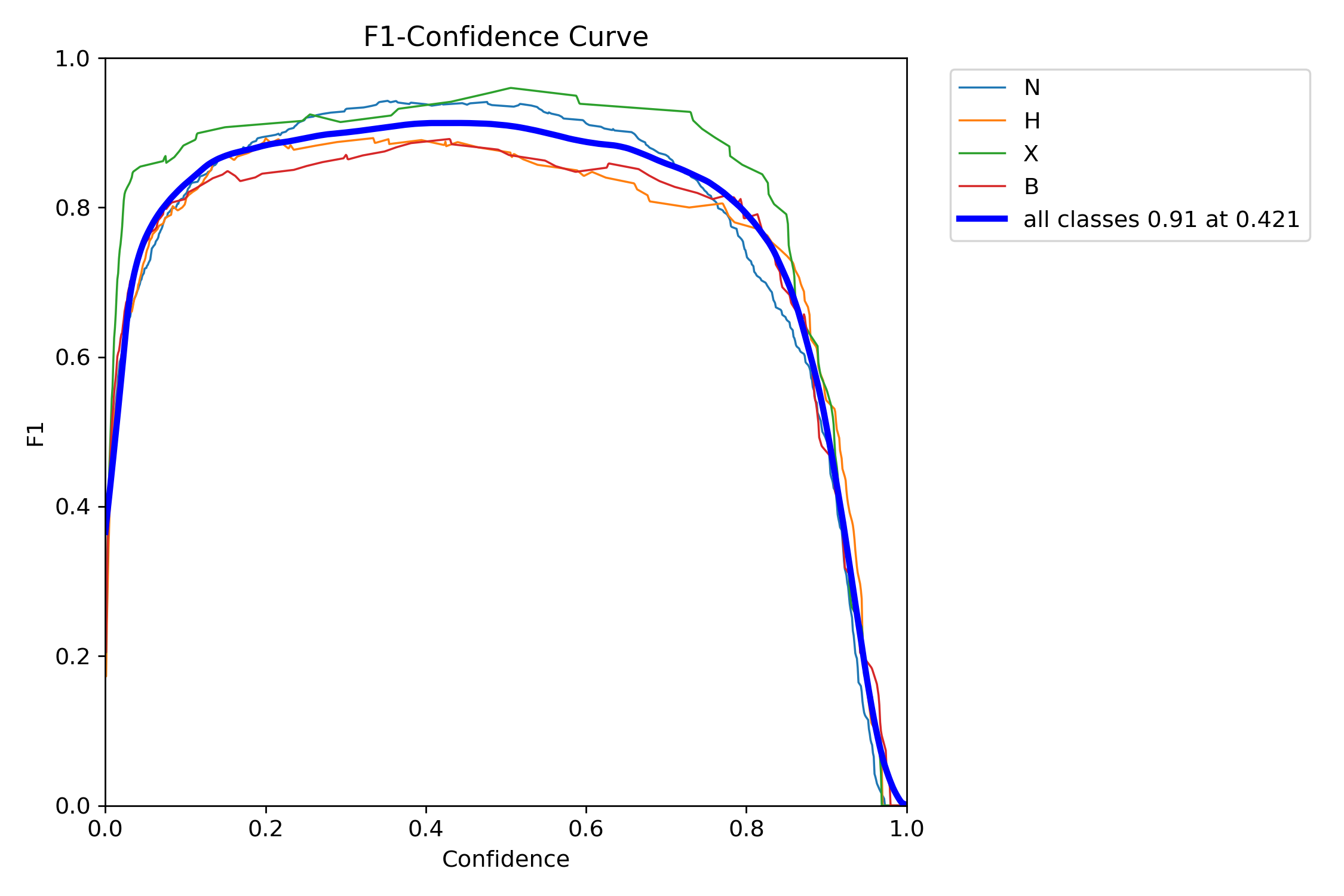

Supplement: Supplementary file 1 — Supplementary Material 1. [file 13007_2026_1527_MOESM1_ESM.zip › same_data/abc3/F1_curve.png]

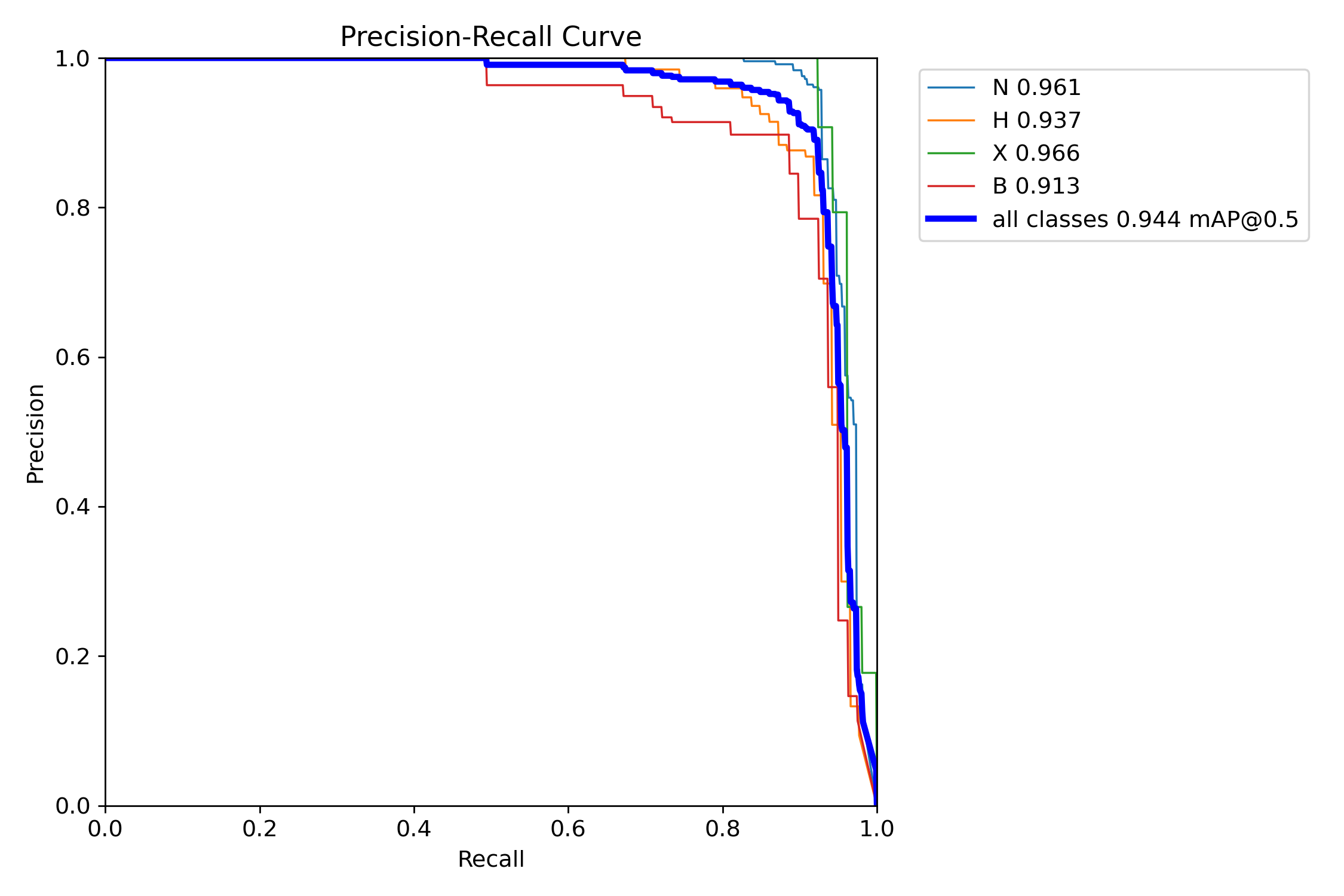

Supplement: Supplementary file 1 — Supplementary Material 1. [file 13007_2026_1527_MOESM1_ESM.zip › same_data/abc3/PR_curve.png]

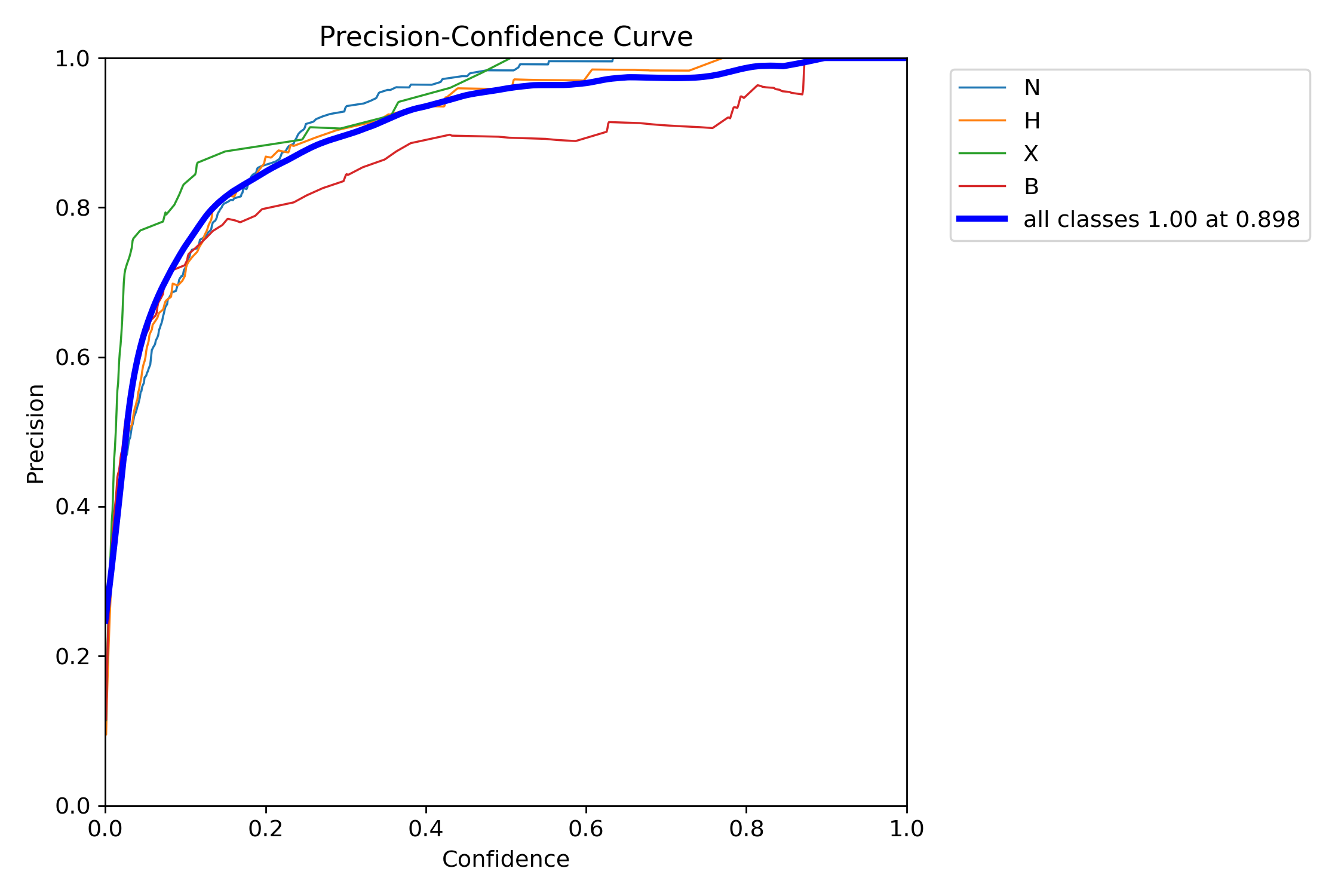

Supplement: Supplementary file 1 — Supplementary Material 1. [file 13007_2026_1527_MOESM1_ESM.zip › same_data/abc3/P_curve.png]

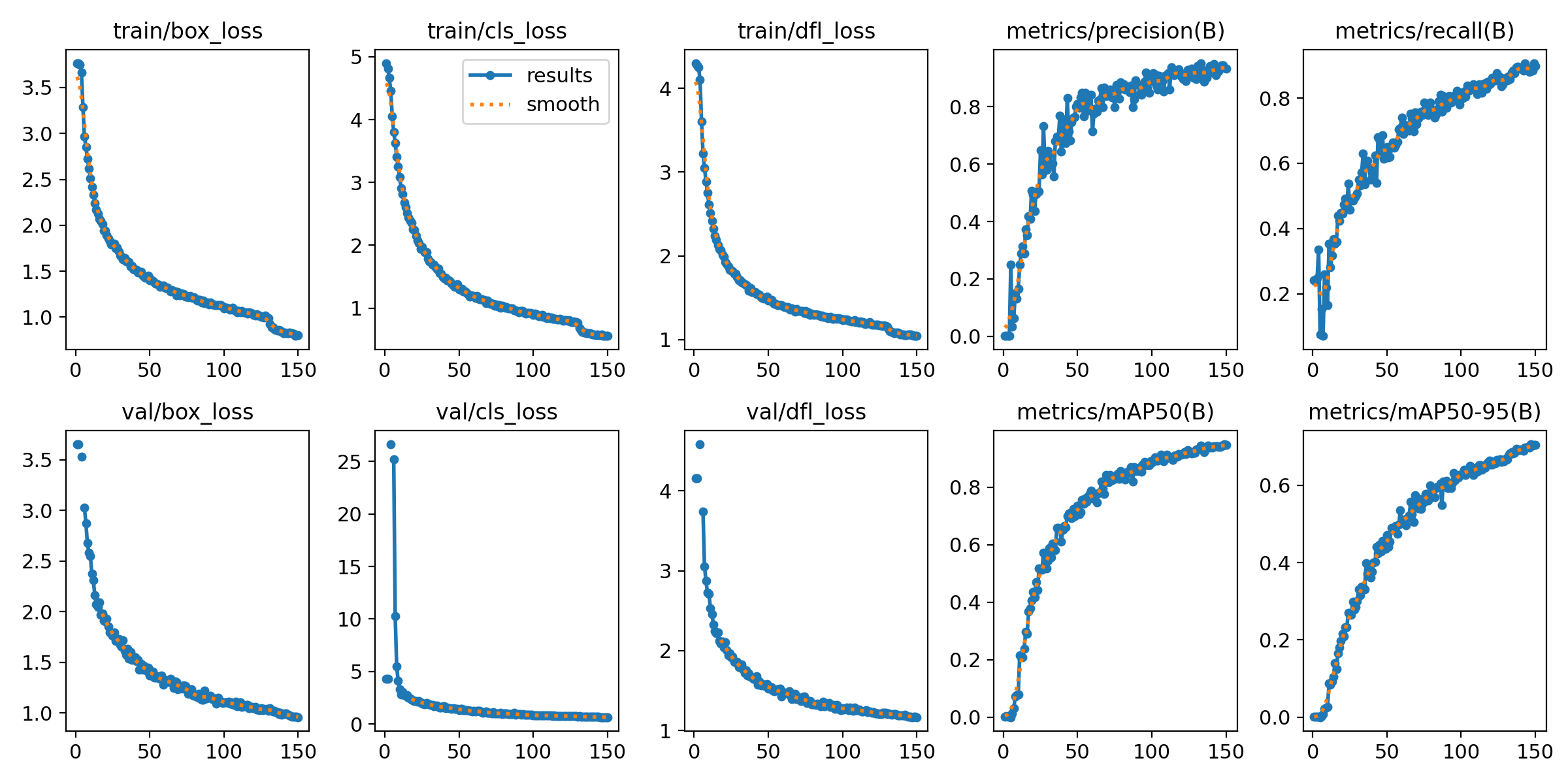

Supplement: Supplementary file 1 — Supplementary Material 1. [file 13007_2026_1527_MOESM1_ESM.zip › same_data/abc3/results.png]

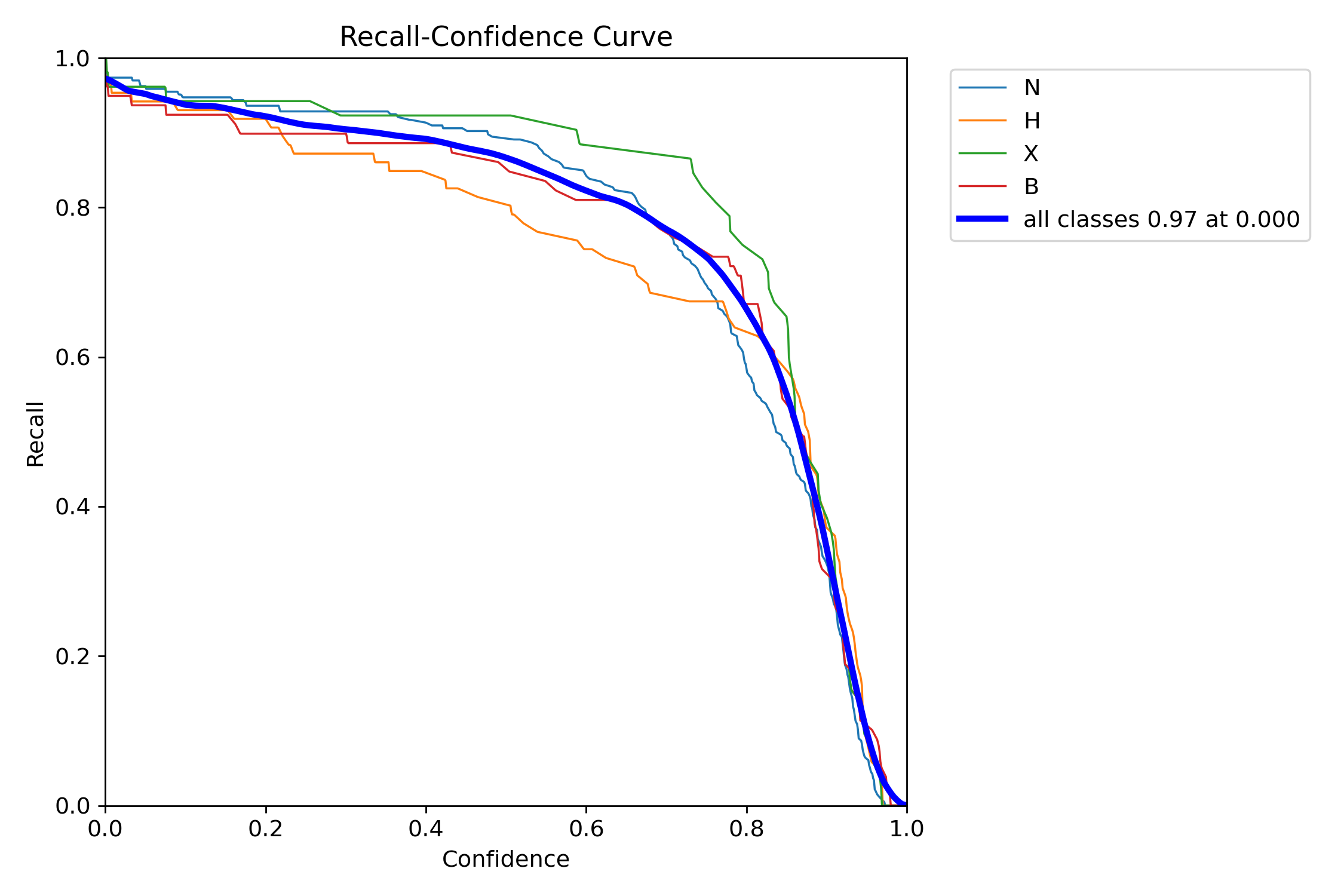

Supplement: Supplementary file 1 — Supplementary Material 1. [file 13007_2026_1527_MOESM1_ESM.zip › same_data/abc3/R_curve.png]

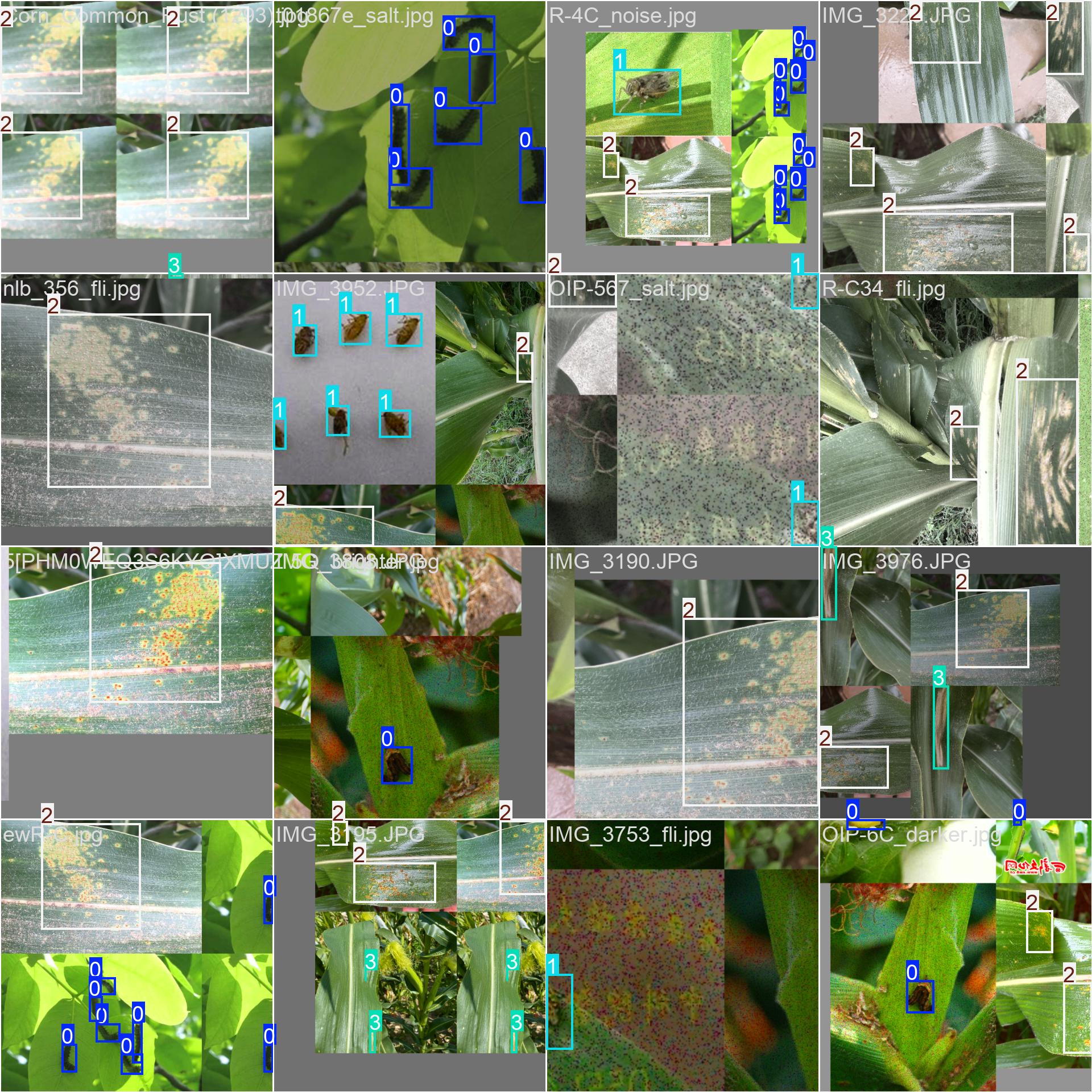

Supplement: Supplementary file 1 — Supplementary Material 1. [file 13007_2026_1527_MOESM1_ESM.zip › same_data/abc3/train_batch0.jpg]

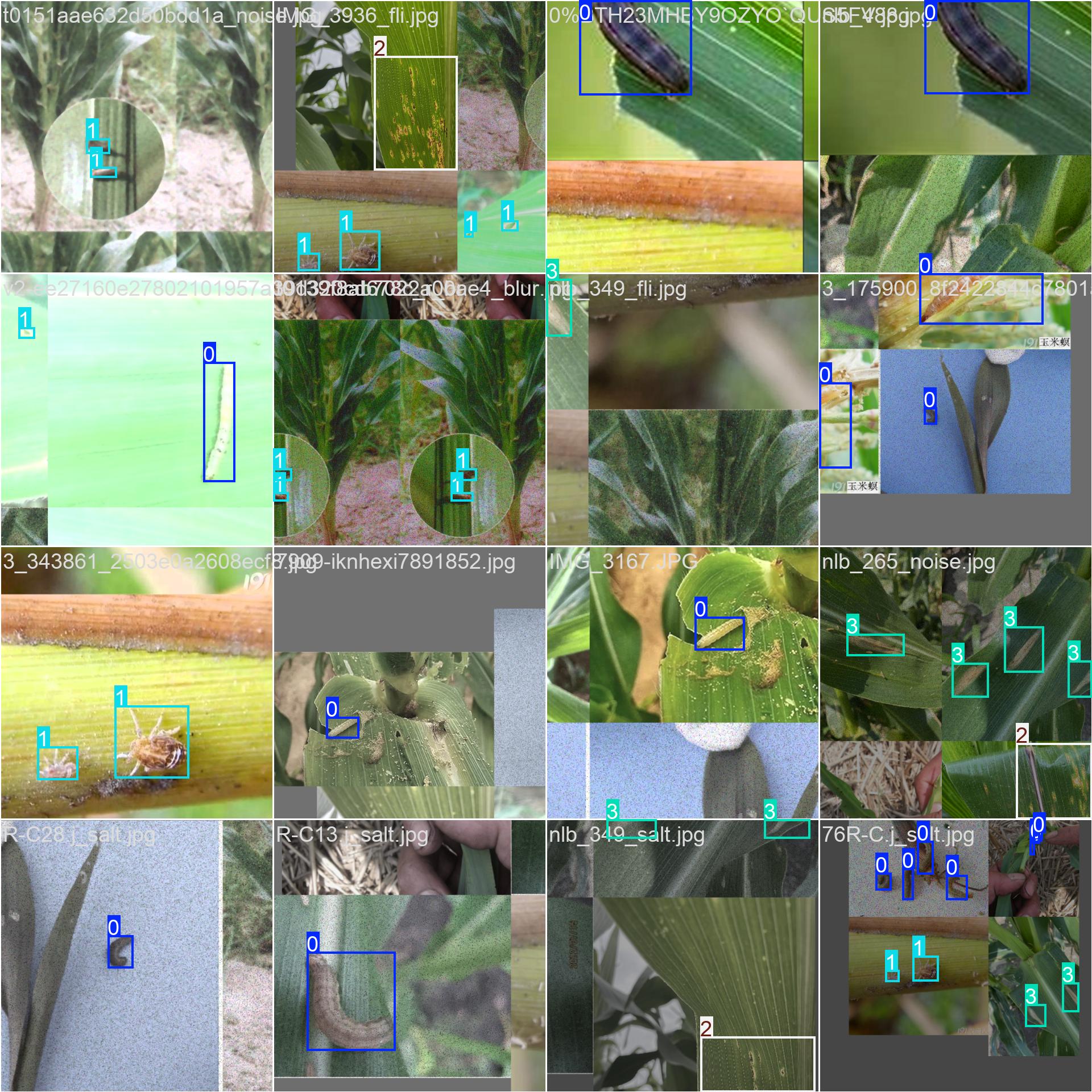

Supplement: Supplementary file 1 — Supplementary Material 1. [file 13007_2026_1527_MOESM1_ESM.zip › same_data/abc3/train_batch1.jpg]

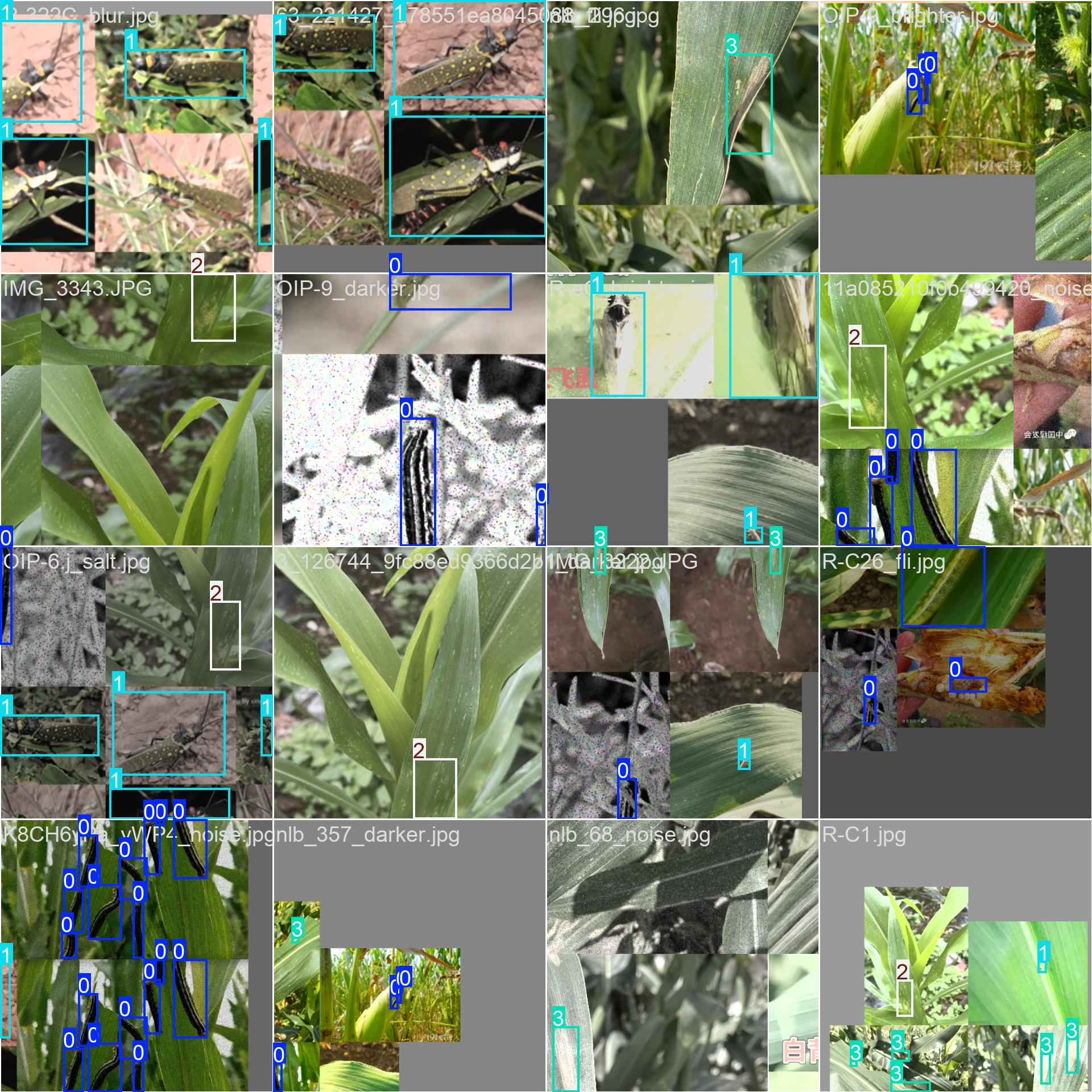

Supplement: Supplementary file 1 — Supplementary Material 1. [file 13007_2026_1527_MOESM1_ESM.zip › same_data/abc3/train_batch2.jpg]

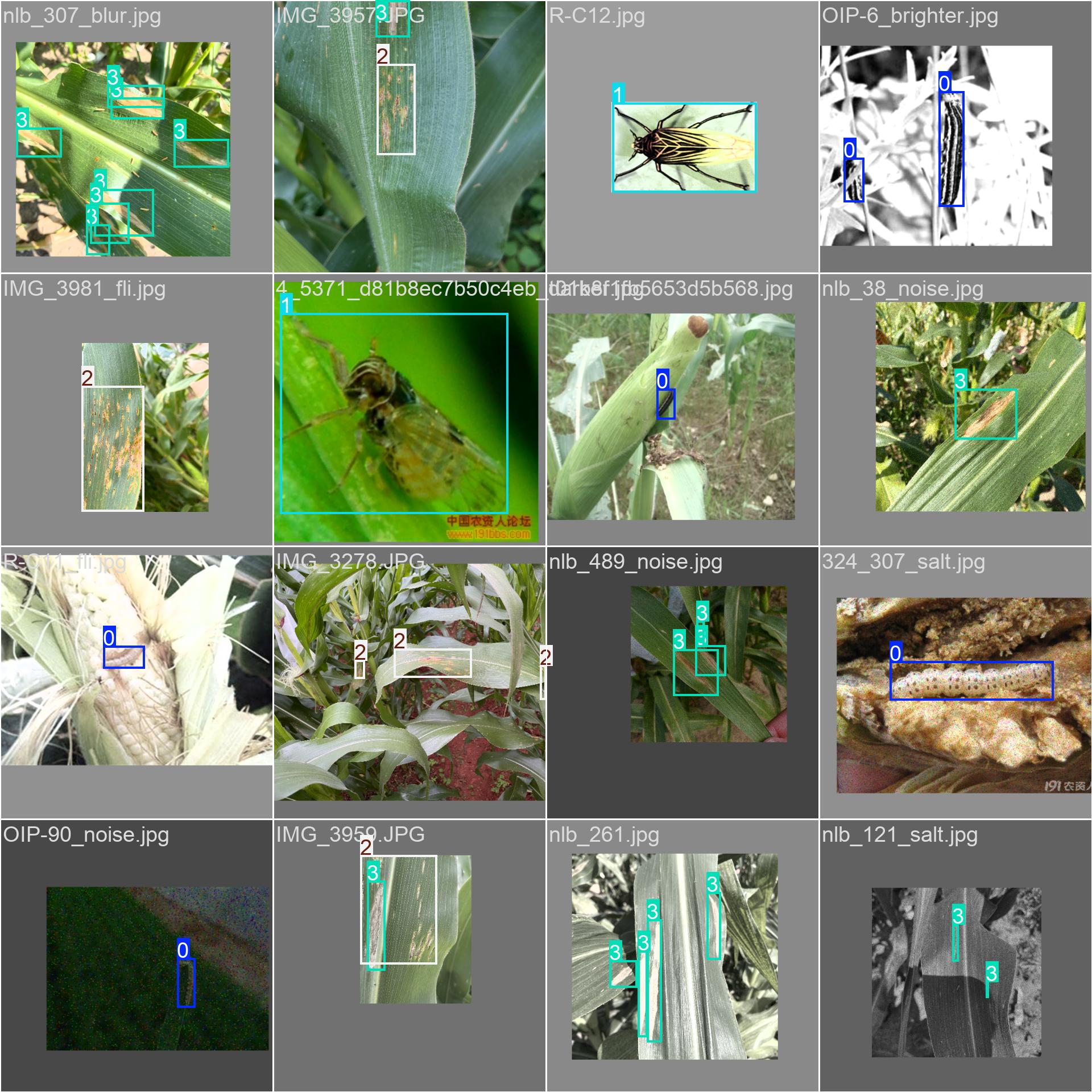

Supplement: Supplementary file 1 — Supplementary Material 1. [file 13007_2026_1527_MOESM1_ESM.zip › same_data/abc3/train_batch6500.jpg]

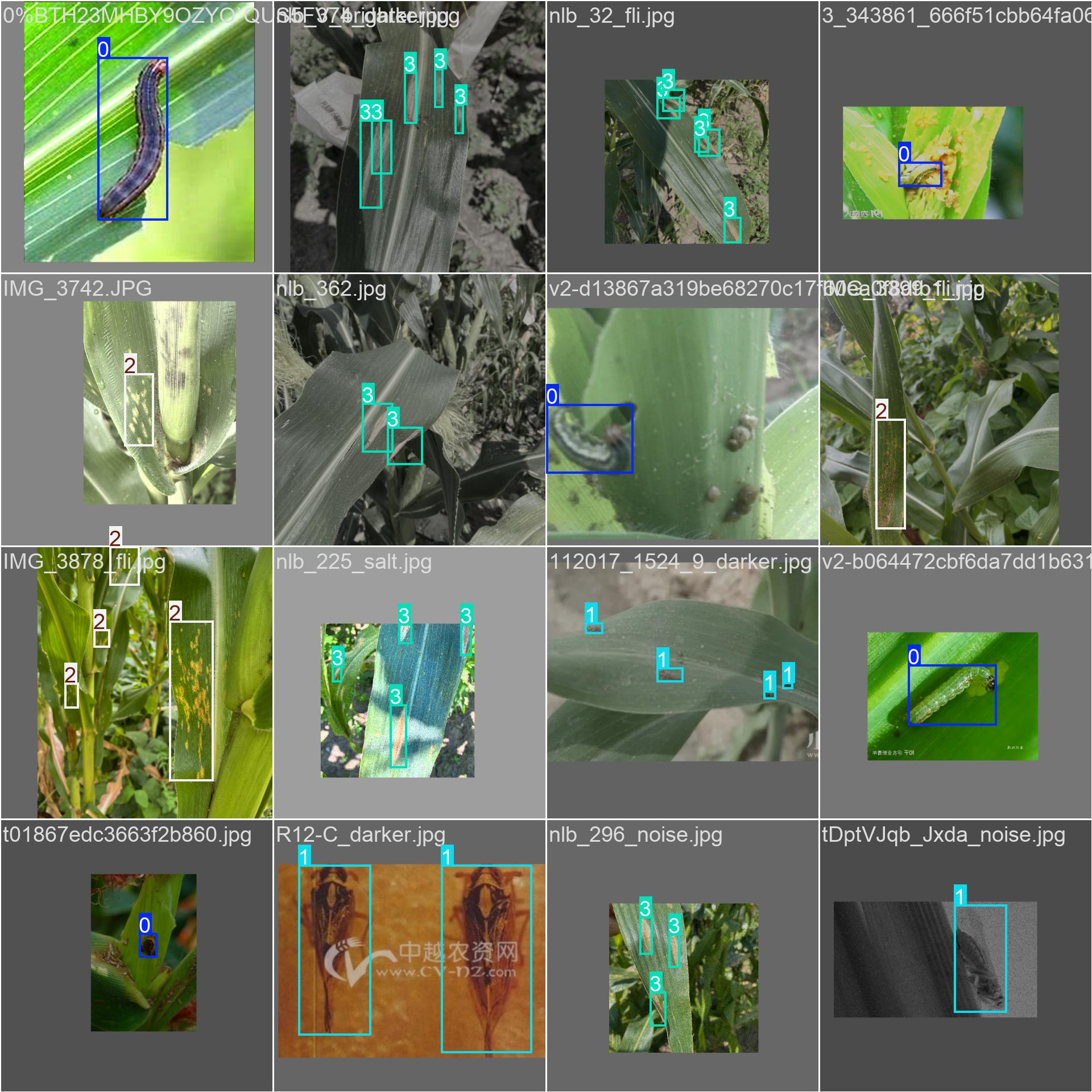

Supplement: Supplementary file 1 — Supplementary Material 1. [file 13007_2026_1527_MOESM1_ESM.zip › same_data/abc3/train_batch6501.jpg]

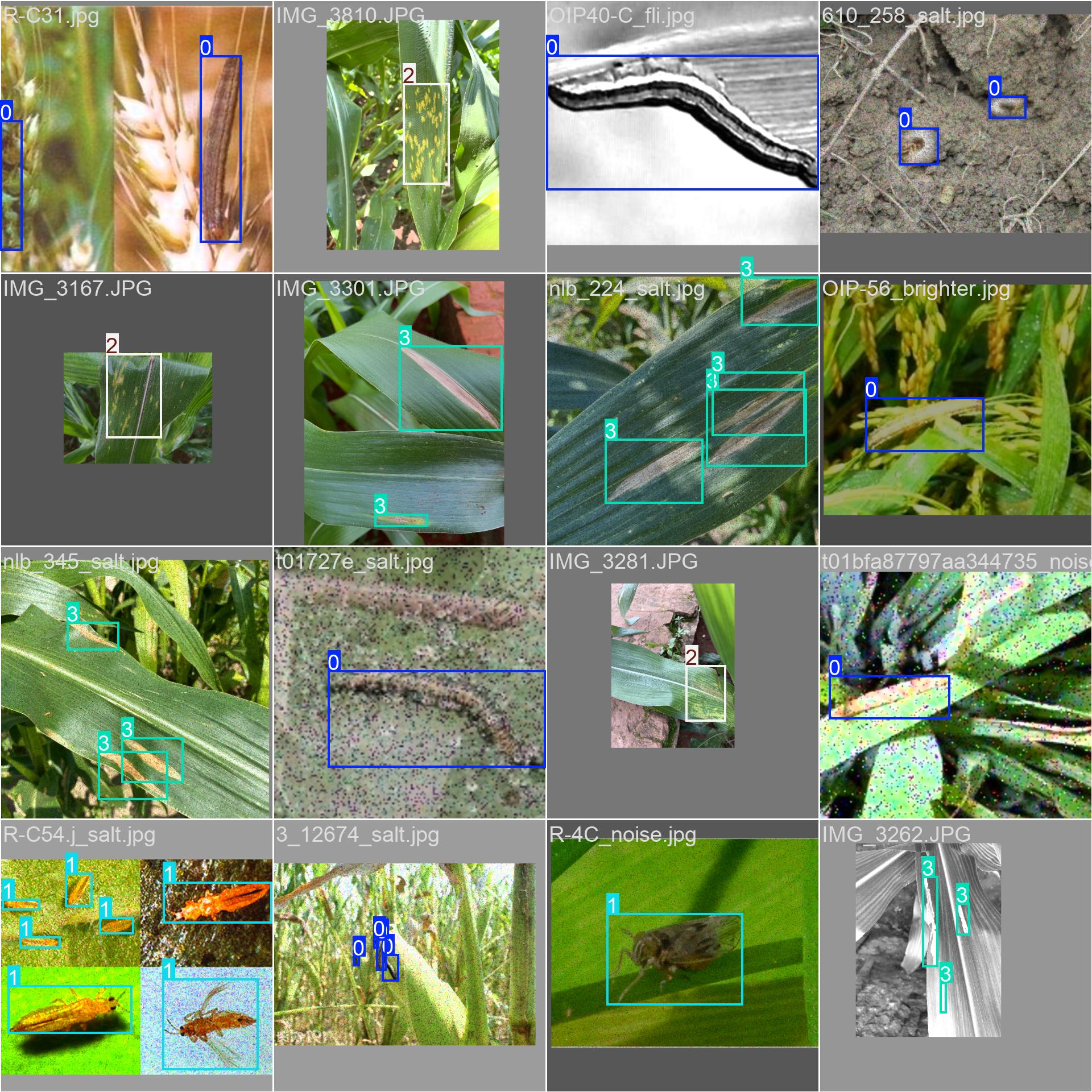

Supplement: Supplementary file 1 — Supplementary Material 1. [file 13007_2026_1527_MOESM1_ESM.zip › same_data/abc3/train_batch6502.jpg]

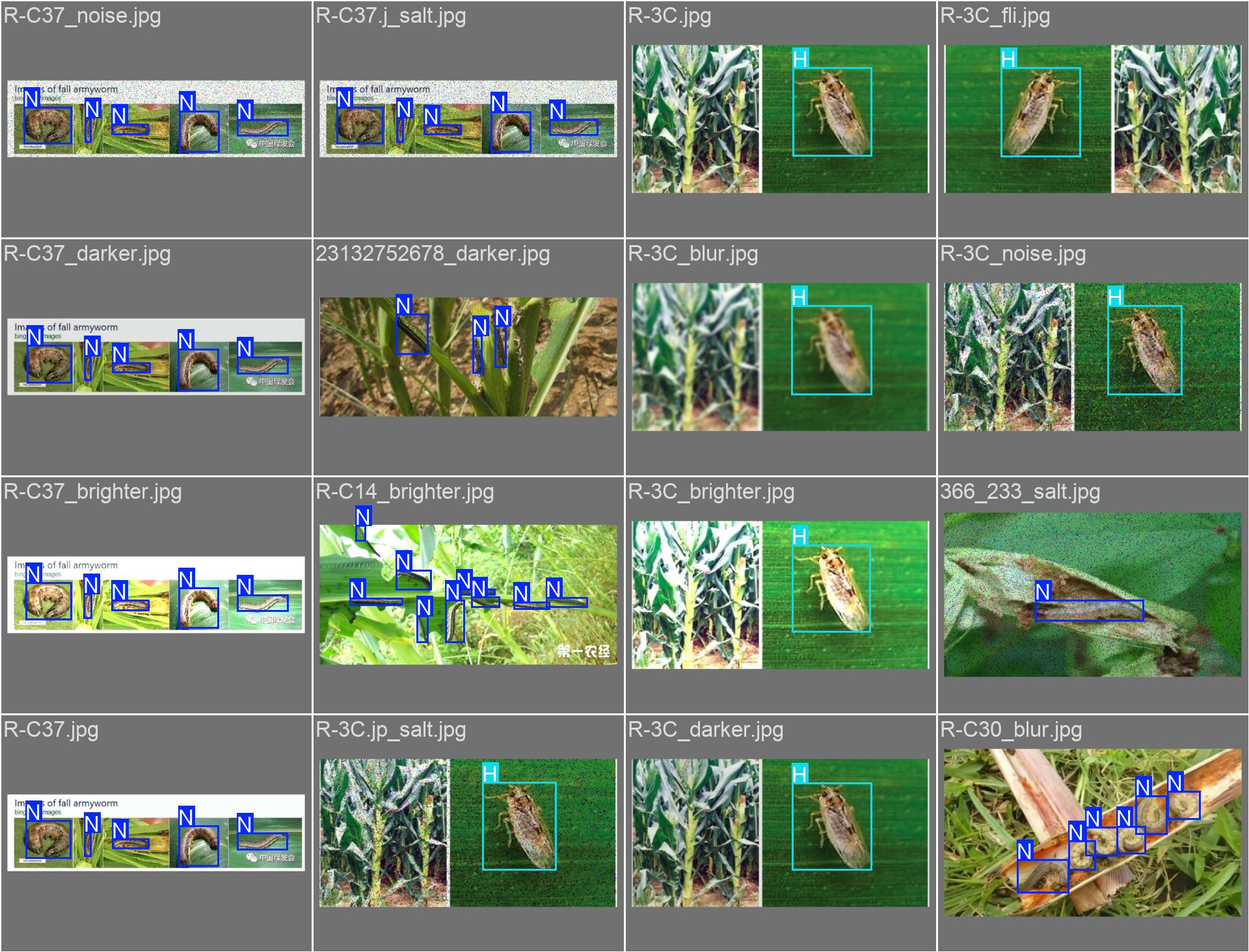

Supplement: Supplementary file 1 — Supplementary Material 1. [file 13007_2026_1527_MOESM1_ESM.zip › same_data/abc3/val_batch0_labels.jpg]

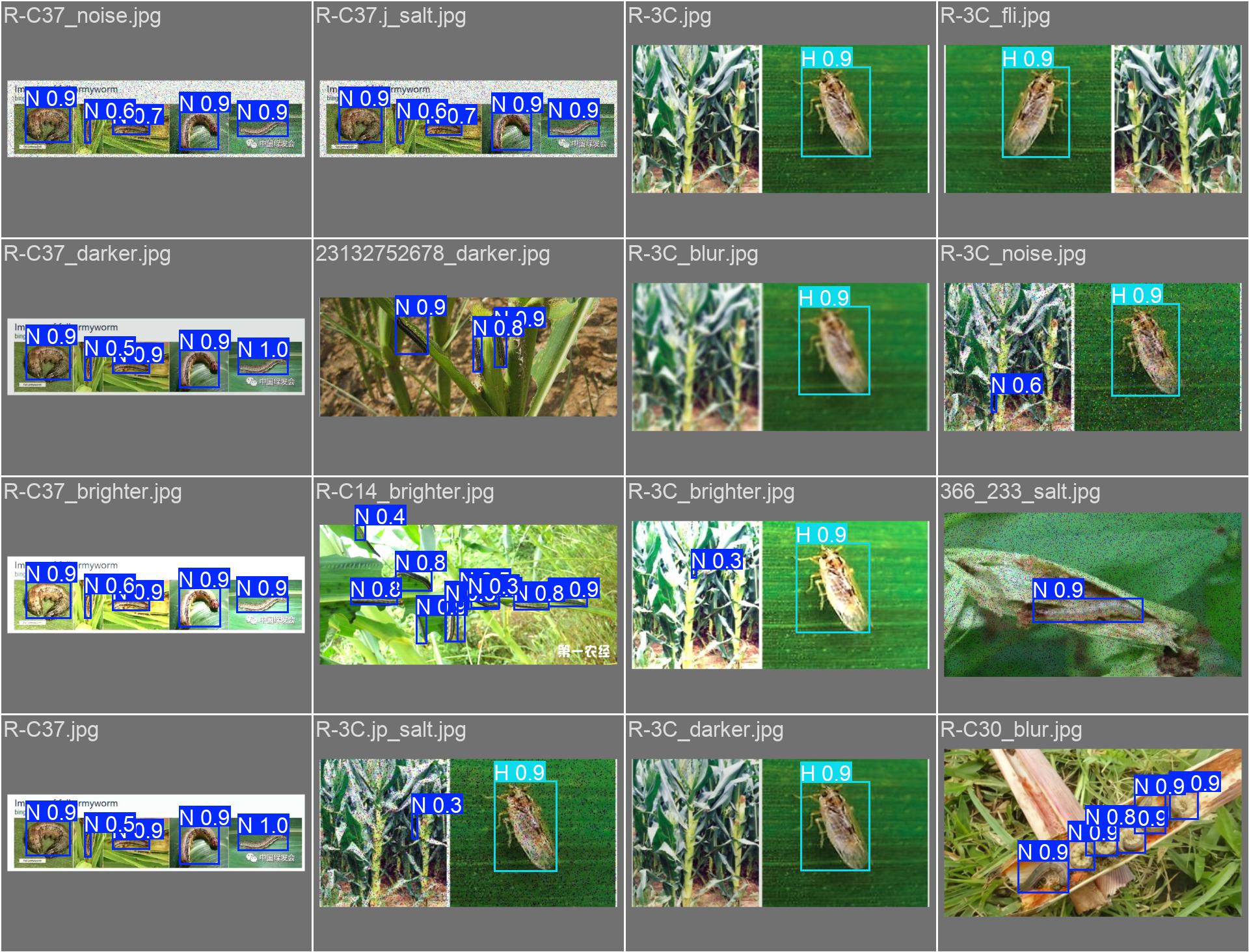

Supplement: Supplementary file 1 — Supplementary Material 1. [file 13007_2026_1527_MOESM1_ESM.zip › same_data/abc3/val_batch0_pred.jpg]

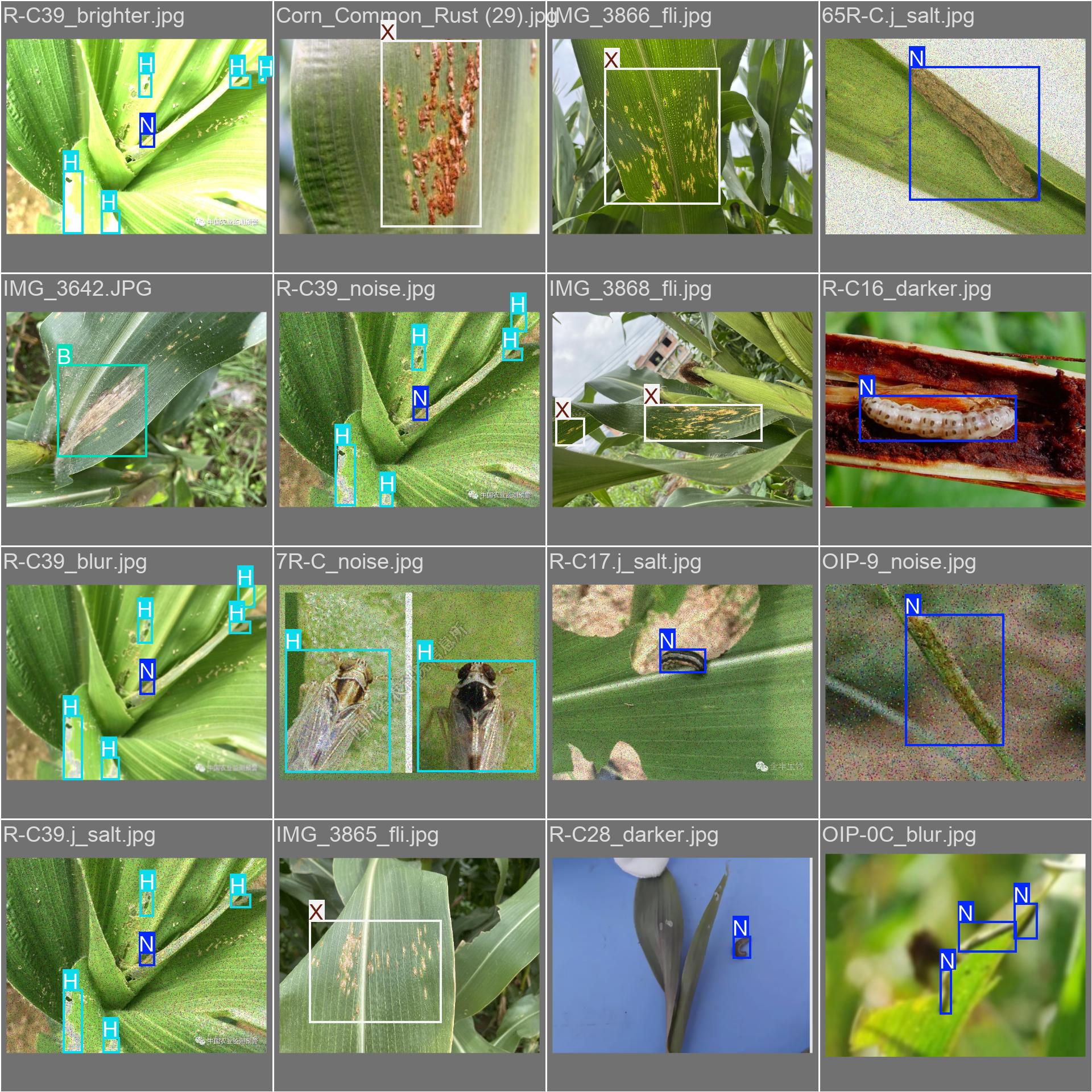

Supplement: Supplementary file 1 — Supplementary Material 1. [file 13007_2026_1527_MOESM1_ESM.zip › same_data/abc3/val_batch1_labels.jpg]

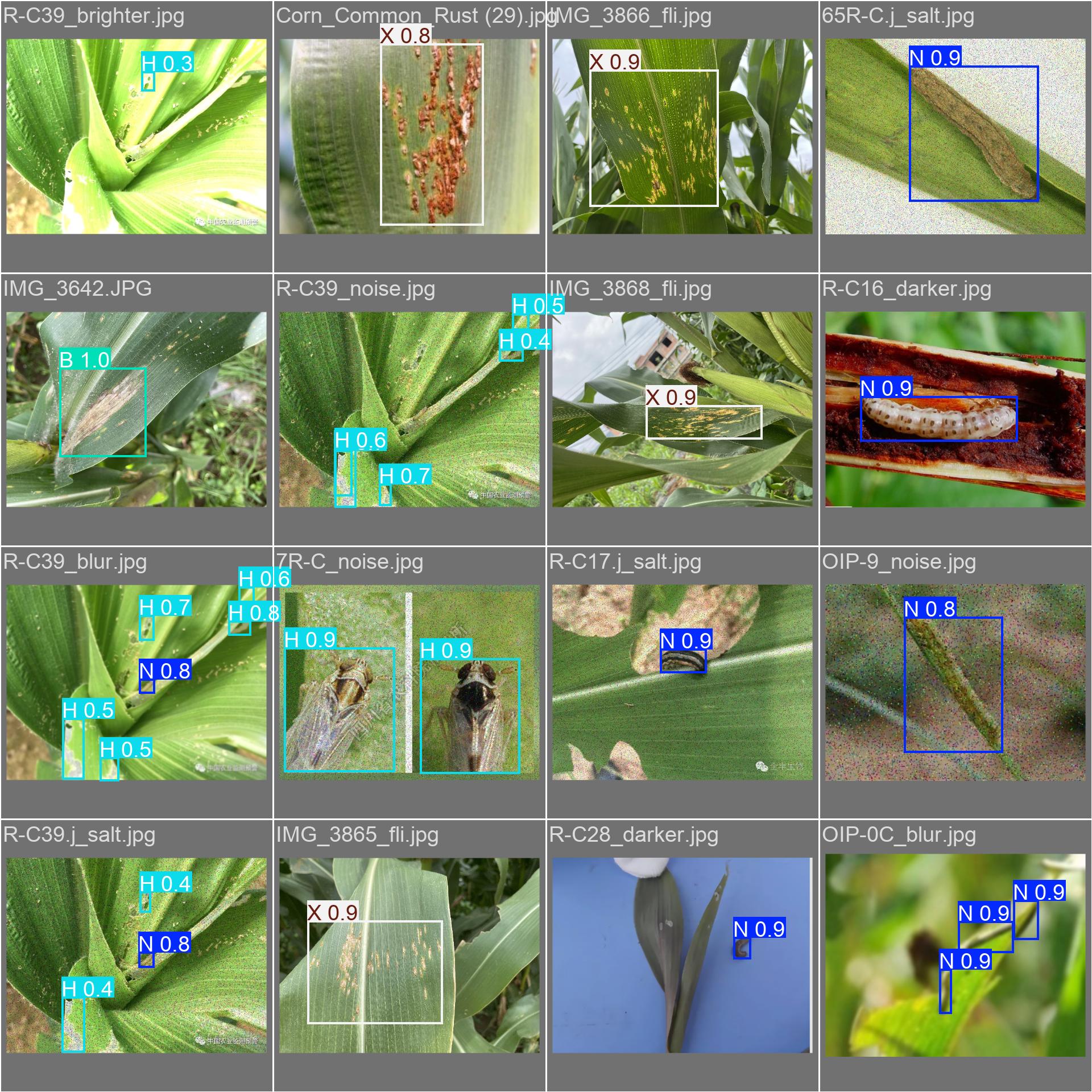

Supplement: Supplementary file 1 — Supplementary Material 1. [file 13007_2026_1527_MOESM1_ESM.zip › same_data/abc3/val_batch1_pred.jpg]

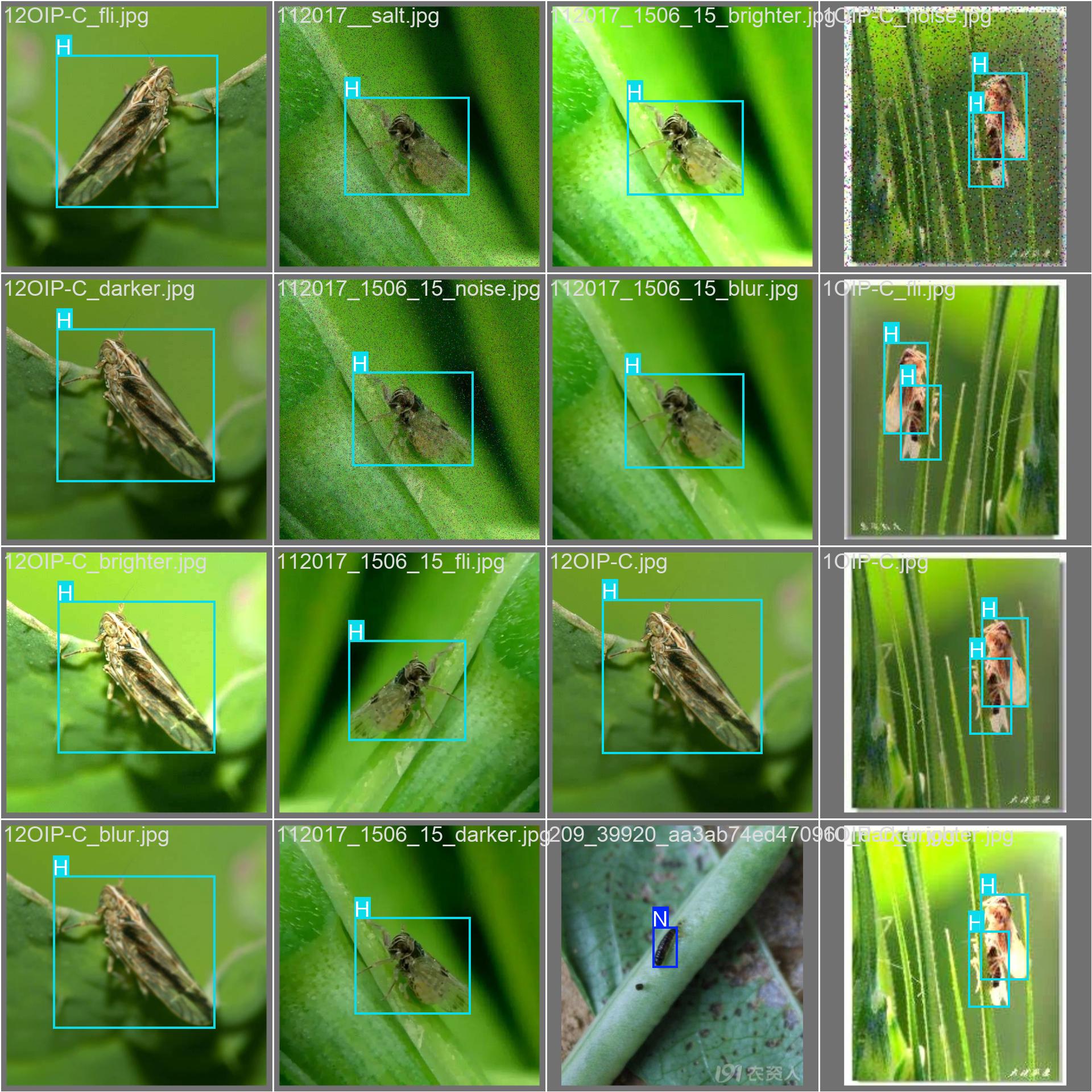

Supplement: Supplementary file 1 — Supplementary Material 1. [file 13007_2026_1527_MOESM1_ESM.zip › same_data/abc3/val_batch2_labels.jpg]

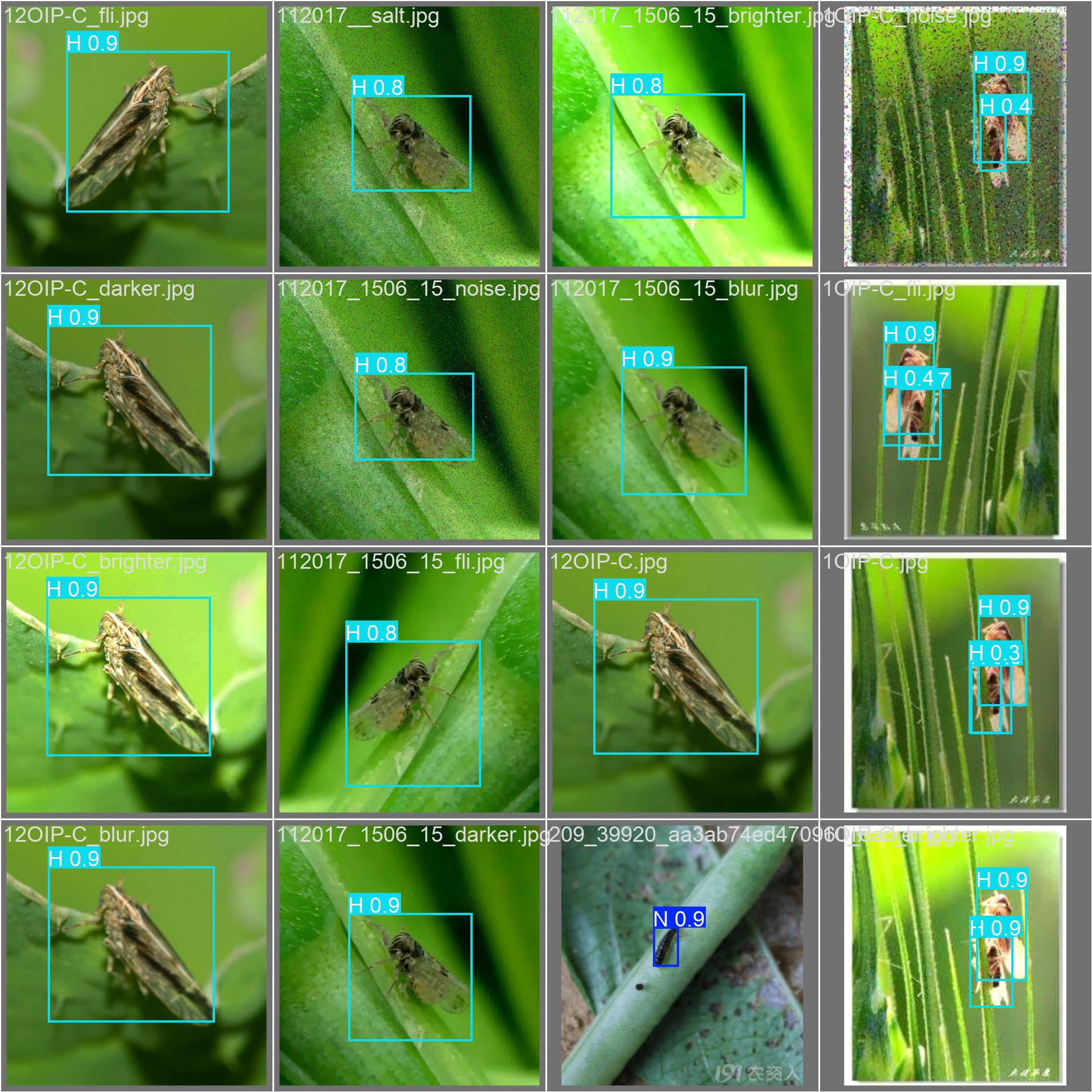

Supplement: Supplementary file 1 — Supplementary Material 1. [file 13007_2026_1527_MOESM1_ESM.zip › same_data/abc3/val_batch2_pred.jpg]

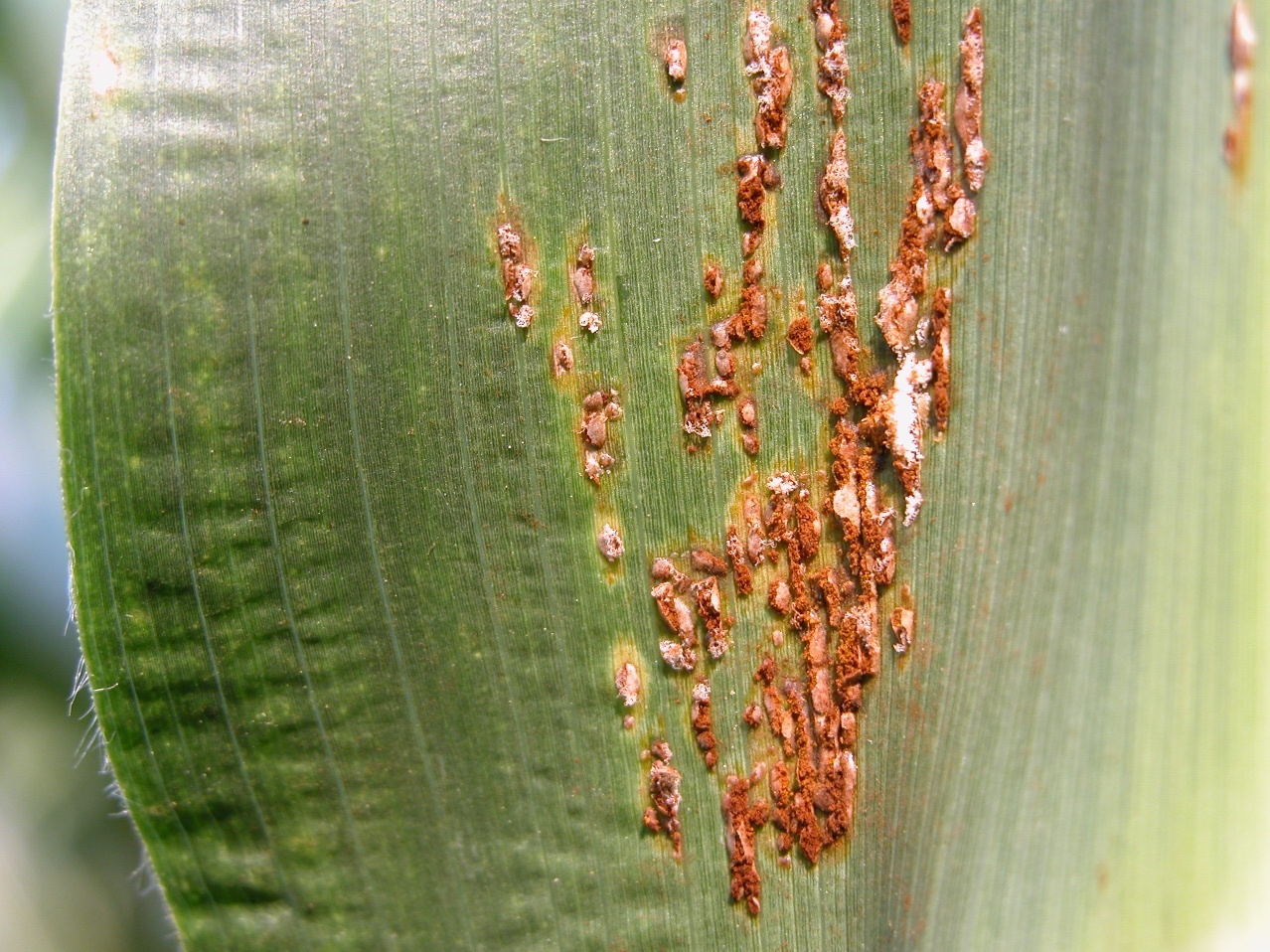

Supplement: Supplementary file 1 — Supplementary Material 1. [file 13007_2026_1527_MOESM1_ESM.zip › same_data/Corn_Common_Rust (28).jpg]

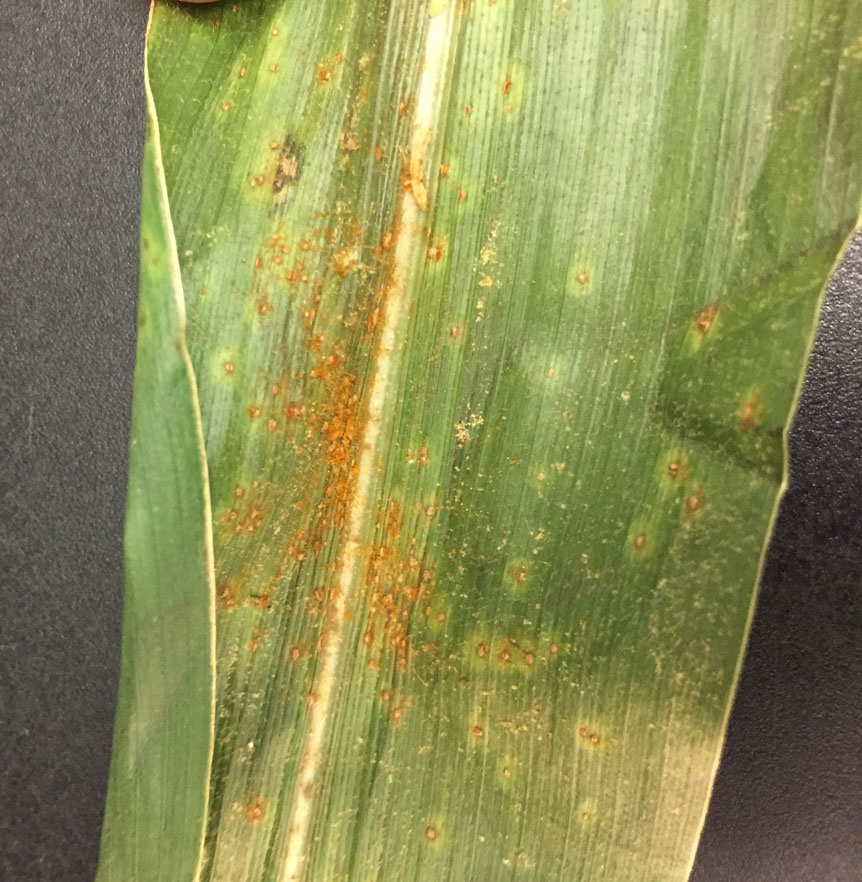

Supplement: Supplementary file 1 — Supplementary Material 1. [file 13007_2026_1527_MOESM1_ESM.zip › same_data/Corn_Common_Rust (57).jpg]

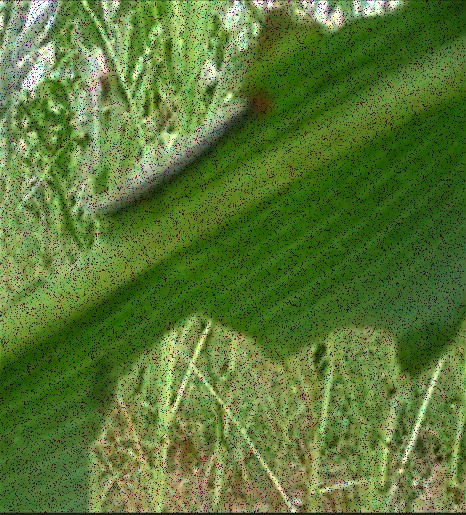

Supplement: Supplementary file 1 — Supplementary Material 1. [file 13007_2026_1527_MOESM1_ESM.zip › same_data/[~19]9K_salt.jpg]
